# Deprescribing psychotropic medicines for behaviours that challenge in people with intellectual disabilities: a systematic review

**DOI:** 10.1186/s12888-022-04479-w

**Published:** 2023-03-28

**Authors:** Danielle Adams, Richard P. Hastings, Ian Maidment, Chetan Shah, Peter E. Langdon

**Affiliations:** 1 Centre for Educational Development, Appraisal and Research, University of Warwick, Coventry, CV4 8UW UK; 2 School of Life and Health Sciences, Aston University, Birmingham, B4 7ET UK; 3 Pharmacy Department, Hertfordshire Partnership University NHS Foundation Trust, Hertfordshire, WD7 9FB UK; 4 Coventry and Warwickshire Partnership NHS Trust, Coventry, CV6 6NY UK; 5 Herefordshire and Worcestershire Health and Care NHS Trust, Worcester, WR5 1JR UK

**Keywords:** Deprescribing, Intellectual disabilities, Psychotropic medication, Behaviours that challenges

## Abstract

**Background:**

Clear evidence of overprescribing of psychotropic medicines to manage behaviours that challenges in people with intellectual disabilities has led to national programmes within the U.K. such as NHS England’s STOMP to address this. The focus of the intervention in our review was deprescribing of psychotropic medicines in children and adults with intellectual disabilities. Mental health symptomatology and quality of life were main outcomes.

**Methods:**

We reviewed the evidence using databases Medline, Embase, PsycINFO, Web of Science, CINAHL and Open Grey with an initial cut-off date of 22nd August 2020 and an update on 14th March 2022. The first reviewer (DA) extracted data using a bespoke form and appraised study quality using CASP and Murad tools. The second reviewer (CS) independently assessed a random 20% of papers.

**Results:**

Database searching identified 8675 records with 54 studies included in the final analysis.

The narrative synthesis suggests that psychotropic medicines can sometimes be deprescribed. Positive and negative consequences were reported. Positive effects on behaviour, mental and physical health were associated with an interdisciplinary model.

**Conclusions:**

This is the first systematic review of the effects of deprescribing psychotropic medicines in people with intellectual disabilities which is not limited to antipsychotics. Main risks of bias were underpowered studies, poor recruitment processes, not accounting for other concurrent interventions and short follow up periods. Further research is needed to understand how to address the negative effects of deprescribing interventions.

**Trial registration:**

The protocol was registered with PROSPERO (registration number CRD42019158079)

**Supplementary Information:**

The online version contains supplementary material available at 10.1186/s12888-022-04479-w.

## Background

Intellectual disabilities are a group of diverse developmental conditions characterised by lower intellectual functioning (usually an IQ of less than 70), and significant impairments of social or adaptive functioning, with an associated onset during childhood [1]. It is relatively common for people with intellectual disabilities to develop behaviours that challenge, with a prevalence of around 10–18% in individuals accessing educational, health or social care services [2–4]. Behaviours that challenge - defined as culturally abnormal behaviour, placing a person at risk of harm to themselves and others - can significantly affect engagement with community amenities due to their duration, intensity, or frequency [5]. These behaviours can include aggression, self-harm, withdrawal, and disruptive or destructive behaviour, including behaviours which may bring the person into contact with the criminal justice system [6].

Prescribing psychotropic medications to treat mental illness may be clinically appropriate for individuals with intellectual disabilities [7, 8]. However, no psychotropic medicines have marketing authorisations for behaviours that challenge in the absence of mental health conditions, except for the short-term use of risperidone and haloperidol for behavioural and psychological effects of dementia. Despite this, behaviours that challenge are independently associated with increased use of psychotropic medication [4, 9]. Psychotropics, particularly if used over a long period of time without adequate review and monitoring, can cause significant harm including: anticholinergic burden, tardive dyskinesia [10, 11], weight gain, and development of metabolic syndrome increasing morbidity and mortality [12]. Therefore, reducing the use of psychotropic medicines for individuals with intellectual disabilities and behaviours that challenge is indicated for reasons of health and quality of life, in addition to being a current policy priority [13, 14].

The purpose of the present paper is to report findings from a systematic review addressing the following question: What are the effects of deprescribing psychotropic medicines as a part of a care pathway or treatment plan for people of all ages with intellectual disabilities and behaviours that challenge? A previous systematic review of deprescribing psychotropic medicines in adults with intellectual disabilities was restricted to antipsychotic medicines involving databases searched between 1st January 1990 and 1st March 2016 [15]. Our review extends this evidence base by including all psychotropic medicines used with children or adults and including research since 2016.

## Methods

The review was conducted and reported in accordance with the Preferred Reporting Items for Systematic Reviews and Meta-Analyses (PRISMA) guidelines 2020. The protocol was registered with the international database of prospectively registered systematic reviews in health and social care (PROSPERO) (registration number CRD42019158079).

### Selection criteria

The eligibility criteria were developed in accordance with the Population, Intervention, Comparator, Outcome (PICO) framework. The population were people with intellectual disabilities prescribed psychotropic medicines of any class for the management of behaviours that challenge. Studies that included adults or children with intellectual disabilities were included. Studies that included fewer than 50% of participants with intellectual disabilities or where data relating to those participants with intellectual disabilities were not reported separately were excluded. The focus of the intervention had to be deprescribing of psychotropic medicines. Studies conducted in both inpatient settings and community settings were eligible for inclusion. Community settings included residential care as well independent living supported by paid or unpaid carers. The primary outcomes were changes in behaviours that challenge and secondary outcomes were changes in quality of life measures or other outcomes such as mental health symptomatology (see Table 1).

**Table 1:** Secondary outcomes

Number and frequency of hospital admissions	
Number and frequency of referrals to intensive nursing support	
Number of placement breakdowns	
Number of dose increases or dose decreases of psychotropic medicines	
Number of new psychotropic medicines initiated, or psychotropic medicines withdrawn	
Frequency, severity, or impact of behaviour that challenges	
Number of hours of social care support	
Estimates of costs associated with deprescribing approaches	
Changes in physical health parameters	

Any experimental, quasi experimental, observational, or case report study reporting relevant quantitative data was included. For the synthesis, studies were grouped according to study design: randomised controlled trials, other comparison designs, pre post studies, longitudinal studies, and descriptive case reports. Studies reporting both individual participant-level data and summary data estimates were included. There were no restrictions on language or date of publication. We did not include abstracts and conference presentations.

### Search strategy

Six electronic databases were searched: Medline, Embase, PsycINFO, Web of Science, CINAHL and Open Grey by the lead reviewer (DA) with a cut-off date of 21st August 2020 (see Table 2 for search strategy). Update searches were carried out on 14th March 2022 with one further case study identified for inclusion.

**Table 2:** Search strategy for medline. Search terms were grouped by (1) “intellectual disabilities, (2) “psychotropic medication”, and (3) “deprescribing”, along with their synonyms. Search terms from within each of groups 1, 2 and 3 were separated using the Boolean operator “OR”. Search terms from groups 1, 2 and 3 were then combined using the Boolean operator “AND”. An example of the full search can be seen in Table 1 below

***1*** ID OR LD OR PMLD OR PIMD OR PDD OR ASD OR autis* OR autism OR Asperger* OR angelman OR PDD NOS OR FASD OR neurofibromatosis OR hypothyroid* OR phenylketonuria OR rubinstein-taybi OR digeorge OR lesch-nyhan OR SEN OR SEND OR DD OR handicap* OR disab* OR (intellectual* adj1 disab*) OR (learning adj1 disab*) OR (intellectual* adj1 deficien*) OR (intellectual* adj1 impair*) OR (learning adj1 difficult*) OR (learning adj1 deficien*) OR (learning adj1 impair*) OR (mental* adj1 retard*) OR (mental* adj1 deficien*) OR (mental* adj1 handicap*) OR (intellectual adj1 developmental adj1 disab*) OR (intellectual adj1 development adj1 disorder*) OR (down* adj1 syndrome) OR (fragile adj1 x adj1 syndrome) OR (fragile adj1 x) OR (william adj1 syndrome) OR (angelman adj1 syndrome) OR (profound* adj2 multiple adj1 learning adj1 disab*) OR (profound* adj1 intellectual* adj1 multiple adj1 disab*) OR (Rett adj1 syndrome) OR (overgrowth adj syndrome) OR (Asperger* adj1 syndrome) OR (autis* adj1 spectrum adj1 disorder) OR (pervasive adj1 developmental* adj1 disorder*) OR (pervasive adj1 developmental* adj1 disorder* adj2 otherwise specified) OR (f?etal adj1 alcohol adj1 syndrome) OR (f?etal adj1 alcohol) OR (prenatal adj1 alcohol adj1 exposure) OR (velocardiofacial adj1 syndrome) OR (klinefelter adj1 syndrome) OR (childhood adj1 disintegrative adj1 disorder) OR (smith adj1 magenis) OR (cri adj1 du adj1 chat) OR (cornelia adj1 de adj1 lange) 36 (de adj1 lange) OR (genetic adj1 disorder*) OR (static adj1 encephalopathy) OR (complex adj1 need*) OR (special adj1 education* adj1 need*) OR (special adj1 need*) OR (special adj1 education* adj1 need* adj2 disabilit*) OR (special adj1 need* adj2 disabilit*) OR (developmental adj1 disabilit*) OR (neurodevelopmental adj1 disorder*) OR (developmental adj1 disorder*) OR (neurodevelopmental adj1 disabilit*) OR (development* adj1 delay*) OR (development* adj1 difficult*) OR (developmental* adj1 impair*) OR (abnormal* adj1 develop*) OR (prader adj1 willi adj1 syndrome) ***2*** psychotropic* OR antidepressant* OR anti-depressant* OR antipsychotic* OR anti- psychotic*or anticonvulsant* OR anti-convulsant* OR antimanic* OR anti-manic* OR antiepileptic* OR anti-epileptic* OR hypnotic* OR SSRI* OR anxiolytic* OR benzodiazepine* OR neuroleptic* OR alprazolam OR fludiazepam OR camazepam OR nordazepam OR etizolam OR clotiazepam OR cloxazolam OR tofisopam OR bentazepam OR loprazolam OR zentiva OR lormetazepam OR dormagen OR niravam OR xanax OR medazepam OR potassium clorazepate OR oxazepam OR bromazepam OR chlordiazepoxide OR chlordiazachel OR libritabs OR lygen OR librium OR oxazepam OR ketazolam OR prazepam OR halazepam OR pinazepam OR adinazolam OR serax OR zaxopam OR emylcamate OR mebutamate OR meprobamate OR benzoctamine OR amosene OR bamate OR equanil OR mepriam OR meprospan OR miltown OR neuramate OR tranmep OR hydroxyzine OR captodiame OR mephenoxalone OR gedocarnil OR etifoxine OR fabomotizole OR atomoxetine OR strattera OR guanfacine OR intuniv OR tenex OR amfetamine OR metamfetamine OR pemoline OR fencamfamine OR modafinil OR fenozolone OR (fenetylline OR armodafinil OR solriamfetol OR caffeine OR propentofylline OR meclofenoxate OR pyritinol OR piracetam OR deanol OR fipexide OR citicoline OR acetylcarnitine OR aniracetam OR idebenone OR prolintane OR pipradrol OR pramiracetam OR adrafinil OR vinpocetine OR tetramethylglycoluril OR phenibut OR oxiracetam OR pirisudanol OR linopirdine OR nizofenone OR dexmethylphenidate OR methylphenidate OR ritalin OR concerta OR delmosart OR equasym OR matoride OR medikinet OR xaggitin OR xenidate OR adhansia OR aptensio OR cotemplar OR daytrana OR dexmethylphenidate OR focalin OR jornay OR metadate OR methylin OR quillichew OR quillivant OR dexamfetamine OR amfexa OR elvanse OR lisdexamfetamine OR vyvanse carbamazepine OR tegretol OR carbagen OR carbatrol OR carnexiv OR epitol OR equetro OR hetrazan OR teril OR eslicarbazepine OR valproate OR belvo OR depacon OR depakene OR stavzor OR depakote OR epilim OR episenta OR epival OR valpromide OR aminobutyric acid OR progabide OR phenytoin OR ethotoin OR mephenytoin OR fosphenytoin OR paramethadione OR trimethadione OR ethadione OR ethosuximide OR phensuximide OR mesuximide OR diazepam OR valium OR dizac OR qpam OR diastat OR mysoline OR primidone OR zonisamide OR zonegran OR vigabatrin OR sabril OR vigadrone OR rufinamide OR inovelon OR banzel OR tiagabine OR gabitril OR topamax OR topiramate OR qsymia OR qudexy OR trokendi OR lamotrigine OR lamictal OR levetiracetam OR keppra OR desitrend OR elepsia OR spritam OR phenobarb* OR methylphenobarbital OR barbexaclone OR metharbital OR oxcarbazepine OR oxtellar OR trileptal OR brivaracetam OR briviact OR clonazepam OR rivotril OR klonopin OR sultiame OR phenacemide OR felbamate OR pheneturide OR zonisamide OR stiripentol OR lacosamide OR cannabidiol OR carisbamate OR beclamide OR retigabine OR perampanel OR clobazam OR onfi OR sympazan OR frisium OR perizam OR tapclob OR zacco OR pentobarbital OR amobarbital OR butobarbital OR bartbital OR aprobarbital OR secobarbital OR talbutal OR vinylbital OR vinbarbital OR cyclobarbital OR heptabarbital OR reposal OR methohexital OR hexobarbital OR thiopental OR etallobarbital OR allobarbital OR proxibarbal OR choral hydrate OR choralodol OR (dichloralphenazone OR paraldehyde OR lorazepam OR ativan OR loraz OR temazepam OR restoril OR temaz OR nitrazepam OR mogadon OR flurazepam OR flunitrazepam OR estazolam OR midazolam OR brotizolam OR quazepam OR loprazolam OR doxefazepam OR cinolazepam OR remimazolam OR glutethimide OR methyprylon OR pyrithyldione OR dalmane OR triazolam OR zopiclone OR zimovane OR zolpidem OR stilnoct OR zolpimist OR tovalt OR intermezzo OR edluar OR ambien OR zaleplon OR sonata OR ramelteon OR tasimelteon OR melatonin OR circadin OR slenyto OR eszopiclone OR lunesta OR methaqualone OR clomethiazole OR bromisoval OR carbromal OR scopolamine OR propiomazine OR triclofos OR hexapropymate OR ethchlorvynol OR bromides OR apronal OR valnoctamide OR methylpentynol OR niaprazine OR dexmedetomidine OR suvorexant OR lyrica OR pregabalin OR lecaent OR alaxid OR alzain OR gabapentin OR gralise OR horizant OR neurontin OR lecomig OR lithium OR camcolit OR liskonum OR eskalith OR lithane OR lithobid OR lithonate OR lithobid OR benperidol OR anquil OR chlorpromazine OR largactil OR promapar OR sonazine OR thorazine OR asenapine OR secuado OR saphris OR sycrest OR flupentixol OR depixol OR psytixol OR flupenthixol OR clopenthixol OR chlorprothixene OR tiotixene OR loxapine OR adasuve OR loxitane OR levomepromazine OR levoprome OR promazine OR sparine OR acepromazine OR cyamemazine OR chlorproethazine OR triflupromazine OR vesprin OR pericyazine OR dixyrazine OR thiopropazate OR acetophenazine OR thioproperazine OR butaperazine OR perazine OR piperazine OR thioridazine OR mesoridazine OR pipotiazine OR periciazine OR perphenazine OR promethazine OR sominex OR phenergan OR trilafon OR pimozide OR orap OR fluspirilene OR penfluridol OR prochlorperazine OR compazine OR compro OR procomp OR sulpiride OR tiapride OR remoxipride OR sultopride OR trifluoperazine OR stelazine OR zuclopenthixol OR clopixol OR fluphenazine OR modecate OR permitil OR prolixin OR veralipride OR levosulpiride OR amisulpride OR solian OR aripiprazole OR abilify OR aristada OR clozapine OR fazaclo OR versacloz OR denzapine OR clozaril OR zaponex OR lurasidone OR latuda OR iloperidone OR cariprazine OR brexpiprazole OR pimavanserin OR paliperidone OR invega OR xeplion OR trevicta risperidone OR risperdal OR prothipendyl OR mosapramine OR olanzapine OR zyprexa OR zylasta OR clotiapine OR quetiapine OR mintreleq OR brancico OR biquelle OR atrolak OR alalquet OR seroquel OR haloperidol OR haldol OR trifluperidol OR melperone OR moperone OR zotepine OR moperone OR pipamperone OR bromperidol OR benperidol OR droperidol OR fluanisone OR oxypertine OR molindone OR sertindole OR ziprasidone OR buspirone OR buspar OR sertraline OR zoloft OR lustral OR fluoxetine OR prozac OR olena OR sarafem OR selfemra OR paroxetine OR brisdelle OR paxil OR pexeva OR seroxat OR citalopram OR celexa OR lexapro OR cipramil OR fluvoxamine OR faverin OR luvox OR duloxetine OR cymbalta OR drizalma OR escitalopram OR zimeldine OR alaproclate OR etoperidone cipralex OR venlafaxine OR alventa OR amphero OR depefex OR majoven OR politid OR sunveniz OR venaxx OR vencarm OR venladex OR venlalic OR vensir OR venzip OR viepax OR effexor OR desvenlafaxine OR khedezla OR pristiq OR clomipramine OR anafranil OR janimine OR pramine OR dibenzepin OR presamine OR desipramine OR imipramine OR tofranil OR opipramol OR protriptyline OR iprindole OR melitracen OR butriptyline OR amoxapine OR dimetacrine OR amineptine OR maprotiline OR quinupramine OR amitriptyline OR amitid OR amitril OR elavil OR endep OR dosulepin OR dothiepin OR doxepine OR mianserin OR trazodone OR oxitriptan OR nomifensine OR nefazodone OR minaprine OR bifemelane OR viloxazine OR oxaflozane OR bupropion OR medifoxamine OR tianeptine OR pivagabine OR levomilnacipran OR milnacipran OR gepirone OR duloxetine OR vilazodone OR molipaxin OR hyperici herba OR esketamine OR desyrel OR oleptro OR trialodine OR trimipramine OR surmontil OR lofepramine OR thioridazine OR melleril OR aventyl OR pamelor OR nortriptyline OR tranylcypromine OR advanz OR parnate OR phenelzine OR nardil OR nialamide OR iproniazide OR iproclozide OR isocarboxazid OR marplan OR moclobemide OR manerix OR toloxatone OR reboxetine OR edronax OR mirtazapine OR remeron OR zispin OR clomipramine OR vortioxetine OR brintellix OR trintellix OR tryptophan OR agomelatine OR valdoxan OR modafinil OR provigil OR armodafinil OR nuvigil OR norpramin OR pertofrane OR pamelor OR aventyl OR vivactil OR asendin OR ludikomil OR serzone OR zyban OR wellbutrin OR forfivo OR aplenzin OR contrave OR khedezla OR pristiq OR viibryd OR cylert OR focalin OR gemonil OR dilantin OR diphenylan OR phenytek OR peganone OR cerebyx OR mesantoin OR paradione OR tridione OR zarontin OR milontin OR aptiom OR phenurone OR felbatol OR zonegran OR diacomit OR vimpat OR fycompa OR antepar OR bryrel OR multifuge OR vermidol OR serentil OR inapsine OR moban OR geodon OR taractan OR adasuve OR loxitane OR fanapt OR rexulti OR nuplazid OR serax OR zaxopam OR centrax OR atarax OR orgatrax OR vistaril OR bamate OR amosene OR equanil OR mepriam OR meprospan OR miltown OR neuromate OR tranmep OR nembutal OR sarisol OR butabarb OR butalan OR butisol OR buticaps OR seconal OR prosom OR halcion OR rozerem OR triclos OR placidyl OR precedex OR belsomra OR fetzima OR savella OR (valproic adj1 acid) OR (psychotropic adj1 medicine*) OR (psychotropic adj1 medication*) OR (psychotropic adj1 drug*) OR (psychotropic adj1 agent*) OR (antidepressant adj1 medicine*) OR (antidepressant adj1 medication*) OR (antidepressant adj1 drug*) OR (antidepressant adj1 agent*) OR (anti-depressant adj1 medicine*) OR (anti-depressant adj1 medication*) OR (anti- depressant adj1 drug*) OR (anti-depressant adj1 agent*) OR (antipsychotic adj1 medicine*) OR (antipsychotic adj1 medication*) OR (antipsychotic adj1 drug*) OR (antipsychotic adj1 agent*) OR (anti-psychotic adj1 medicine*) OR (anti-psychotic adj1 medication*) OR (anti-psychotic adj1 drug*) OR (anti-psychotic adj1 agent*) OR (neuroleptic adj1 medicine*) OR (neuroleptic adj1 medication*) OR (neuroleptic adj1 drug*) OR (neuroleptic adj1 agent*) OR (anticonvulsant adj1 medicine*) OR (anticonvulsant adj1 medication*) OR (anticonvulsant adj1 drug*) OR (anticonvulsant adj1 agent*) OR (anti-convulsant adj1 medicine*) OR (anti- convulsant adj1 medication*) OR (anti-convulsant adj1 drug*) OR (anti-convulsant adj1 agent*)) OR ((antimanic adj1 medicine*) OR (antimanic adj1 medication*) OR (antimanic adj1 drug*) OR (antimanic adj1 agent*) OR ((anti-manic adj1 medicine*) OR (anti-manic adj1 medication*) OR (anti-manic adj1 drug*) OR (anti-manic adj1 agent*)) OR ((antiepileptic adj1 medicine*) OR (antiepileptic adj1 medication*) OR (antiepileptic adj1 drug*) OR (antiepileptic adj1 agent*) OR (anti-epileptic adj1 medicine*) OR (anti-epileptic adj1 medication*) OR (anti-epileptic adj1 drug*) OR (anti-epileptic adj1 agent*) OR (ADHD adj1 medication*) OR (ADHD adj1 medicine*) OR (selective adj1 serotonin adj1 reuptake adj1 inhibitor*) OR (serotonin adj2 norepinephrine adj1 reuptake adj1 inhibitor*) OR (serotonin adj2 noradrenaline adj1 reuptake adj1 inhibitor*) OR (ethyl adj1 loflazepate) OR (lavandulae adj1 aetheroleum) OR (amino adj1 valeric adj1 acid) OR (valerianae adj1 radix). ***3*** discontin* or deprescrib* or de-prescrib* OR deprescrip* OR polypharmacy OR taper* OR (medication adj5 withdraw*) OR (medicine* adj5 withdraw*) OR (drug* adj5 withdraw*) OR (medicine* adj5 discontin*) OR (medication adj5 discontin*) OR (drug* adj5 discontin*) OR (medicine* adj5 reduc*) OR (medication adj5 reduc*) OR (dose* adj5 reduc*) OR (inappropriate adj2 prescription*) OR (inappropriate adj2 prescribing) OR (medicine* adj5 decreas*) OR (medication adj5 decreas*) OR (dose* adj5 decreas*) ***4*** **1 AND 2 AND 3** Limits: Human Studies only	

References were imported into an EndNote library, removing duplicates using both the software function and a manual check. Forwards and backwards reference searching of included papers was also conducted to track citations after the initial search and again after the updated search. Four key researchers, identified as having published several studies in this field over the last 10 years, were contacted to identify any further studies. Trial registries were not searched.

### Data selection

Following the removal of duplicates, the titles and abstracts of the remaining records were reviewed by the primary reviewer (DA) against the eligibility criteria. A second reviewer (CS) undertook an independent screening of a random sample of 20% of abstract/title records. Following this, the remaining papers were subjected to full text screening by DA with another random sample of 20% of full texts screened by CS. Near perfect agreement was achieved for title /abstract screening (*k* = 0.86) and for full text screening (*k* = 0.81). Disagreements were resolved by discussion together with a third arbiter (PL). Automation tools were not used.

### Data extraction

A bespoke data extraction form, consisting of six main categories with sub-categories, was developed to extract relevant data. The primary reviewer (DA) extracted data from all included studies and the second reviewer (CS) conducted independent data extraction for a random selection of 20% of studies. No formal agreement statistics were calculated but a high level of agreement was achieved.

### Quality appraisal

Following data extraction, studies were individually appraised for risk of bias by DA using the appropriate tool from the Critical Appraisals Skills Programme Tools [16] each of which consist of ten questions to assess internal and external validity. Case reports and case series were quality appraised by using tool developed by Murad et al. [17]. The second independent reviewer (CS) quality appraised a random sample of 20% of the included studies, full agreement was reached.

### Synthesis methods

Due to the heterogeneity of research design and the variability in participants, interventions and settings of included studies, a narrative approach was selected to synthesise the data, summarising the current evidence base in relation to the review question. Grouping the studies according to study design, the narrative synthesis focused on patterns in the direction and size of the effects of the deprescribing interventions and exploring relationships within and between studies and identifying factors that may help us to understand differences in reported findings [18].

## Results

**Fig. 1:**
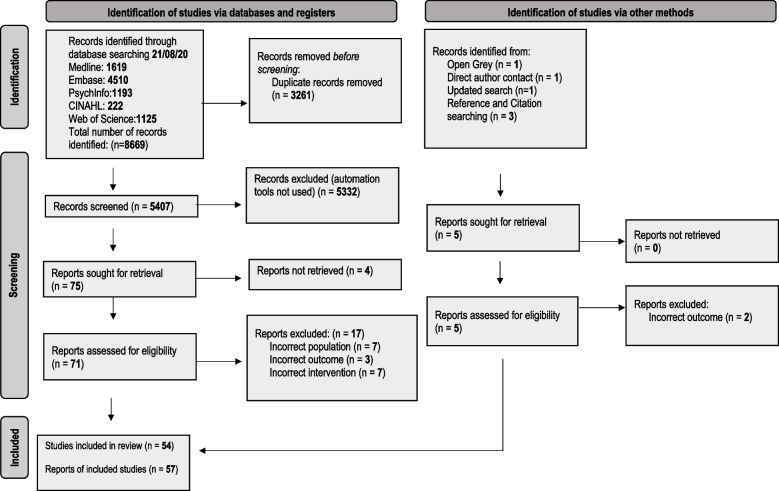
PRISMA 2020 flow diagram for the deprescribing of psychotropic medicines in people with intellectual disabilities prescribed for behaviour that challenges: a systematic review [19]

We identified 8675 records, and 57 reports relating to 54 studies met our eligibility criteria and were included in the review. This is reported in the PRISMA flow diagram (Fig. 1).

Studies excluded at full text review together with reasons were recorded and listed in Table 3. Summary tables of extracted data for included studies are reported in Tables 4,  5, 6, and 7. A summary table of quality appraisal for included studies is reported in Table  8.

**Table 3:** List of excluded studies at full text screening with reasons. The following studies were excluded during screening of full text papers as they did not meet the eligibility criteria for the reasons listed below

IPP	Incorrect Patient Population
IO	Incorrect Outcome
II	Incorrect Intervention
FTPU	Full Text Paper Unavailable
**EXCLUDED STUDY**	**REASON FOR EXCLUSION**
**Alblowi, M. A., and F. D. Alosaimi. “Tardive Dyskinesia Occurring in a Young Woman after Withdrawal of an Atypical Antipsychotic Drug.”** ***Neurosciences*** **20.4 (2015): 376–79.**	IPP
**Baglio, Christopher. “Evidence and Impact of Expectancies Associated with Psychotropic Medication Reductions in Persons with Mental Retardation.”** ***ED.D.Dissertations. 15.*** **(2010).**	IO
**Branford, D. “Antipsychotic Drugs in Learning Disabilities (Mental Handicap).”** ***Pharmaceutical Journal*** **258.6936 (1997): 451–56.**	II
**Briggs, R. “Monitoring and Evaluating Psychotropic Drug Use for Persons with Mental Retardation: A Follow-up Report.”** ***American Journal of Mental Retardation*** **93.6 (1989): 633–9.**	FTPU
**Campbell, M., et al. “Tardive and Withdrawal Dyskinesia in Autistic Children: A Prospective Study.”** ***Psychopharmacology Bulletin*** **24.2 (1988): 251–55.**	FTPU
**Campbell, M., et al. “Neuroleptic-Related Dyskinesias in Autistic Children: A Prospective, Longitudinal Study.”** ***Journal of the American Academy of Child & Adolescent Psychiatry*** **36.6 (1997): 835–43.**	IPP
**Connor, D. F., S. Benjamin, and K. R. Ozbayrak. “Case-Study - Neuroleptic Withdrawal Dyskinesia Exacerbated by Ongoing Stimulant Treatment.”** ***Journal of the American Academy of Child and Adolescent Psychiatry*** **34.11 (1995): 1490–94.**	IPP
**Connor, D. F., and T. J. McLaughlin. “A Naturalistic Study of Medication Reduction in a Residential Treatment Setting.”** ***Journal of Child & Adolescent Psychopharmacology*** **15.2 (2005): 302–10.**	IPP
**Davies, S. J., et al. “Discontinuation of Thioridazine in Patients with Learning Disabilities: Balancing Cardiovascular Toxicity with Adverse Consequences of Changing Drugs.”** ***BMJ*** **324.7352 (2002): 1519–21.**	IPP
**Deb, S., and W. Fraser. “The Use of Psychotropic Medication in People with Learning Disability: Towards Rational Prescribing.”** ***Human Psychopharmacology*** **9.4 (1994): 259–72.**	II
**Deb, S., G. Unwin, and T. Deb. “Characteristics and the Trajectory of Psychotropic Medication Use in General and Antipsychotics in Particular among Adults with an Intellectual Disability Who Exhibit Aggressive Behaviour.”** ***Journal of Intellectual Disability Research*** **59.1 (2015): 11–25.**	II
**Granas, A. G., et al. “Interdisciplinary Medication Review to Improve Pharmacotherapy for Patients with Intellectual Disabilities.”** ***International Journal of Clinical Pharmacy*** **41.6 (2019): 1516–25.**	II
**Hancock, Robert D., et al. “Changes in Psychotropic Drug Use in Long-Term Residents of an Icf/Mr Facility.”** ***American Journal on Mental Retardation*** **96.2 (1991): 137–41.**	II
**Malone, R. P., et al. “Repeated Episodes of Neuroleptic-Related Dyskinesias in Autistic Children.”** ***Psychopharmacology Bulletin*** **27.2 (1991): 113–7.**	FTPU
**Malone, R. P., et al. “Risperidone Treatment in Children and Adolescents with Autism: Short- and Long-Term Safety and Effectiveness.”** ***Journal of the American Academy of Child & Adolescent Psychiatry*** **41.2 (2002): 140–7.**	IO
**Okorie, E., and C. Connaughton. “Antipsychotic Prescribing in a Residential Facility for Clients with Learning Disabilty.”** ***British Journal of Developmental Disabilities*** **57.2 (2011): 11722.**	II
**Perez, C. A., S. S. Garcia, and R. D. Yu. “Extrapyramidal Symptoms as a Result of Risperidone Discontinuation During Combination Therapy with Methylphenidate in a Pediatric Patient.”** ***Journal of Child and Adolescent Psychopharmacology*** **26.2 (2016): 182.**	IPP
**Silva, R. R., et al. “Haloperidol Withdrawal and Weight Changes in Autistic Children.”** ***Psychopharmacology Bulletin*** **29.2 (1993): 287–91.**	FPTU
**Sovner, R. “Thioridazine Withdrawal-Induced Behavioral Deterioration Treated with Clonidine: Two Case Reports.”** ***Mental Retardation*** **33.4 (1995): 221–5**	IPP
**Tiihonen, Jari. “Fatal Agranulocytosis 4 Years after Discontinuation of Clozapine.”** ***The American Journal of Psychiatry*** **163.1 (2006): 161.**	II
**Wrein, D. “Understanding the Role of Care Staff in Supporting Individuals with an Intellectual Disability Who Take Psychotropic Medication.”** ***Prof Doc Thesis University of East London School of Psychology*** **(2019).**	IO

**Table 4:** Summary of included randomised controlled trials

Author/Year, Country	Study Design	Participants (n, age, gender, ethnicity, level of intellectual disabilities)	Setting	Intervention (including medication that was targeted, Duration of deprescribing intervention and length of follow up)	Outcome Measures	Summary of Findings
Research Units on Pediatric Psychopharmacology Autism Network. 2005 USA [20]	Randomised Controlled Trial (RCT)	n: 38 mean age: 9 years old (5 to 17) gender: 87% male ethnicity: not reported ID = mild 8 (21%) moderate 6 (16%) severe 7 (18%) profound 6 (16%) plus 4 incomplete data and remainder borderline or above average IQ	Community	**Intervention:** Part of a two-stage study. Maintenance dose reduced by 25% per week in the experimental group. Control group continued risperidone. **Medication:** risperidone **Duration:** reduced over 3 weeks **Length of follow up**: 5 weeks after planned discontinuation	ABC CGI	84% completed discontinuation After 32 participants completed the study, trial was stopped. Relapse rates were 62.5% for gradual placebo substitution and 12.5% for continued risperidone; difference was found to be statistically significant.
Ahmed et al. 2000 U.K. [21]	RCT	*n* = 56 (Includes participants also reported in Smith et al., 2002 [22]) mean age = 43(20 to 78) gender: 48% male ethnicity: not reported ID = incomplete data	45% NHS hospitals 9% NHS Community unit 46% Community residential homes	**Intervention:** Thirty-six participants randomly allocated to the experimental group underwent four, monthly 25% drug reduction stages. There were no planned drug changes for the control group **Medication:** Participants received 12 different antipsychotic drugs, most frequently thioridazine (18 people, 12%), haloperidol (13, 23%) and chlorpromazine (8, 14%). **Duration:** Reduced over 4 months **Length of follow up**: 1 month after planned discontinuation	ABC, DISCUS, weighing scales, direct observation using palm-top Psion 3a portable computers, Number of participants Successfully deprescribed	12 participants (33%) completed full withdrawal 7 participants (19%) achieved and maintained at least a 50% reduction. Drug reduction was associated with increased dyskinesia and higher activity engagement but not increased maladaptive behaviour. Some setting characteristics were associated with drug reinstatement.
de Kuijper et al. 2014 [23] The Netherlands	RCT	n: 98 (Includes participants also reported in de Kuijper et al.,2013 [24], de Kuijper et al. 2014 [25] and de Kuijper et al. 2018 [26]) mean age: 49.8 (15 to 66) gender: 64% male ethnicity: not reported ID: profound 35 (36%) severe 26 (27%) moderate 30 (31%) mild 7 (7.1%)	Community	**Intervention:** Participants underwent 12.5% antipsychotic dose reduction every 2 or 4 weeks **Medication:** 65 pipamperone, 18 haloperidol, 15 risperidone, 8 olanzapine, 7 levomepromazine 1 pimozide **Duration:** Reduced over 14 or 28 weeks **Length of follow up:** 12 weeks after planned discontinuation	Primary outcome: ABC (irritability subscale) Secondary outcomes: other ABC subscales, CGI, SCOPA-AUT, Epworth Sleepiness Scale, AIMS, Barnes, Unified Parkinson’s Disease Rating Scale Physical Health parameters- weight, BP, lipids, waist circumference, pulse, prolactin, testosterone, Number of participants Successfully deprescribed	Of 98 participants, 43 achieved complete discontinuation; at follow-up 7 had resumed use of antipsychotics. Mean ABC ratings improved significantly for those who achieved complete discontinuation and at follow-up for those who had not achieved complete discontinuation. Similar results with respect to most ABC sub-scales, including the ‘irritability’ subscale. No significant differences in improvement of ABC ratings between both discontinuation schedules. Higher ratings of extrapyramidal and autonomic symptoms at baseline associated with less improvement of behavioural symptoms after discontinuation; higher baseline ABC rating predicted higher odds of incomplete discontinuation.
de Kuijper et al. 2013 [24] The Netherlands	RCT **additional reporting of** [23]	(Includes participants also reported in de Kuijper et al.*,* 2014 [23, 24])	’		Fasting glucose, triglycerides, high density lipoproteins, low-density lipoproteins, and total cholesterol in blood; height, weight, and waist circumference and systolic and diastolic blood pressure	Discontinuation of anti-psychotics led to a significant decrease in waist circumference, weight, BMI, and systolic blood pressure. Higher baseline dosage associated with a larger decrease in waist circumference, weight, and BMI in these participants. No significant difference between discontinuation in 14 or 28 weeks. Dosage reductions associated with decrease in weight and BMI, negatively associated with metabolic outcomes such as fasting plasma glucose levels.
de Kuijper et al. 2014 The Netherlands [27]	RCT **additional reporting of** [23]	(Includes participants also reported in de Kuijper et al.*,2013* [24] *and de Kuijper* et al.*,,* 2014 [23])		,	Plasma measurements included prolactin, testosterone (only in male participants), 25-OH vitamin D, PTH, and bone turnover markers, ie, bone alkaline phosphatase (BALP), aminopropeptide type I collagen (PINP), and C-telopeptide type I collagen (CTX).	Both complete discontinuation and dosage reduction led to decrease in prolactin plasma levels and to increase in levels of CTX, the bone resorption marker. Dose reductions associated with a significant decrease in 25-OH vitamin D levels, with less weight loss and higher BMI compared with those who had completely discontinued. More weight loss associated with less difference in baseline/follow-up CTX levels and with less difference in baseline/follow-up 25-OH vitamin D levels.
Hassler et al. 2007 Germany [28]	RCT	n: 39 (Includes 31 participants also reported in de Hassler et al.*,* 2011 [29]) mean age: 36.4 (SD 10.4) gender: 54% male ethnicity: 100% white ID: severe 28 (72%) moderate 9 (23%) mild 2 (5%)	Inpatient	**Intervention:** Random allocation of withdrawal of medication after a 6 week period of open treatment **Medication:** zuclopentixol **Duration**: Sudden discontinuation **Length of follow up:** 12 weeks after discontinuation	Primary outcome: MOAS Secondary outcome: withdrawal symptoms, extrapyramidal signs, vital signs, weight, and routine laboratory tests of prolactin and serum levels of zuclopenthixol were conducted.	The placebo group was associated with more aggressive behaviour as indicated by outcomes observed by external raters.
Hassler et al. 2011 Germany [29]	RCT **additional reporting of** [28]	n: 31 (The participants were also reported in Hassler et al.*,* 2007 [28]) mean age: 38.4 (adults) gender: 55% male ethnicity: 100% white ID = not reported	Inpatient	**Intervention:** Prospective follow up of an RCT in which participants who remained on medication were compared to those participants who discontinued during a 2 year period **Medication:** zuclopentixol **Duration:** Not reported **Length of follow up:** Variable	MOAS DAS CGI-I Body weight	Patients still treated with zuclopentixol after 2 years (*n* = 21) benefitted, compared to the patients who discontinued (*n* = 10) For continually treated patients, no adverse events, side-effects, or treated extrapyramidal symptoms were reported. They lost on average 1.8 kg body weight. Patients who discontinued zuclopentixol on average gained 2.6 kg body weight.
Heistad et al. 1982 USA [30]	RCT	n: 100 mean age: 28.5 (13 to 65) ethnicity: not reported gender: 54% male ID: > 50% profound	Inpatient	**Intervention**: 5 separate arms to assess effect of withdrawal. Details not reported. Patients selected were rank ordered by current drug dose, and one member of each successive pair was randomly assigned to either the drug-placebo or the placebo-drug sequence. **Medication:** thioridazine **Duration:** variable **Length of follow up:** 4 to 5 weeks after discontinuation	Unvalidated adapted behaviour coding system, NOSIE, AIMS	Time-sampling of behaviour showed significant increase in self-stimulation and active negative behaviour and decreased work and life skills while receiving placebo. Most patients’ behaviour was better while on active medication, some showed significant improvement when medication was temporarily discontinued. Favourable long-term progress among those who had medication restored was greater for patients whose behaviour had worsened to the greatest degree during the placebo (discontinuation) trial.
McNamara et al. 2017 U.K. [31]	RCT	*n* = 22 mean age: 43 (21 to 68) gender: 68% male ethnicity: not reported ID = not reported	Community	**Intervention:** Treatment in the intervention group was gradually reduced over a 6-month period and then maintained at the same level for a further 3 months. In the control group, baseline level of medication was maintained throughout the 9-month period. **Medication**: risperidone **Duration:** Reduced over 6 months **Length of follow up**: 6 and 9 months after planned discontinuation	Feasibility outcomes: the Number and proportion of general practices/Community learning disability teams that progressed from initial approach to recruitment of participants and the Number and proportion of recruited participants who progressed through the various stages of the study. Clinical outcomes: MOAS, ABC, PAS-ADD, The ASC, DISCUS, the CSRI, use of other interventions to manage challenging behaviour, use of PRN medication and level of psychotropic medication use.	Of the 22 participants randomised (intervention, *n =* 11; control, *n =* 11), 13 (59%) achieved progression through all four stages of reduction. Follow-up data at 6 and 9 months were obtained for 17 participants (intervention, *n =* 10; and control, *n =* 7; 77% of those randomised). No clinically important changes in participants’ levels of aggression or challenging behaviour reported.
Ramerman et al. 2019 The Netherlands [32]	RCT	n:25 (11 participants also reported in Kuijper et al.*,* 2018 [33] and Ramerman et al.*,*2019 [34]) mean age: 30 gender: 76% male ethnicity: not reported ID: mild 52% moderate 24% severe 24% profound 0%	Inpatient	**Intervention:** In the discontinuation group, Risperidone was gradually replaced by a placebo over 14 weeks, while the control group maintained their existing dosage. **Medication:** risperidone **Duration:** Reduced over 14 weeks **Length of follow up:** 8 weeks after planned discontinuation	ABC CGS-I, SCOPA-AUT, Epworth Sleepiness Scale, AIMS, Barnes, Unified Parkinson’s Disease Rating Scale Physical Health parameters- weight, BP, lipids, waist circumference, pulse, Prolactin, testosterone, Number of participants Successfully deprescribed	In the discontinuation group, 82% completely withdrew from risperidone. No significant change in irritability, compared with the continuation group, although there was Group^a^ Time effects on stereotypical behaviour in favour of the continuation group. Significant Group^a^Time effects were also found for weight, waist, body mass index, prolactin. Levels and testosterone levels, with beneficial effects for the discontinuation group. 2 participants had severe dyskinesia
Smith et al. 2002 U.K. [22]	RCT **additional reporting of** [21]	(Participants also reported in Ahmed et al., 2000 [21])			ABS, ABC DISCUS direct observation	High Yule’s *Q*-value results pre- and post-baseline were found, indicating that clients were highly responsive to staff interaction. Yule’s *Q*-value did not significantly increase following drug withdrawal.

Key: *AIMS* Abnormal Involuntary Movement Scale, *ABC* Aberrant Behavior Checklist, *ABS* Agitated Behaviour Scale, *BARNES* Barnes Akathisia Rating Scale, *BFCRS* Bush-Francis Catatonia Rating Scale, *BP* Blood Pressure, *CARS* Childhood Autism Rating Scale CGAS: Children’s Global Assessment Scale (CGAS) CGI: Clinical Global Impressions, *CSM* Committee on Safety of Medicines, *CPRS* Comprehensive Psychopathological Rating Scale, *DAS* Disability Assessment Schedule, *DISCUS* Dyskinesia Identification System Condensed User Scale, *DISCO* Dyskinesia Identification System-Coldwater, *ECG* Electrocardiogram, *FBC* Full Blood Count, *HbA1c* Glycated Haemoglobin, *Kiddie SAD-PL* Kiddie Schedule for Affective Disorders and Schizophrenia, Present and Lifetyime Version, *LFTs* Liver Function Tests, *MOAS* Modified Overt Aggression Scale, *NOSIE* Nurses’ Observation Scale for Inpatient Evaluation, *PAS-ADD* Psychiatric Assessment Schedule for Adult with Developmental Disability, *PBS* Positive Behaviour Support, *PTH* Parathyroid Hormone, *RAND-36* measure of health related quality of life, *U + Es* Urea and elelctrolytes, *UPDRS* Unified Parkinson’s Disease Rating Scale, *SCOPA-AUT* Scales for Outcomes in Parkinson’s Disease - Autonomic Dysfunction

**Table 5:** Summary of included non-randomised controlled trials

Author/Year, Country	Study Design	Participants (n, age, gender, ethnicity, level of intellectual disabilities)	Setting	Intervention (including medication that was targeted, Duration of deprescribing intervention and length of follow up)	Outcome Measures	Summary of Findings
Aman et al. 1985 New Zealand [35]	Prospective non randomised controlled design	n: 24 mean age: Not reported gender: 71% male ethnicity: not reported ID = severe / profound 100%	Inpatient	**Intervention:** Participants received their full antipsychotic dosage during the first week, a half dosage during the second week, and no medication thereafter for 4 weeks. The control group were not taking psychotropic medicines prior to the study **Medication:** trifluoperazine, thioridazine, periciazine, haloperidol **Duration:** Over one week **Length of follow up**: 8 weeks after discontinuation	DISCO	The antipsychotic medication group was rated as having higher total dyskinesia scores. However, examination of three Group by Time interactions indicated that the symptom scores rose more rapidly for the control group.
Carpenter et al. 1990 USA [36]	Prospective non randomised controlled design	n: 10 mean age: 30 (18 to 53) gender: 90% male ethnicity: 70% black 30% white ID = borderline 10% mild 50% moderate 10% severe 30%	Inpatient	**Intervention:** Reduction of antipsychotic medication by 25–100%,; no medication changes in control group **Medication:** chlorpromazine, Thioridazine, haloperidol **Duration:** Not reported **Length of follow up:** Not reported	Performance on a discrimination task requiring matching of colours presented sequentially on a computer screen	Group that had their medicines deprescribed achieved better scores than control group.
Gerrard et al. 2019 U.K. [37]	Prospective non randomised controlled design	n: 54 (may include participants also reported in Gerrard 2020 [38]) mean age: Not reported gender: 50% male ethnicity: not reported ID = mild 16 (30%) moderate 16 (30%) severe 20 (37%) profound 2 (3%)	Community	**Intervention:** The experimental group were considered for deprescribing with input from specialist PBS team, while the control group underwent unsupported medication challenge. **Medication:** amisulpride 5%, aripiprazole 29%, olanzapine 5%, quetiapine 9%, risperidone 52% **Duration:** Variable **Length of follow up:** Variable	Number of patients who: agreed to initiation of a reduction schedule agreed to subsequent reductions had medication reviews discontinued medication Number of patients restarted on medication Number of patients achieving 25, 50% or 75% reduction Number of patients who had medication increased	There was a significantly higher success rate for medication reduction and discontinuation when PBS assessment and intervention was provided. At each stage of the process, initiating a reduction schedule, there is a difference between the two groups, pointing to greater success with the support of PBS. Complete discontinuation: 60% in PBS group 15% in non PBS group At least 50% reduction: 20% in PBS group, 7% in non PBS group Represcribing or dose increases: 1 person in PBS group 66% In non PBS group
Swanson et al. 1996 USA [39]	Prospective non randomised controlled design	n:80 mean age: 38 gender: 61% male ethnicity: not reported ID = Moderate: 1% Severe: 16% Profound: 80% Unknown: 3%	Inpatient	**Intervention:** antipsychotic dose reduced by 10% every 3 months until discontinued **Medication:** risperidone **Duration:** Variable **Length of follow up**:6 months post discontinuation	DISCUS ABC	Transient increase in average DISCUS score in antipsychotic only group after withdrawal with return to baseline 6 months after discontinuation. In group antipsychotic plus anticonvulsant no change in scores reported. Transient increase in average ABC during antipsychotic withdrawal in those also prescribed anticonvulsants.
Wigal et al. 1993 USA [40]	Prospective non randomised controlled design	n: 56 (may include participants also reported in Wigal et al., 1994 [41]) mean age: 33 gender: 64% male ethnicity: not reported ID = severe/profound 96%	Inpatient	**Intervention:** Medication review and dose reduction programme Four groups compared increase in antipsychotic dose (IN; *n* = 5), no change in antipsychotic dose (NC; *n* = 14), reduction in antipsychotic dose of < 25% (SD; *n* = 21), and reduction in antipsychotic dose of ≥25% (25D, *n* = 16) **Medication:** antipsychotic **Duration**: Variable **Length of follow up**:10 months	DISCUS Proportion of participants with dyskinesia Number of participants discontinuing and decreasing dosage	No difference in DISCUS score between groups at baseline; DISCUS score at follow-up was increased in NC, SD, and 25D groups—greatest increase observed in 25D group; DISCUS score at follow- up decreased in the IN group; significant correlation observed between degree of dose reduction and DISCUS score (*r* = 0·51; *p* < 0·001); proportion with dyskinesia increased from 30% at baseline to 60% at follow-up in the 25D group, did not change in the SD or NC groups, and fell from 60 to 20% in the IN group.
Wigal et al. 1994 USA [41]	Prospective non randomised controlled design	n:43 (may include participants also reported in Wigal et al., 1993 [40]) mean age: 24 gender:67% male ethnicity: not reported ID = Severe/ Profound: 86%	Inpatient	**Intervention:** Medication review and dose reduction programme **Medication**: antipsychotics **Duration**: Variable **Length of follow up**:10 months	Rates of dyskinesia	63% of discontinuation group and 29% of dose reduction group developed dyskinesia. No dyskinesia reported in participants in no change, increase or unmedicated group.
Zuddas et al. 2000 Italy [42]	Prospective non randomised controlled design	n:10 mean age: 12.3 (7 to 17) gender: 70% male ethnicity: not reported ID = mild 2 Moderate 5 Severe 3	Community	**Intervention:** Following open label treatment with risperidone, three patients discontinued **Medication:** risperidone **Duration:** Medication tapered over 3 to 4 weeks **Length of follow up:** 5 months	CARS CPRS CGI C-GAS Kiddie SAD-PL Intellectual functioning was measured using the Raven progressive matrices. Physical and neurological examinations, vital signs and measurement of body weight were carried out for all patients at baseline, weekly for the first month and monthly thereafter.	3 participants discontinued risperidone, 6 months after withdrawal, atypical antipsychotic was represcribed. (risperidone × 2, olanzapine × 1) Patients who discontinued risperidone showed progressive behaviour deterioration.

Key: *AIMS* Abnormal Involuntary Movement Scale, *ABC* Aberrant Behavior Checklist, *ABS* Agitated Behaviour Scale, *BARNES* Barnes Akathisia Rating Scale, *BFCRS* Bush-Francis Catatonia Rating Scale, *BP* Blood Pressure, *CARS* Childhood Autism Rating Scale, *CGAS* Children’s Global Assessment Scale (CGAS), *CGI* Clinical Global Impressions, *CSM* Committee on Safety of Medicines, *CPRS* Comprehensive Psychopathological Rating Scale, *DAS* Disability Assessment Schedule, *DISCUS* Dyskinesia Identification System Condensed User Scale, *DISCO* Dyskinesia Identification System-Coldwater, *ECG* Electrocardiogram, *FBC* Full Blood Count, *HbA1c* Glycated Haemoglobin, *Kiddie SAD-PL* Kiddie Schedule for Affective Disorders and Schizophrenia, Present and Lifetyime Version LFTs: Liver Function Tests, *MOAS* Modified Overt Aggression Scale, *NOSIE* Nurses’ Observation Scale for Inpatient Evaluation, *PAS-ADD* Psychiatric Assessment Schedule for Adult with Developmental Disability, *PBS* Positive Behaviour Support, *PTH* Parathyroid Hormone, *RAND-36* measure of health related quality of life, *U + Es* Urea and elelctrolytes, *UPDRS* Unified Parkinson’s Disease Rating Scale, *SCOPA-AUT* Scales for Outcomes in Parkinson’s Disease - Autonomic Dysfunction

**Table 6:** Summary table of included pre post studies

Author/Year, Country	Study Design	Participants (n, age, gender, ethnicity, level of intellectual disabilities)	Setting	Intervention (including medication that was targeted, Duration of deprescribing intervention and length of follow up)	Outcome Measures	Summary of Findings
Brahm et al. 2003 USA [43]	Prospective Pre post design (no control)	n: 18 mean age: 42.7 (27 to 57) gender: 8% male ethnicity: not reported ID = moderate/severe/profoun d: 100%	Not reported	**Intervention:** Following warning of QTC with Thioridazine, 18 patients reviewed, antipsychotic medication reduced and QTc prolongation assessed **Medication:** Thioridazine, mesoridazine **Duration:** Variable **Length of follow up:** 8 weeks post discontinuation	ECG	15 participants discontinued thioridazine, increases in QTc prolongation times in five male patients after discontinuation of thioridazine, three patients slight increases and two patients more marked increases.
Branford D 1996 U.K. [44]	Retrospective Pre post design (no control)	n: 198 mean age: 43 (18 to 82) gender: 66% male ethnicity: not reported ID = borderline 1% mild 13% moderate: 30% severe: 56%	47% Inpatient 53% Community	**Intervention:** Medication review and dosage reduction programme **Medication:** thioridazine,chlorpromazine, zuclopentixol, haloperidol **Duration:** Mostly over 3 months **Length of follow up:** 12 months	Number reducing or discontinuing antipsychotic medication; challenging behaviour reports	123 patients underwent a reduction of antipsychotics. 16% of the total cohort of 198 were withdrawn from antipsychotics, 28% maintained on reduced dosage of antipsychotics. Out of the 123 undergoing reduction, 31 (25%) of 123 discontinued antipsychotic, 56 (46%) of 123 reduced dose, 27 (22%) of 123 same dose, and 9 (7%) of 123 increased dose; 31 (25%) of 123 no deterioration, 52 (42%) of 123 deterioration in behaviour, and 40 (33%) of 123 not reported
de Kuijper et al. 2018 The Netherlands [33]	Prospective Pre post design (no control)	n: 129 (includes an unspecified number of participants also reported in de Kuijper et al.*,* 2014 [23] and Ramerman et al., 2019 [32]) mean age: 49 (11.5–84.2) gender: 67% male ethnicity: not reported ID = mild 13% moderate 24% severe 44% profound 16% unspecified 3%	Community	**Intervention:** antipsychotic reduced over 14 weeks **Medication:** Not reported (see study [45]) **Duration**:14 weeks **Length of follow up**: 6 months following planned discontinuation	**Primary outcome measure:** Complete discontinuation at 16 weeks **Secondary outcome measures:** Complete discontinuation at 28 and 40 weeks, ABC, CGI-I, CGI-S	61% had completely discontinued antipsychotics at 16 weeks, 46% at 28 weeks, and 40% at 40 weeks. CGI-I: at 16 weeks 6% of participants had shown improvement and 9% worsening in behaviour; at 28 weeks, these percentages were 9 and 15%, and at 40 weeks 21 and 7%, respectively. At 28 weeks those who had not achieved complete discontinuation had significantly more often worsening in behaviour according to the CGI-I than those who had successfully discontinued.
de Kuijper et al. 2018 The Netherlands [45]	Prospective Pre post design (no control) **additional reporting of** [33]	(includes an unspecified number of participants also reported in de Kuijper et al.*,* 2014 [23] and Ramerman et al., 2019 [32])			**Primary outcome measure:** Complete discontinuation at 16 weeks **Secondary outcome measures:** Complete discontinuation at 28 and 40 weeks, ABC, Barnes, AIMS Number of times participants experienced new health problems Number of consultations by participants with their physician Number of new medication prescriptions or dosage changes Number of new nonpharmaceutical treatments. Number of changes in living circumstances and life events	61% had completely discontinued antipsychotics at 16 weeks, 46% at 28 weeks, and 40% at 40 weeks. ABC total scores increased in 49% of participants with unsuccessful discontinuation at 16 weeks Participants who achieved complete discontinuation had less-severe parkinsonism and lower incidence of health worsening during the study period compared with participants with incomplete discontinuation. A lower incidence of complete discontinuation was associated with higher ABC score, higher akathisia score and more frequent worsening of health.
Ellenor et al. 1977 USA [46]	Retrospective Pre post design (no control)	n: 208 mean age: not reported gender: not reported ethnicity: not reported ID = mild/moderate 20% Severe/profound 80%	Inpatient	**Intervention:** Pharmacist involvement in a behavioural review committee with aim of deprescribing psychotropic medicines over a two year programme **Medication:** anti anxiety/ antidepressants, antipsychotics, sedative/hypnotics, miscellaneous medication for behaviour management **Duration:** Variable **Length of follow up:** Variable	ABS (adaptive behaviour scales) Number of prescriptions and changes in dosages of medicines	ABS scores reported for 54 participants revealed a slight increase in adverse behaviours for all three groups; medication reduced, medication stopped and control group who had not been assessed by the behaviour review committee. Through discontinuance of medication a 50% reduction’ in the use of antianxiety-antidepressant agents, 17.5% reduction in antipsychotic agents, 57.6% reduction in sedativehypnotics and a 64.7% reduction in miscellaneous agents was reported. Of the total 183 drugs discontinued, 153 of these, or 83%, were discontinued without being replaced with a pharmacologically equivalent agent. In addition, of the 313 medications being administered to patients for behavior control at the completion of the two years, 124 of these, or 39.6%, were being administered at lower dosages Of the 313 drugs administered at the end of the study period, 87 medications were being administered at higher dosages or had been added to the patient’s drug regimen. Thus, while 39.6% were receiving lower dosages, 28% received. Higher dosages. The remaining 33% received the same dosage.
Ferguson et al. 1982 USA [47]	Prospective Pre post design (no control)	n: 250 mean age: not reported (adolescents and adults) gender: not reported ethnicity: not reported ID = not reported	Inpatient	**Intervention:** Introduction of interdisciplinary teams medication reviews with a goal to deprescribe antipsychotic medication typically by 25–50% per 30 day period **Medication:** antipsychotics **Duration:** Variable **Length of follow up:** Not reported	Number of individuals receiving neuroleptic drugs, mean daily drug dose, Number of individuals receiving dosage increases or decreases, number of individuals able to be maintained on lowered dosages or no drug at all	Data-based reviews resulted in decreased numbers of individuals receiving antipsychotic drugs, lower mean daily dosages, and less frequent dosage increases. 97% of the individuals receiving drug discontinuation or dosage decreases were not placed back on a drug or did not receive dosage increases
Fielding et al. 1980 USA [48]	Retrospective Pre post design (no control)	n: 192 mean age: 35 (SD 14.5) gender: 52% male ethnicity: not reported ID = severe/profound 86%	Inpatient	**Intervention:** Two phases Phase one: subjects participated in a 50-day assessment period consisting of 20 days during which they received their normal psychotropic medication followed by 30 days during which they received no medication. Medication was not tapered. At the end of the 30 days of non- medication, prescriptions were discontinued for those who did not show an increase in challenging behaviours. The 50-day assessment was repeated for individuals who remained on psychotropic medication. Phase two: 92 subjects who were unable to discontinue psychotropic were exposed to 30 days of 25% dose reduction which was repeated depending on adverse behaviours. Doses were also increased if necessary **Medication:** The most commonly prescribed medications were Mellaril and Thorazine. Other drugs used less often included Haldol, Trilafon, Quide, Navane, and Prolixin. **Duration:** 30 days for phase 1, Variable for phase 2 **Length of follow up:** nearly 2 years	Daily number of incidents of adverse behaviours Number of participants who discontinued or changed dose of psychotropic medicines	60% of participants who had been taking medications no longer needed them as no increase in frequency of episodes of behaviours that challenge. All but eight of the 68 residents whose medication gradually was reduced under phase two have achieved permanent dosage reductions while maintaining rates of maladaptive behavior comparable to those observed while medicated. While maladaptive behaviors increased slightly for some, they decreased or remained stable for the majority.
Findholt et al. 1990 USA [49]	Retrospective Pre post design (no control)	n: 208 mean age: not reported gender: not reported ethnicity: not reported ID = severe / profound: majority	Inpatient	**Intervention:** Behaviour and Medication review committee reviewed medication of participants at least every 6 months **Medication:** antipsychotics, antidepressants, anxiolytics **Duration:** Variable **Length of follow up:** Variable	Number of patients taking antidepressants, anxiolytics and antipsychotics and Number of patients receiving polypharmacy (defined in study as 2 or more psychotropic medicines) Cost savings based on medicine prices	April 1979 out of a total population of 590 persons, 208 (41%) were receiving antipsychotic medications, 69 (14%) were on antidepressants, and 67 (13%) were taking anxiolytics, with 52 residents on polypharmacy. March of 1987, with a total population of 436, these Numbers decreased to 52 (12%) on antipsychotics, 9 (2%) on antidepressants, 11 (3%) on anxiolytics, and 3 receiving polypharmacy. Cost savings for four most prescribed medicines $119.77 per day.
Gerrard 2020 U.K. [38]	Retrospective Pre post design (no control)	n: 66 (includes an unspecified number of participants also reported in Gerrard et al.*,* 2019 [37]) mean age: not reported gender: 50% male ethnicity: not reported ID = not reported	Community	**Intervention:** Pharmacist and PBS nurse reviewed patients with view to deprescribing in conjunction with views of patient, carers and families **Medication:** Included risperidone, olanzapine, quetiapine, amisulpride, aripiprazole,benzodiazepines **Duration:** Variable **Length of follow up:** Variable	Number of medicines stopped, Number of medicines restarted. For antipsychotic prescriptions- FBC, U + Es, LFT, TFT, Lipids, Glucose/HbA1c, prolactin, BP, weight, pulse and ECG	24 psychotropic medications were stopped; 20 of these were with PBS support. A further 22 people were undergoing the challenge which was not complete at end of study. Ten medications needed to be restarted post-discontinuation or increased post-reduction, with eight being in the unsupported clinic. On average, each person required a minimum of five reviews to fully undertake the challenge. The majority of medications stopped were antipsychotics,. Over half these prescriptions were for risperidone, which reflects the clinical practice that this antipsychotic was the preferred choice in behavioural intervention. Side effect burden reduced by 71% with a reduction of 50% of the starting dose or more. The main issues that improved were sedation, weight gain and postural hypotension.
Howerton et al. 2002 USA [50]	Prospective Pre post design (no control)	n: 159 mean age: not reported gender: 65% male ethnicity: not reported ID = mild 57 moderate 31 severe 39 profound 21 borderline 5 none 6	Community	**Intervention:** Evaluation of an interdisciplinary review team addressing polypharmacy **Medication**: Typical and atypical antipsychotics, anticonvulsants, SSRIs, antidepressants, lithium **Duration:** Variable **Length of follow up:** 3 months	Medicines stopped and started	Decrease in polypharmacy, discontinuation of unnecessary anticonvulsants. Thioridazine use was reduced by 63%, haloperidol by 72%, and chlorpromazine by 100%. Lithium was discontinued in 18 patients.
Inoue et al. 1982 Canada [51]	Retrospective Pre post design (no control)	n: 251 mean age: not reported gender: not reported ethnicity: not reported ID = borderline 2.5% mild 13.1% moderate: 33% severe:31.3% profound: 16.2% unspecified 3.6%	Inpatient	**Intervention:** Implementation of a pharmacy patient review service to address overprescribing of psychotropic medicines over 5 years. **Medication:** antipsychotics 72%, anxiolytics 16%, sedative/hypnotics-11%, antidepressants 9%, and others (e.g. lithium) 1% **Duration:** Variable **Length of follow up:** Variable	Number of psychotropic medicines discontinued Number of psychotropic medicines with dose changes	By the end of the five year period, 135 psychotropic medication orders for 121 patients were discontinued. The dosage reductions (25–75%; mean 48.6%) were made for 91 medication orders.
Janowsky et al. 2006 USA [52]	Retrospective Pre post design (no control)	n: 138 (may include participants also reported in Janowsky 2008 [53]) mean age: 48 (18 to 81) gender: 60% male ethnicity: not reported ID = severe to profound 100%	Inpatient	**Intervention**: Medication review and dosage reduction programme **Medication**: Typical and atypical antipsychotics **Duration**: Not reported **Length of follow up**: 10 years	Number discontinuing antipsychotic medication	55% Successfully discontinued antipsychotic medication 36% relapsed on withdrawal requiring dose increases or represcribing.
Janowsky 2008 USA [53]	Retrospective Pre post design (no control)	n: 57 (may include participants also reported in Janowsky 2006 [52]) mean age: 52 (30 to 78) gender: 65% male ethnicity: not reported ID = severe to profound 100%	Inpatient	**Intervention:** Medication review and dosage reduction programme **Medication:** haloperidol (*n* = 24), thioridazine (*n* = 20), chlorpromazine (*n* = 7), thiothixine (*n* = 5), and loxapine (*n* = 1) **Duration:** Not reported **Length of follow up:** Up to 15 years	Number discontinuing antipsychotic medication Number of episodes of challenging behaviour	4 (8%) of 49 discontinued antipsychotic medication and 45 (92%) of 49 could not discontinue antipsychotic medication; 2 (4%) of 49 no deterioration in behaviour and 47 (96%) of 49 experienced behavioural relapse.
Jauernig et al. 1995 Australia [54]	Retrospective Pre post design (no control)	n: 25 mean age: not reported gender: not reported ethnicity: not reported ID = not reported	Inpatient	**Intervention:** Medication review and dosage reduction programme involving maximum monthly dose reduction of 25% **Medication:** thioridazine,chlorpromazine, haloperidol, fluphenazine, trifluoperazine **Duration:** Variable **Length of follow up:** 2 years	Number reducing or discontinuing antipsychotic medication; Number of episodes of challenging behaviour	3 (12%) discontinued antipsychotic medication, 19 (76%) underwent dose reduction, and 3 (12%) no change in dose; challenging behaviour frequency at follow-up lower than in baseline in all 3 patients (100%) whose antipsychotic had been discontinued and in 15 patients (79%) of 19 who underwent dose reduction.
LaMendola et al. 1980 USA [55]	Retrospectives Pre post design (no control)	n: not reported mean age: not reported gender: not reported ethnicity: not reported ID = not reported	Inpatient	**Intervention:** Medication review and dosage reduction programme over 4 years **Medication:** included antipsychotics and benzodiazepines **Duration:** not reported **Length of follow up:** not reported	Percentage of patients prescribed psychotropic medicines, percentage of patients prescribed major and minor tranquilisers.	Patients prescribed psychotropic medication decreased from 34 to 21%, percentage prescribed major tranquillisers fell from 27 to 20% and minor tranquilisers were no longer used having accounted for 5% of patients.
Lindsay et al. 2004 USA [56]	Prospective Pre post design (no control)	n: 14 mean age: 9.7 (5–13) gender: 93% male ethnicity: not reported ID = 100% borderline to moderate	Community	**Intervention:** After a mean exposure of 8.9 months because of excessive weight gain, or excessive appetite, or insufficient clinical response, antipsychotic medication stopped **Medication:** risperidone **Duration:** sudden discontinuation **Length of follow up:** 24 months	Body weight	Standardised weight at 12 and 24 months after discontinuation of risperidone was not distinguishable from standardized weight before risperidone was initially prescribed
Luchins et al. 2004 USA [57]	Retrospective Pre post design (no control)	n: 95 mean age: 32 (18 to 73) gender: 60% male ethnicity: not reported ID = mild / moderate: 70 severe / profound: 25	Inpatient	**Intervention**: Interdisciplinary team programme to review psychotropic medication with a view to reduce or discontinue **Medication**: antipsychotics **Duration**: Variable **Length of follow up**: Variable	Dosage changes of antipsychotics and other psychotropic medicines. Unvalidated behaviour rating tool	Reduction of antipsychotics associated with improvement in behaviour. 41 participants were receiving an alternative psychotropic medicine at the end of the study period, with 5 of them receiving two such drugs concurrently. The alternative drugs used were as follows: lithium (*n* = 26) carbamazepine *(n =* 9) buspirone *(n =* 9), and propranolol (n = 2). The prescribing of these other psychotropic medicines were associated with a reduction in the prescribing of antipsychotic medicines,
Marholin et al. 1979 USA [58]	Prospective Pre post design (no control)	n: 6 mean age: 35 (27 to 53) gender: 100% male ethnicity: not reported ID = severe 100%	Inpatient	**Intervention:** antipsychotics were withdrawn and readministered using a double-blind B-A-B (drug-placebo-drug) design. **Medication:** phenothiazine antipsychotics **Duration:** Sudden discontinuation for 23 days **Length of follow up:** 48 days	Observations on the ward and during workshop tasks	Findings highly individualised and mixed When chlorpromazine was withdrawn and reinstated, reversible changes occurred in at least one category of behavior for all subjects.
Matthews et al. 2003 U.K. [59]	Retrospective Pre post design (no control)	n: 77 mean age: 45.5 (16 to 81) gender: 51%male ethnicity: not reported ID = mild 22% moderate: 22% severe / profound: 39% unspecified: 17%	Community	**Intervention:** Retrospective case note analysis to observe effects of discontinuation **Medication:** thioridazine **Duration:** not reported **Length of follow up:** Not reported	Significant adverse events on /following Thioridazine withdrawal	Over 50% of those on regular thioridazine experienced adverse events during or following drug withdrawal. Adverse events were significantly associated with the duration of previous thioridazine prescription. Problems encountered included reemergence of psychosis or mood disturbance, escalation of arousal, aggression, anxiety, self-injury, sexual disinhibition, and ritualised behaviours. Further details of adverse effects not reported.
Marcoux 1985 USA [60]	Prospective Pre post design (no control)	n: not reported mean age: not reported gender: not reported ethnicity: not reported ID = not reported	Inpatient	**Intervention:** Interdisciplinary team programme to review psychotropic medication with a view to reduce or discontinue **Medication:** chlorpromazine, piperidine, mesoridazine, thioridazine, piperazine,fluphenazine, perphenazine, prochlorperazine, tripfluoperazine, haloperidol, thiothixene, molindone **Duration:** Not reported **Length of follow up:** Not reported	antipsychotic dosages	antipsychotic dosages decreased at a projected annual rate of 17% and no significant withdrawal reactions reported. This dosage decrease has saved the Institution approximately $2800 to $3200 in medication costs after a 10-month period.
May et al. 1995 USA [61]	Prospective Pre post design (no control)	n: 23 mean age: 42 (24–62) gender: 100% male ethnicity: not reported ID = severe/ profound: 100%	Inpatient	**Intervention:** antipsychotic dose reduced by 10% every 3 months until discontinued **Medication:** risperidone **Duration:** Variable **Length of follow up:**3–4 years	Number of incidents of challenging behaviour	Three groups to describe changes in challenging behaviour: Transient worsening (n = 9; 39% Progressive improvement (*n* = 5; 22%) Persistent worsening (n = 9; 39%).
Newell et al. 2000 USA [62]	Prospective Pre post design (no control)	n:6 (may include participants also reported in Newell et al.*,* 2001 [63] and Newell et al.*,* 2002 [64]) mean age: 36.8 (14 to 50) gender:67% male ethnicity: not reported ID = mild moderate 4 severe 1 profound 1	Inpatient	**Intervention:** antipsychotic dose reduced by 25% every 3 months until discontinued **Medication**: haloperidol, thioridazine, mesoridazine **Duration:** Variable **Length of follow up:** 6 months to 2 years post discontinuation	Video analysis of lip movement DISCUS	Dyskinetic movements increased during antipsychotic withdrawal followed by a reduction post-discontinuation.
Newell et al. 2001 USA [63]	s	n:26 (may include participants also reported in Newell et al.*,* 2000 [62] and Newell et al.*,* 2002 [64]) mean age: 34.9 (18 to 52) gender:69% male ethnicity: not reported ID = mild 1 moderate 5 severe 12 profound 8	Inpatient	**Intervention:** antipsychotic dose reduced by 25% every 2 to 4 months until discontinued **Medication:** haloperidol, thioridazine, chlorpromazine, mesoridazine, lozapine, trifluperazine **Duration:** Variable **Length of follow up**: 12 months post discontinuation	DISCUS	Mean total DISCUS increased significantly during antipsychotic withdrawal, returning to baseline. Prevalence: baseline 31% during withdrawal 85% follow up 38%.
Newell et al. 2002 USA [64]	Prospective Pre post design (no control)	n:20 (may include participants also reported in Newell et al.*,* 2000 [62] and Newell et al., 2001 [63]) mean age: 36.6 (SD 8.6) gender:75% male ethnicity: not reported ID = severe / profound 100%	Inpatient	**Intervention:** antipsychotic dose reduced by 25% every 3 months until discontinued **Medication:** haloperidol, thioridazine, chlorpromazine, lozapine, trifluperazine **Duration:** Variable **Length of follow up**: 12 months post discontinuation	Postural stability DISCUS	Postural stability changed significantly during course of medication withdrawal and tended to return to baseline values at follow-up; mean total DISCUS increased significantly from baseline during antipsychotic withdrawal before returning to baseline values at follow up.
Ramerman et al. 2019 The Netherlands [34]	Prospective Pre post design (no control)	n: 128 (includes participants also reported in de Kujper et al., 2014 [33] and Ramerman et al., 2019 [32]) mean age: 48 (10–68) gender: 71% male ethnicity: not reported ID = mild 15.6% moderate 21.9% severe 46.9% profound 15.6%	Community	**Intervention:** Antipsychotic reduced over 14 weeks, 12.5% of the baseline dosage every two weeks data combined from two studies and part of clinical care. **Medication:** risperidone 23.4% olanzapine 8.5%, quetiapine 1.6%, clozapine 2.3%, aripiprazole 0.8%, pipamperone 34%, haloperidol 5.4%, pericyazine 4%, zuclopentixol 5.5%, levomepromazine 2.3%, pimozide 5.5% **Duration:** 14 weeks **Length of follow up:** 6 months following planned discontinuation	Primary outcome measure: healthrelated quality of life RAND-36 Secondary outcome measures: ABC, UPDRS, SCOPAAUT	Physical well-being showed an increase in the group that had achieved complete discontinuation. Social functioning showed a decrease in the group that incompletely discontinued, which recovered at follow-up. Mental wellbeing decreased at 16 weeks, but recovered at follow up, regardless of complete or incomplete discontinuation.
Shankar et al. 2019 U.K. [65]	Retrospective Pre post design (no control)	n: 71 mean age: not reported gender: not reported ethnicity: not reported ID = not reported	Community	**Intervention**: Usually dose changes were 10–25% of baseline dose reduced every 6–8 weeks **Medication:** antipsychotics **Duration:** Variable **Length of follow up:** 3 months	Number reducing or discontinuing antipsychotic medication Number of patients requiring hospital admission or change in placement	46.5% (33/71) discontinued antipsychotic medication 11.3% (8/71) reduced over 50% of antipsychotic dosage At three months follow-up no one required hospital admission or change in placement.
Spreat et al. 1993 USA [66]	Pre post design (no control)	n:86 mean age: not reported gender: not reported ethnicity: not reported ID = not reported	Inpatient	**Intervention:** Medication reduction trials **Medication:** haloperidol,mesoridazine, thioridazine,thioxanthene, chlorpromazine,thrifluoperazine,mo lindone,fluphenazine, chlorpromthixene **Duration**: Variable **Length of follow up**:12 months post discontinuation	Changes in antipsychotic prescribing	> 50% dose reduction or discontinuation: 14 (16%) ≤50% dose reduction: 26 (30%) No change or increased dose: 46 (53%).
Stevenson et al. 2004 U.K. [67]	Retrospective Pre post design (no control)	n: 119 mean age:44 (18–72) gender: 58% male ethnicity: Not reported ID = mild 27.7% Moderate: 21.8% Severe: 18 (15.1% Profound:7.6% Not reported: 27.7%	Community	**Intervention**: Medication withdrawal programme following CSM advice. **Medication:** Thioridazine **Duration:** Variable **Length of follow up:** Variable	Number of people withdrawn from antipsychotic Number of new prescriptions Numbers needing extra carer support, Numbers of placement breakdown, family problems, admissions to hospital	7.6% completely withdrew from antipsychotic medicines, and 48.7% experienced onset/deterioration in problem behaviours or mental illhealth. The cost to the intellectual disabilities psychiatric service (over and above that of routine psychiatric care) was £258,050. 10 people required increased levels of carer support to be provided; seven were excluded from a day centre placement, one person experienced a placement breakdown and moved to a new home, and six experienced considerable family problems. 14 hospital admissions to an intellectual disabilities psychiatric assessment and treatment unit

Key: *AIMS* Abnormal Involuntary Movement Scale, *ABC* Aberrant Behavior Checklist, *ABS* Agitated Behaviour Scale, *BARNES* Barnes Akathisia Rating Scale, *BFCRS* Bush-Francis Catatonia Rating Scale, *BP* Blood Pressure, *CARS* Childhood Autism Rating Scale, *CGAS* Children’s Global Assessment Scale (CGAS), *CGI* Clinical Global Impressions, *CSM* Committee on Safety of Medicines, *CPRS* Comprehensive Psychopathological Rating Scale, *DAS* Disability Assessment Schedule, *DISCUS* Dyskinesia Identification System Condensed User Scale, *DISCO* Dyskinesia Identification System-Coldwater, *ECG* Electrocardiogram, *FBC* Full Blood Count, *HbA1c* Glycated Haemoglobin, *Kiddie SAD-PL* Kiddie Schedule for Affective Disorders and Schizophrenia, Present and Lifetyime Version LFTs: Liver Function Tests, *MOAS* Modified Overt Aggression Scale, *NOSIE* Nurses’ Observation Scale for Inpatient Evaluation, *PAS-ADD* Psychiatric Assessment Schedule for Adult with Developmental Disability, *PBS* Positive Behaviour Support, *PTH* Parathyroid Hormone, *RAND-36* measure of health related quality of life, *U + Es* Urea and elelctrolytes, *UPDRS* Unified Parkinson’s Disease Rating Scale, *SCOPA-AUT* Scales for Outcomes in Parkinson’s Disease - Autonomic Dysfunction

**Table 7:** Summary table of included case studies

Adams et al. 2017 U.K. [68]	Case study	n: 1 age: 30 gender: male ethnicity: not reported ID = mild	Community	**Intervention: **Two psychotropic medicines and a beta blocker were deprescribed separately **Medication:** olanzapine, carbamazepine, propranolol **Duration:** 2 years **Length of follow up:** ongoing; 2 years from when deprescribing process began	Discontinuation or reduction of dose of psychotropic medicines General wellbeing Weight	Olanzapine and carbamazepine were stopped Propranolol was reduced and deprescribing process ongoing. He is less tired, more alert, and better able to express himself. He has expanded his activities and increased his access to the community. He can cope better with changes to his routine. His behaviours are well managed by the behavioural strategies in place, and he has now been discharged by the psychiatrist to the GP. Weight reduced form 82 kg to 56 kg which was within recommended BMI
Bastiampillai et al. 2014 Australia [69]	Case study	n: 1 age: 28 gender: male ethnicity: not reported ID = moderate	Inpatient	**Intervention:** Following warning from CSM in UK, thioridazine withdrawn and patient changed to risperidone. **Medication:** risperidone **Duration:** not reported **Length of follow up:** not reported	Behaviour and mental health symptomatology	Delusions and hallucinations reported within 2 weeks of stopping Thioridazine, hospitalised for 2 ½ years, unresponsive to several other Antipsychotics, prescribed clozapine and went into remission
Brahm et al. 2009 USA [70]	Case study	n: 1 age: 53 gender: male ethnicity: white ID = moderate	Inpatient	**Intervention:** deprescribing **Medication:** ziprasidone **Duration:** Not reported **Length of follow up:** Not reported	Episodes of inappropriate sexual behaviour	Episodes of inappropriate sexual behaviour increased from 2 to 3 per month prior to discontinuation to 21 episodes the following month post discontinuation
Branford D 2019 U.K. [14]	Case studies × 3	1. n: 1 age: 35 gender: male ethnicity: not reported ID = not reported 2. n: 1 age: not reported gender: not reported ethnicity: not reported ID = not reported 3. n: 1 age: not reported gender: male ethnicity: not reported ID = not reported	1. Community 2. Community 3. Community		1. Successful discontinuation, quality of life observations 2. Successful discontinuation 3. Successful discontinuation, quality of life observations	1. Antipsychotic discontinued post discontinuation he was more lively, wanting to go on more outings and tackle new activities. Staff aware to offer active support to meet his needs and his grabbing behaviours are understood. 2. Chlorpromazine discontinued 3. Antipsychotic discontinued Patient is now reported to be very positive. He enjoys walks, his self-confidence has gone up and his life is changing. He is cooking for himself and is keen to find work.
Connor 1998 USA [71]	Case study	n: 1 age: 11 gender: not reported ethnicity: not reported ID = moderate	Community	**Intervention:** deprescribing **Medication:** thioridazine **Duration:** 3 weeks **Length of follow up:** 12 weeks	AIMS	Within 1 week of discontinuation patient developed new onset multiple involuntary movements consisting of jaw grinding, oral dyskinesias, bilateral hand rolling, vermiform tongue movements. and bilateral choreiform movements of his digits. When methylphenidate that was being co prescribed was also discontinued the movement disorder resolved.
Dillon 1990 USA [72]	Case study	n: 1 age: 7 yrs. 11 months gender: male ethnicity: not reported ID = borderline	Community	**Intervention:** deprescribing **Medication:** clonidine **Duration:** 4 weeks **Length of follow up:** not reported	Adverse behaviours	When withdrawn from clonidine over 4 weeks multiple self-destructive behaviours involving the theme of suffocation were reported
Faisal et al. 2021 Ireland [73]	Case study	n:1 age:13 gender: female ethnicity: not reported ID = moderate	Community	**Intervention:** deprescribing **Medication:** risperidone **Duration:** unclear **Length of follow up:** unclear	Overall clinical presentation, BFCRS	In first week following risperidone discontinuation nursing staff observed gradual change in behaviour, insomnia, increased salivation, mutism, echopraxia, immobility. Catatonic symptoms occurred over 8 weeks following discontinuation followed by admission to paediatric high dependency unit. Responded to im lorazepam, Resolution of catatonic symptoms after 7 weeks in hospital
Ghaziuddin et al. 1990 USA [74]	Case study	n: 1 age: 34 gender: female ethnicity: not reported ID = moderate	Inpatient	**Intervention:** deprescribing **Medication:** diazepam **Duration:** 6 weeks **Length of follow up:** 6 months	Challenging behaviour mental health symptomatology dose of medication	10 days after discontinuation of diazepam resembling mania reported. Improvement noted when diazepam represcribed.
Lee et al. 2019 U.K. [75]	Case study	n: 1 age: early 40s gender: female ethnicity: not reported ID = moderate	Community	**Intervention:** Flexible medication reduction in collaboration with PBS framework Involving an initial 25% reduction with further changes dictated by behavioural data, the impact of any side effects, the opinions of care staff and of family members **Medication**: risperidone **Duration**: 6 months **Length of follow up:** not reported	Dose of medication challenging behaviour	Reduction slowed down in response to increase in grabbing behaviours. Risperidone stopped PBS supported medication reduction reduced challenging behaviour
McLennan 2019 Canada [76]	Case study	n: 1 age: 15 gender: male ethnicity: white ID = moderate	Community	**Intervention:** deprescribing 6 psychotropic medicines, **Medication:** quetiapine, lamotrigine, clonidine, olanzapine, sertraline, and ziprasidone **Duration:** whole process over approx.18 months **Length of follow up:** not reported	Number of medicines stopped	Quetiapine, lamotrigine, clonidine, olanzapine, sertraline successfully discontinued ziprasidone added trazadone prn added for sleep ziprasidone associated with unsuccessful attempt to deprescribe

Key: ^a^ Possibility of potential overlap of participants with other included studies by the same author(s)

*AIMS* Abnormal Involuntary Movement Scale, *ABC* Aberrant Behavior Checklist, *ABS* Agitated Behaviour Scale, *BARNES* Barnes Akathisia Rating Scale, *BFCRS* Bush-Francis Catatonia Rating Scale, *BP* Blood Pressure, *CARS* Childhood Autism Rating Scale CGAS: Children’s Global Assessment Scale (CGAS) CGI: Clinical Global Impressions, *CSM* Committee on Safety of Medicines, *CPRS* Comprehensive Psychopathological Rating Scale, *DAS* Disability Assessment Schedule, *DISCUS* Dyskinesia Identification System Condensed User Scale, *DISCO* Dyskinesia Identification System-Coldwater, *ECG* Electrocardiogram, *FBC* Full Blood Count, *HbA1c* Glycated Haemoglobin, *Kiddie SAD-PL* Kiddie Schedule for Affective Disorders and Schizophrenia, Present and Lifetyime Version LFTs: Liver Function Tests, *MOAS* Modified Overt Aggression Scale, *NOSIE* Nurses’ Observation Scale for Inpatient Evaluation, *PAS-ADD* Psychiatric Assessment Schedule for Adult with Developmental Disability, *PBS* Positive Behaviour Support, *PTH* Parathyroid Hormone, *RAND-36* measure of health related quality of life, *U + Es* Urea and elelctrolytes, *UPDRS* Unified Parkinson’s Disease Rating Scale, *SCOPA-AUT* Scales for Outcomes in Parkinson’s Disease - Autonomic Dysfunction

**Table 8:** Summary of quality appraisal of included studies

	Author/Year	Key Sources of Bias
**Summary of quality assessment for RCTs**		
**1**	Research Units on Pediatric Psychopharmacology Autism Network. 2005 [20]	• parent raters • process for randomisation, recruitment and sampling unclear • short follow up period
**2**	Ahmed et al. 2000 [21]	• selection bias, not blinded, no allocation concealment, process for randomisation, recruitment and sampling unclear • no reporting of PRN prescribing and administration, non-pharmacological interventions, level of support, co morbidities, level of ID • baseline characteristics of experimental and control groups uneven • short follow up period
**3**	de Kuijper et al. 2014 [23]	• selection bias, not blinded, no allocation concealment, process for randomisation, recruitment and sampling insufficient. • no reporting of other psychotropic medication prescribing, PRN prescribing and administration, non-pharmacological interventions, level of support, co morbidities • short follow up period
**4**	de Kuijper, G., et al. 2013 [24]	• side arm of previous study • outcomes are statistically significant but unclear if clinically significant. • lack of evaluation of confounding factors e.g. changes in diet and exercise • measurements and results were not AP specifically reported
**5**	de Kuijper et al. 2014 [25]	• side arm of previous study • confounding factors that could have affected the results include linking effects to the actual AP eg risperidone has greater effect on prolactin than others in the sample, olanzapine has a greater effect on weight gain. • measurements and results were not AP specifically reported
**6**	Haessler et al. 2007 [28]	• recruitment, randomisation and blinding process unclear • no power calculation • baseline comparability unclear • short follow up period • unclear if outcomes were discontinuation effects or reduced effects of placebo • no tapering • no reporting of other psychotropic medication prescribing, PRN prescribing and administration, non-pharmacological interventions, level of support, co morbidities
**7**	Hassler et al. 2011 [29]	• see no 6 Haessler, F., et al. 2007 • small sample • not blinded
**8**	Heistad et al. 1982 [30]	• no power calculation • rating scales not specified • rate of discontinuation unclear • process of randomisation unclear • simultaneous withdrawal of antiparkinsonian medication, • no reporting of PRN prescribing and administration, non-pharmacological interventions, level of support, co morbidities • short follow up period
**9**	McNamara et al. 2017 [31]	• significantly underpowered • trial finished prematurely and reported as pilot
**10**	Ramerman et al. 2019 [32]	• no allocation concealment, • no power calculation • no reporting of other psychotropic medication prescribing, PRN prescribing and administration, non-pharmacological interventions, level of support, co morbidities
**11**	Smith et al. 2002 [22]	• See no 2 Ahmed, Z., et al. 2000
**Summary of quality assessment for non randomised controlled trials (CTs)**		
**1**	Aman et al. 1985 [35]	• subjective outcome measurements • sampling and recruitment process unclear • no power calculation • short follow up period
**2**	Carpenter et al. 1990 [36]	• selection method unclear • exposure inadequately ascertained • causality inadequately ascertained • short follow up period
**3**	Gerrard et al. 2019 [37]	• recruitment and allocation process unclear • length of follow up not reported.
**4**	Swanson et al. 1996 [39]	• selection bias • control group inadequately matched • inadequate blinding • statistics or statistical tests inadequately reported or inappropriate • institutional setting • intervention poorly defined
**5**	Wigal et al. 1993 [40]	• selection bias • statistics or statistical tests inadequately reported or inappropriate • missing baseline information • intervention poorly defined
**6**	Wigal et al. 1994 [41]	• selection bias • statistics or statistical tests inadequately reported or inappropriate • missing baseline information • intervention poorly defined
**7**	Zuddas et al. 2000 [42]	• no power calculation and small number of participants • sampling and recruitment unclear • confounding factors include psychological, behavioural and environmental interventions
**Summary of quality assessment for non randomised no control Pre Post Studies (PPSs)**		
**1**	Brahm et al. 2003 [43]	• missing baseline information • variable deprescribing schedules
**2**	Branford 1996 [44]	• patients living with relatives, those in unsupervised accommodation, and those in accommodation where staff were unwilling to engage excluded from study • selection bias • use of unvalidated measures or non-standard assessment tools • missing baseline information • intervention poorly defined • selective reporting or incomplete
**3**	de Kuijper et al. 2018 [33]	• sampling and recruitment unclear • rater reliability
**4**	de Kuijper et al. 2018 [33]	• as above
**5**	Ellenor et al. 1977 [46]	• intervention poorly defined • outcomes measures unclear
**6**	Ferguson et al. 1982 [47]	• duration of intervention variable • length of follow up not reported
**7**	Fielding et al. 1980 [48]	• unvalidated outcome measures
**8**	Findholt et al. 1990 [49]	• high turnover of medical staff delivering the intervention
**9**	Gerrard 2020 [38]	• author / researcher is the clinician delivering the intervention
**10**	Howerton et al. 2002 [50]	• differing referral rates from the various primary care providers • poor follow up rates
**11**	Inoue et al. 1982 [51]	• limited baseline information
**12**	Janowsky et al. 2006 [52]	• selection bias • missing baseline information • intervention poorly defined
**13**	Janowsky et al. 2008 [53]	• selection bias • missing baseline information • intervention poorly defined
**14**	Jauernig et al. 1995 [54]	• selection bias • use of unvalidated measures or non-standard assessment tools • missing baseline information • intervention poorly defined
**15**	LaMendola et al. 1980 [55]	• intervention poorly defined • duration of intervention and length of follow up not reported • missing baseline and outcomes information
**16**	Lindsay et al. 2004 [56]	• poorly defined methodology • small sample size • inconsistent weighing scales • no BMI measurements • missing data • no reporting of dietary modification, environmental and behavioural interventions
**17**	Luchins et al. 2004 [57]	• poor reporting of duration of intervention and length of follow up
**18**	Marcoux 1985 [60]	• intervention poorly defined
**19**	Marholin et al. 1979 [58]	• selection method unclear • causality not adequately ascertained • short follow up
**20**	Matthews et al. 2003 [59]	• duration of intervention and length of follow up missing • outcomes poorly reported
**21**	May et al. 1995 [61]	• small sample size • selection bias • use of unvalidated measures or non-standard assessment tools • statistics or statistical tests inadequately reported or inappropriate • missing baseline information • selective reporting or incomplete outcome data
**22**	Newell et al. 2000 [62]	• small sample size • selection bias • use of unvalidated tools • missing baseline and outcome data
**23**	Newell et al. 2001 [63]	• selection bias
**24**	Newell et al. 2002 [64]	• selection bias • inadequate blinding • use of unvalidated measures or non-standard assessment tools • statistics or statistical tests inadequately reported or inappropriate • missing baseline information
**25**	Ramerman et al. 2019 [32]	• weak methodology of combining studies with different designs
**26**	Shankar et al. 2019 [65]	• unvalidated outcome tools
**27**	Spreat et al. 1993 [66]	• selection bias • institutional setting • missing baseline information • intervention poorly defined
**28**	Stevenson et al. 2004 [67]	• weak methodology • use of non standardised assessment tools • subjective outcome measurements
**Summary of quality assessment of case studies**		
**1**	Adams and Sawhney 2017 [68]	• selection method unclear
**2**	Bastiampillai et al. 2014 [69]	–
**3**	Brahm et al. 2009 [70]	–
**4**	Branford 2019 [14]	• selection method unclear
**5**	Connor D 1998 [71]	
**6**	Dillon J 1990 [72]	• outcome and causality inadequately ascertained
**7**	Faisal et al. [73]	•
**8**	Ghaziuddin et al. 1990 [74]	–
**9**	Lee et al. 2019 [75]	• selection method unclear
**10**	McLennan J 2019 [76]	–

Included studies were carried out in nine countries, in both inpatient (*n* = 31) and community settings (*n* = 24). One study did not report setting.

Details of participant characteristics and numbers were incompletely reported in several studies, and in studies conducted by the same researchers, there was lack of clarity regarding duplication of participants [34, 37, 77].The total number of participants across all studies where reported was 3292. The percentage of participants reported to have severe/profound intellectual disabilities varied across study types ranging from 49% for RCTs, 62% for non-randomised controlled studies and 72% for pre post studies without randomisation control. One case study reported the participant to have severe/profound intellectual disabilities. Furthermore, the level of intellectual disability was incompletely or not reported in 33% of studies and the amount and type of support provided to participants was not reported in any of the studies. Ethnicity was reported in only five studies.

The most frequently deprescribed psychotropic medicines across all studies were typical and atypical antipsychotics. Aside from one RCT, the prescribing and administration of pro re nata (PRN) medication for the management of behaviours that challenge was incompletely reported [31].

Intervention approaches ranged from sudden discontinuation to gradually tapering dosage over 28 weeks. Sixteen studies reported the deprescribing intervention as integral to or supported by the wider multidisciplinary team [37, 38, 44, 46–51, 54, 55, 57, 65, 66, 75]. However, there were no data reported regarding working across organisation boundaries such as between primary and secondary care and no data reporting specific non pharmacological interventions to support deprescribing although for three studies the deprescribing interventions were in the context of a Positive Behaviour Support (PBS) framework [37, 38, 75]. Evidence of pharmacists working within the multidisciplinary team (MDT) was reported in 11 studies [37, 38, 44, 47, 49, 51, 54, 57, 60, 65, 75] and pharmacist non-medical prescribers delivering the interventions were reported in three studies, although the same pharmacist prescriber was involved in all three [37, 38, 75]. Follow up ranged from immediately after medication was reduced or discontinued to 15 years. For 22 studies, follow up was variable or not specified. Outcomes were measured using a range of standardised rating tools and questionnaires. Input from patients, carers, and family and models of co-production in developing multidisciplinary deprescribing interventions were not reported. The reporting of shared decision-making approaches involving patients, carers, and clinicians within deprescribing interventions were reported in three studies (all within a Positive Behavioural Support (PBS) framework) [37, 38, 75, 78]. Quality of Life outcomes were only reported in one pre post study [34] and one paper reporting three case studies [14].

Across all study types there was incomplete reporting of rates of complete psychotropic discontinuation, at least 50% psychotropic dose reduction, represcribing, behavioural changes and emergence of adverse effects. Where reported in RCTs using a tapering approach to deprescribing, rates of complete deprescribing ranged from 33 to 84% [20, 21, 23, 31, 32]. Relapse rates due to worsening of behaviour ranged from 62.5% to no worsening. In the non randomised group of studies, Gerrard et al. [37] reported up to 60% complete discontinuation with a further 50% achieving a 50% dose reduction with only one person requiring represcribing This contrasted to findings by Zuddas et al. [42] who reported that all three people who achieved discontinuation displayed behavioural deterioration requiring represcribing.

### Randomised control trials (RCTs)

Seven RCTs evaluated the effects of deprescribing antipsychotic medicines [20, 21, 23, 30–32, 79], three on typical antipsychotics, three on atypical antipsychotics, and one on both types. Four studies were conducted in community settings [20, 23, 31, 32], two studies were carried out in an inpatient settings [30, 79] and one study included a mix of both [21]. Sample sizes ranged from 22 to 100 participants, with participant ages, where reported, ranging from 5 years to 78 years, with all 7 studies reporting outcomes for adults, 4 studies reporting outcomes for adolescents (ages 10--19 years [80]) and two studies reporting outcomes for children. The majority of participants were male ranging from 48 to 87% across RCTs. Length of follow up period varied from 4 weeks to 9 months following discontinuation or maximum dosage reduction. Primary outcome measures were firstly the changes in frequency and intensity of episodes of behaviours that challenge at follow up (we report follow up as time after planned complete discontinuation or maximum dosage reduction) and secondly, numbers of participants who reduced or stopped their antipsychotic medication.

#### Changes in behaviours that challenge

Assessment of the effects of deprescribing antipsychotics on behaviours that challenge was a primary outcome in all seven RCTs. Deprescribing antipsychotic medication was associated with a reduction in behaviours that challenge irrespective of whether the antipsychotic was tapered over 14 or 28 weeks in an RCT by de Kuijper et al. [23] This study involving 98 participants in community settings reported firstly that higher ratings of extrapyramidal and autonomic symptoms at baseline were associated with less improvement of behavioural symptoms after discontinuation; and secondly, higher baseline Aberrant Behavior Checklist (ABC) scores were associated with an increased likelihood of incomplete discontinuation [23].

Authors of studies where antipsychotic doses were reduced over 6 months [31] or 4 months [21] reported no clinically important changes in participants’ levels of aggression or behaviours that challenge at 9 months and 1 month respectively after planned discontinuation. Furthermore in a study by Ramerman et al. [32] study no change in irritability was reported when risperidone was reduced over 14 weeks in 86 participants compared to placebo.

In a study of the effects of withdrawal of zuclopentixol by Hassler et al. [79] behaviours that challenge increased at 12 weeks after sudden discontinuation of zuclopenthixol in 20 participants compared to the 19 participants that continued to be prescribed the antipsychotic. Heistad et al. [30] also reported increases in behaviours that challenge in participants undergoing deprescribing of thioridazine in a series of 5 separate groups within an RCT. Rates of relapse of behaviours that challenge were reported to be higher at 5 weeks follow up when risperidone was discontinued over 3 weeks in 38 adolescents and children in an RCT by the Pediatric Psychopharmacology Autism Network research units [20]. Relapse rates were 62.5% for gradual placebo substitution and 12.5% for continued risperidone [20].

The deprescribing interventions of the three RCTs reporting overall increase in behaviours that challenge involved sudden discontinuation [30, 79] or tapering over the short time of 3 weeks. This compares to antipsychotics deprescribed over 14 to 28 weeks in studies reporting no change or a reduction in behaviours that challenge [21, 23, 31, 32]. In addition the follow up periods in the three RCTs [20, 30, 79] reporting increases in behaviours that challenge were shorter; 4 to 12 weeks compared to 4 weeks to 12 months in studies reporting no change or a reduction in behaviours that challenge [21, 23, 31, 32]. Two [30, 79] of the three RCTs reporting increases in behaviours that challenge were conducted in inpatient settings. Three [23, 31, 32] of the four studies reporting more favourable results regarding behaviours that challenge, were carried out in community settings, the fourth study [21] involving participants in both community and inpatient settings. The studies reporting less favourable effects on behaviours that challenges involved larger percentages of participants with severe or profound intellectual disabilities ranging from 34 to 72% [20, 30, 79]compared to 24 to 63% [23, 32] in two of the four studies reporting more favourable outcomes although two studies did not report level of intellectual disability [21, 31].

#### Reduction /discontinuation completion outcomes

Four studies [21, 23, 31, 32] used a study design involving tapering of the dose of antipsychotic dose three [21, 23, 32] of which reported numbers of participants achieving complete withdrawal.

Ahmed et al. [21] reported 33% of 36 participants achieved discontinuation with a further 19% achieving and maintaining at least a 50% reduction at one month follow up. de Kuijper et al. [23] reported 37% of 98 participants achieved complete discontinuation with significant improvements in behaviours that challenge at 12 weeks follow up. Secondly, they reported re-prescribing at follow up after an initially discontinuing in 7% of participants. Ramerman et al. [32] reported 82% of the 11 participants in the deprescribing group, completely withdrew from risperidone.

#### Other outcomes

Four studies reported outcomes of physical and mental health and wellbeing.

One study by de Kuijper et al. [24, 81] and another by Ramerman et al. [32] reported positive effects of deprescribing antipsychotics on physical health parameters. de Kuijpers et al. [24, 81] reported a mean decrease of 4 cm waist circumference, of 3.5 kg weight, 1.4 kg/m^2^ BMI, and 7.1 mmHg systolic blood pressure at 12 weeks follow up after planned discontinuation, in 98 participants following complete discontinuation of antipsychotics over 14 or 28 weeks. Ramerman et al. [32] reported favourable group time effects on weight, waist, body mass index, prolactin levels and testosterone levels in 11 participants who completely discontinued risperidone. However, this study was underpowered and follow up was limited to 8 weeks. Both complete discontinuation and dosage reduction of antipsychotics were reported by de Kuijper et al. [24] to lead to a decrease in prolactin plasma levels and an increase in levels of C- telopeptide type 1 collagen (CTX), the bone resorption marker [24]. Ahmed et al. [21] reported an association of typical antipsychotic reduction with increased dyskinesia. However the follow up time for this study was 4 weeks compared to the study by de Kuijper et al. [24]which had a follow up period of 12 weeks and the study by Ramerman et al. [32] which had an 8 week follow up period. Conversely, Hassler et al. [79] reported weight gain in participants who discontinued zuclopentixol [79] in an inpatient setting. Two [24, 32, 81] of the three studies reporting positive physical health outcomes were carried out in community settings and the third study [21] involved participants in both hospital and community settings.

#### Integrated synthesis of RCTs

The evidence from RCTs regarding the effects of deprescribing on behaviours that challenge at follow up was mixed. The length of follow up was inadequate for the majority of studies with four studies reporting follow up periods of between four and eight weeks [20–22, 30, 32] and a further two studies reporting follow up at 12 weeks [23, 24, 28, 79, 81] and therefore it could not be established if successful deprescribing could be maintained in those studies reporting positive effects or no change on behaviours that challenge.

The evidence suggests that discontinuing or reducing the dosage of antipsychotics can have positive effects on physical health such as the reversal of antipsychotic markers for metabolic syndrome. The several subclasses of antipsychotics and variable doses at baseline may limit the robustness of this evidence. Methodological limitations across all RCTs included the use of small sample sizes and limited reporting of information about blinding procedures and methods to ensure allocation concealment. Two studies did not make use of blinding [21, 23]. The treating physician was involved in the sampling and recruitment of participants in two RCTs leading to possible selection bias [21, 32].

### Nonrandomised controlled studies

Seven nonrandomised controlled studies evaluated the effects of deprescribing antipsychotic medicines. Two studies were conducted in community settings [37, 42] and five studies were carried out in inpatient settings [35, 36, 39–41]. Sample sizes ranged from 6 to 80 participants, with participant ages, where reported, ranging from 7 years to 53 years, with 4 studies reporting outcomes for adults, one study reporting outcomes for adolescents (ages 10–19 years [80]) and one study reporting outcomes for children. The majority of participants were male ranging from 50 to 90% across studies. Length of follow up period varied from 8 weeks in one study to between 5 months and 12 months following discontinuation or maximum dosage reduction in those studies reporting. Two studies reported nonspecific variable follow up periods.

#### Behaviours that challenge

Two studies reported on changes in episodes or severity of behaviours that challenge. Zuddas et al. [42] reported progressive deterioration of behaviours in the 3 out of 10 adolescents and children participants who discontinued risperidone. A study by Swanson et al. [39] reported transient increases in ABC scores in 21 participants, 96% of whom had severe or profound intellectual disabilities, who discontinued risperidone and were co prescribed antiepileptic medication. However, this was not reported in the 19 participants who discontinued risperidone in the absence of antiepileptic medication.

#### Reduction /discontinuation outcomes

Two studies reported outcomes regarding numbers of participants who had their psychotropic medicines deprescribed. Zuddas et al. [42] reported that three children or adolescents discontinued risperidone although all three required the represcribing of an antipsychotic within 6 months following discontinuation.

A study by Gerrard et al. [37] comparing two groups of participants, reported a higher success rate for psychotropic medication reduction and discontinuation when this was carried out within a PBS framework. The authors reported that participants in the non-PBS group were more likely to have their medication increased following an initial reduction. Support was delivered by staff using a PBS framework for a minimum of three months post discontinuation or medication reduction. One patient required a medication increase or restart when supported by PBS. This compared to 66% of participants in the non-PBS group. However, evidence is limited by unequally matched groups in terms of intellectual disability [37] and the follow up times were variable.

#### Other outcomes

Four studies [35, 39–41] reported changes in dyskinesia scores following the deprescribing of antipsychotics. Aman and Singh [35] examined the effects of deprescribing typical antipsychotics on dyskinesias comparing a deprescribing group to a group that were not prescribed antipsychotics. The evidence was inconclusive although the deprescribing group was rated as having higher total dyskinesia scores.

Swanson et al. [39] reported transient increases in average Dyskinesia Identification System Condensed User Scale (DISCUS) scores after risperidone discontinuation with return to baseline 6 months after discontinuation. Wigal et al. [40] reported larger increases in DISCUS scores associated with greater dosage reductions of antipsychotics. Another study by the same authors measured the rates of dyskinesias during their medication review and dose reduction programme [41]. They reported that 63% of participants who discontinued antipsychotics and 29% of those who were receiving reduced dosages developed dyskinesias. In participants who were not medicated or where there was no change or increase in dosage, no dyskinesias were reported. All four studies were carried out in inpatient settings and most of the participants had severe or profound intellectual disabilities, ranging from 86 to 100%.

### Pre post study designs

Twenty-seven pre-post studies evaluated the effects of deprescribing psychotropic medicines, with all studies reporting interventions involving antipsychotics and 6 studies reporting on interventions on more than one class of psychotropic medication [38, 46, 49–51, 55] including anxiolytics (*n* = 3), antidepressants (*n* = 4), sedatives/hypnotics (*n* = 2), benzodiazepines (n = 2), anticonvulsants (*n* = 1) and lithium (n = 2) in addition to antipsychotics [46, 49–51, 55, 77]. Eight studies were conducted in community settings,17 studies were carried out in inpatient settings, one study involved a mix of both, and one study did not report setting. Sample sizes ranged from 6 to 250 participants, with participant ages, where reported, ranging from 5 years to 84 years, with 14 studies reporting outcomes for adults, 6 studies reporting outcomes for adolescents (ages 10--19 years [80]) and one study reporting outcomes for children. The majority of participants were male ranging from 50 to 100% where reported. Length of follow up period varied from 8 weeks to 15 years following discontinuation or maximum dosage reduction with 12 studies reporting variable follow up or not reporting follow up periods.

#### Behaviours that challenge

From 12 studies reporting on the effects of deprescribing psychotropic medicines on behaviours that challenge the findings are mixed [33, 44, 46, 48, 52–54, 57–59, 61, 67]. Branford [44] reported no deterioration in behaviours that challenge in 25% of 123 participants who underwent a reduction of antipsychotics. However, 42% did show a deterioration in behaviours that challenge, and for 33%, changes in behaviour were not reported. de Kuijper et al. [33] reported at 16 weeks post planned discontinuation, 6% of participants had shown improvement and 9% had a worsening of behaviour; at 28 weeks, these percentages were 9 and 15%, and at 40 weeks 21 and 7%, respectively. They also concluded that at 28 weeks, those who had not achieved complete discontinuation had significantly more worsening of behaviour than those who had successfully discontinued. Ellenor et al. [46] reported ABS scores for 54 participants which showed a slight increase in behaviours that challenge for all three groups; medication reduced, medication stopped and control group. Fielding et al. [48] reported that all but eight of 68 participants whose antipsychotic medication was reduced, achieved permanent dosage reductions while maintaining rates of behaviours that challenge similar to those observed prior to deprescribing. They also found that behaviours that challenge decreased or remained stable for the majority although they slightly increased for some. In two studies by Janowsky et al. [52, 53] 40% of 138 participants with severe or profound intellectual disabilities and 96% of 49 participants with severe or profound intellectual disabilities were reported to experience a relapse in behaviours that challenge. Jauernig et al. [54] reported a lower frequency of behaviours that challenge at follow-up compared to baseline in all 3 patients (100%) whose antipsychotic had been discontinued and in 15 patients (79%) of 19 who underwent dose reduction. Luchins et al. [57] reported an improvement in behaviour associated with the reduction in prescribing of antipsychotics. Marholin et al. [58] reported reversible changes in behaviours that challenge when chlorpromazine was withdrawn suddenly and then restarted 23 days later in 6 participants with severe intellectual disabilities. Matthews and Weston [59]reported over 50% of 77 participants who were on regular thioridazine experienced behaviours that challenge during or following discontinuation. Adverse events were significantly associated with the duration of previous thioridazine prescription. May et al. [61] evaluated the effects of deprescribing risperidone in people with severe and profound intellectual disabilities and reported transient worsening of behaviours that challenge in 39%, persistent worsening in 39%, and progressive improvement in 22% of participants. Stevenson et al. [67] reported that 48.7% experienced onset or deterioration in behaviours or mental ill-health following the deprescribing of thioridazine.

#### Reduction /discontinuation outcomes

Nineteen studies [33, 38, 43, 44, 46–55, 57, 60, 65–67] reported lower prescribing rates, complete discontinuation, or reduced dosages of psychotropic medicines,14 of which reported an evaluation of a clinical service involving multidisciplinary medication reviews with varying time periods for follow up [38, 44, 46–51, 54, 55, 57, 60, 65, 66]. Nine of the studies reported evaluations of medication reviews involving pharmacists [38, 44, 46, 47, 49, 51, 54, 60, 65]. A medication review programme by Branford [44] reported 16% of 198 adult participants withdrawn from antipsychotics and 28% maintained on reduced dosage of antipsychotics at 12 months follow up. One hundred and twenty-three of the 198 participants underwent a reduction of their antipsychotics and 25% discontinued antipsychotics while 46% were receiving reduced dosages at 12 months follow up. Gerrard et al. [38] reported that 24 psychotropic medications were stopped within their retrospective study; 20 of these were with PBS support and ongoing deprescribing continued at the time of publication of the study in 2020. de Kuijper et al. [33] reported 61% had completely discontinued antipsychotics at 16 weeks, 46% at 28 weeks, and 40% at 40 weeks. However, 32 % of participants who initially withdrew at 16 weeks were represcribed antipsychotics at 28 weeks follow up and 13% who withdrew at 28 weeks were represcribed antipsychotics at 40 weeks follow up. Studies by Findholt et al. [49] and Howerton et al. [50] reported a decrease in polypharmacy. A retrospective review by Luchins et al. [57] reported the reduction of antipsychotic prescribing was associated with prescribing other psychotropic medicines such as carbamazepine, lithium, and buspirone.

#### Other outcomes

Nine studies [34, 38, 45, 56, 62–65, 67] reported physical health and wellbeing findings, one of which also reported mental health and wellbeing outcomes. Ramerman et al. [34] reported improved physical health amongst those who completely discontinued antipsychotics, while social functioning and mental wellbeing initially deteriorated in those who incompletely discontinued; however, this was temporary, and they recovered at 6 months after planned discontinuation. In addition, they reported that participants who had completely discontinued had temporary decreases in mental wellbeing. Similar findings were reported by de Kuijper et al. [45] who reported a positive association between complete antipsychotic discontinuation with less-severe parkinsonism and lower incidence of health worsening compared with participants with incomplete discontinuation. Shankar et al. [65] reported no placement breakdowns or hospital admissions following antipsychotic deprescribing at 3 months follow up. This contrasts to Stevenson et al. [67] who reported 12% of participants were admitted to psychiatric assessment and treatment unit and 8% of participants required increased carer support following deprescribing of thioridazine. Two studies reported weight loss following deprescribing; Linsday et al. [56] reported the weight of 14 children returned to baseline at 12 and 24 months following discontinuing risperidone and Gerrard [38] reported a reduction in weight gain following deprescribing. Newell et al. [62–64] reported transient withdrawal dyskinesia in three studies monitoring participants during the reduction of typical antipsychotics.

#### Integrated synthesis of non randomised controlled and pre post studies

The non randomised controlled studies and pre post studies, although they have less methodological rigour, offered similar evidence to the RCTs through their use of evaluations of clinical services and longer follow up periods. In addition, the non-randomised controlled studies report more extensively on dyskinesias. One non randomised controlled study [37] and 14 pre post studies [38, 44, 46–51, 54, 55, 57, 60, 65, 66] evaluated clinical services providing deprescribing interventions, making use of a multidisciplinary model rather than the traditional medical model when reducing medication. Studies by Gerrard et al. [37, 38] and a case study by Lee et al. [75] reported that deprescribing outcomes for a range of psychotropic medicines were more successful alongside PBS compared to patients undergoing deprescribing interventions without this framework.

The external validity of the pre post studies was limited due to their lack of control or comparison group which would have improved the methodology. Well over half (67%) were conducted in inpatient settings. However, the reporting of deprescribing of psychotropic medicines other than antipsychotics allows for some tentative conclusions about the de-prescribing of psychotropics other than antipsychotics. Outcome from these studies suggest that deprescribing interventions within a multidisciplinary model may be associated with successful outcomes in terms of reducing and discontinuing psychotropic prescribing which could be maintained over a longer-term basis.

### Case studies

The effects of deprescribing on several classes of psychotropic medications were reported in 13 case studies [14, 68–76, 82] (3 separate case studies were reported in one paper). Five of these studies [14, 68, 75, 76] reported an association between successful discontinuation and improved quality of life. Another set of seven studies [69–74, 82] reported a range of adverse effects including delusions, hallucinations, inappropriate sexual behaviours, transient dyskinesias, self-harm, mania, aggression and catatonia following the deprescribing intervention. Lee et al. [75] described using flexible medication reduction within a PBS framework resulting in the discontinuation of risperidone.

## Discussion

We are mindful that some studies included psychotropic medicines that are no longer prescribed e.g. thioridazine, and some are rarely prescribed e.g. chlorpromazine. However the focus of our review was the psychotropic deprescribing process rather than evidence of effectiveness of deprescribing individual medicines. Hence the findings from these studies will still be relevant and add to the evidence base of the effects of deprescribing psychotropic medicines in people with intellectual disabilities. Overall the evidence from RCTs indicated that deprescribing interventions for antipsychotic medicines prescribed for the management of behaviours that challenge in people with intellectual disabilities may lead to a reduction in dosage and may be discontinued under some circumstances. Reducing and discontinuing antipsychotics may have positive health outcomes on physical health parameters. This is particularly important in this population as people with intellectual disabilities experience health inequalities with more co morbidities and reduced life expectancy [83]. Findings demonstrating the successful reduction in dosage and discontinuation of psychotropic medicines was also found in the other study designs. Although of reduced methodological rigour, the longer follow up times and the inclusion of other classes of psychotropic medicines in addition to antipsychotics of these studies, added to the evidence base. However, these positive findings need to be considered in the context of a lack of high quality RCTs.

Negative effects of deprescribing should be acknowledged. Firstly, RCTs reported relapsing of behaviours that challenge, although this evidence is limited by variable periods of follow up, and secondly dyskinesias were reported by four non randomised controlled studies [35, 39–41] and three pre post studies [62–64]. Furthermore, initial deprescribing is sometimes reversed with the represcribing of psychotropic medicines at follow up and therefore caution is needed when synthesising evidence from studies with a wide range of follow up periods. A variety of reasons were given for represcribing which included increases in episodes and intensity of behaviours that challenge, restrictiveness of setting and staff training [21, 30]. Physical discomfort is associated with behaviours that challenges [84]. Evidence suggests that people with intellectual disabilities are more susceptible to movement side effects of antipsychotic drugs [85]. As dyskinesias may be exacerbated by discontinuation of psychotropic medication this raises concerns that tapers will sometimes be aborted due to irritability and agitation that may be secondary to discontinuation effects rather than a relapse of symptoms related to the medicine’s efficacy.

Aside from represcribing psychotropic medicines, outcomes regarding the consequences of relapsing behaviours that challenge such as placement breakdown, hospital admission and increase in required carer support was limited to two pre post studies [65, 67]. Lack of consensus regarding optimal follow up time periods was a consistent theme across all included studies affecting the heterogeneity of the methodologies. This impacted on synthesizing the evidence regarding positive outcomes.

Although the findings from our systematic review suggests that deprescribing interventions in people with intellectual disabilities prescribed psychotropic medication, may lead to dosage reductions and the discontinuation of these medicines, it remains unclear how to optimise the circumstances for this to take place.

However, despite the limitations to the evidence base, it seems likely that planning before initiating the deprescribing process may be helpful. This could include staff training to ensure that other interventions for any transient increases in behaviours that challenge can be optimised together with plans for addressing the emergence or the worsening of dyskinesias. The evaluation of stakeholder experiences to understand barriers and enablers of this process may provide clarity.

There is a large variation in clinical practice of prescribers regarding discontinuation of psychotropic medication, both in terms of the deprescribing process and the individuals who are identified as suitable for deprescribing, This may be partially related to environmental factors as setting culture and attitudes of staff towards off-label antipsychotic medication use in people with intellectual disabilities [86]. How these decisions are made will likely impact on the success of deprescribing interventions.

In summary our findings suggest it is likely that there may be several factors affecting successful outcomes of the psychotropic deprescribing process. Enablers of the deprescribing process may be the views and clinical practice of clinicians, embracing a multidisciplinary approach, pre-planning of the deprescribing process including how to address emergence or worsening of movement effects, availability and quality of staff training, and stakeholder attitudes, including those of the individuals who are prescribed these medicines, towards the deprescribing process. Barriers to this process may be the perceived negative effects of the deprescribing process, limited knowledge of discontinuation effects of the individual psychotropic medicines, lack of input from carers and lack of understanding of the experiences of people whose psychotropic medicines have been reduced. Our review found there was a lack of evidence in the literature of shared decision making between people with intellectual disabilities and the healthcare team. The lack of literature about quality of life is reflected in our review as we were unable to report extensively on this outcome.

### Strengths and weaknesses of the methodology of included studies

With reference to reporting on medications, firstly, studies focused upon deprescribing one class or one specific type of medicine and did not address the co-prescribing of other psychotropic medicine, or an increase or decrease in dosage of these medicines. Secondly, studies did not report complete details regarding polypharmacy, where participants were co prescribed several medicines or were taking medicines available without prescriptions. This may have led to drug interactions and adverse drug reactions which could affect the outcomes of studies. Thirdly, the reporting of physical health medication and the prescribing and administration of PRN medication for the management of behaviours that challenge was missing from the included studies. Furthermore, there was frequent incomplete reporting of concurrent non-pharmacological treatments such as behavioural, psychological, and environmental interventions.

### Strengths and weaknesses of the methodology of the systematic review

A weakness of our methodology was that the second reviewer was restricted to independently screening 20% of titles, abstracts and full text papers and extracting data from 20% of included studies due to limited resources. Within this review, we focused upon deprescribing all psychotropic medicines in people with intellectual disabilities, rather than just antipsychotics which has been previously examined [15]. The adverse effects and effects associated with discontinuation may vary between classes of psychotropic medicines and within classes. Further, it should be noted that while the inclusion of single case studies and retrospective studies allowed for a more inclusive synthesis of the literature, studies of this type are prone to bias.

### Implications for clinicians and policymakers

Evidence based guidelines for prescribing psychotropic medicines in people with intellectual disabilities tend to focus on antipsychotics. There is a need to evaluate all psychotropic prescribing, including PRN, in people with intellectual disabilities to ensure that medication is being optimised and appropriate interventions are implemented within the multidisciplinary framework when addressing the management of behaviours that challenge.

We did not find evidence of involvement of patients, carers, and family within the development and process of deprescribing interventions. Whilst we have reported evidence suggesting that a multidisciplinary approach may be appropriate, policy makers and clinicians should be mindful to: (a) co-produce deprescribing interventions with people with intellectual disabilities to ensure they reflect their needs, which will, (b) help empower individuals to make informed decisions about their healthcare, and (c) facilitate full stakeholder engagement in shared decision making. It was noted that it was unclear as to how this was included within the intervention process within the included studies [14, 87, 88]. Co-production acknowledges that people with “lived experience” of using services are best placed to advise on what support and services will make a positive difference to their lives (Social Care Institute for Excellence, 2018).

Medicines optimisation is a patient centred framework forming part of routine clinical practice supporting patients to achieve best possible outcomes from their medicines by providing evidence based choices and ensuring medicines are as safe as possible [89]. Deprescribing should form part of medicines optimisation and incorporate routine monitoring to help improve health outcomes as evidence suggests that people with intellectual disabilities have poorer health outcomes [90]. Embracing a multidisciplinary approach and co-producing robust effective deprescribing processes with all stakeholders at the individual and service level may contribute to improved health outcomes reducing exposure to adverse effects. Routine monitoring within the medicines optimisation framework must address not only the effectiveness and adverse effects of medicines, but also discontinuation effects and possible relapses facilitating prompt medication review.

### Implications for future research

Studies addressing quality of life measures would address the absence of this in the literature. We recommend that future research should focus on studies addressing confounding factors that we have highlighted above, namely the lack of reporting of other co-administered interventions. These interventions include the prescribing of other classes psychotropic medicines which were not the subject of the deprescribing intervention, the prescribing of PRN medication, psychological, environmental, and behavioural interventions. The length of the follow up period may have a significant impact on whether deprescribing can be deemed as successful in sustaining long term reduced reliance on psychotropic medication and temporary discontinuation effects and the re-emergence of behaviours that challenges. We therefore suggest longer follow up periods are needed within future studies. We also recommend that future research should also consider the feasibility of deprescribing all classes of psychotropic medicines in routine clinical practice in a range of settings, and with children, adolescents, and adults. Further studies of stakeholder experiences to identify enablers of deprescribing and best practice in involving people with intellectual disabilities and their carers in decisions about their medicines would be welcome. Recruitment and sampling challenges will need to be addressed in future research ensuring balance and reporting of age, gender, ethnicity, and level of intellectual disability within both inpatient and community settings. Further research to increase the knowledge of discontinuation symptoms of the various psychotropic medicines would be helpful when planning psychotropic deprescribing.

Further studies looking at enablers and barriers of the psychotropic deprescribing process, including addressing the impact of attitudes towards deprescribing of clinicians and carers on the success of deprescribing interventions, would be welcome as this could potentially influence initial decisions to implement deprescribing in individuals affecting outcomes.

Finally, it would be helpful if future studies exploring psychotropic deprescribing consistently reported outcomes regarding complete discontinuation, or greater dosage reduction, rate of represcribing, improvement and deterioration of behaviour and emergence of adverse effects.

### Other information

Protocol and registration: The review protocol was registered on 19th December 2019 with PROSPERO, the international prospective register of systematic reviews (registration number CRD CRD42019158079). https://www.crd.york.ac.uk/prospero/display_record.php?ID=CRD42019158079.

Amended versions of the protocol were published on 7th July 2020, 17th September 2020, 30th September 2020 and 22nd March 2021. For further details regarding amendments please see link above.

## Supplementary Information

**Additional file 1.**

## Data Availability

The data that support the findings of this study can be found in the summary table (Table 4), the quality assessment table (Table 5) and the raw data supplementary file (Table 8).
