# Supplementary material for: Deprescribing psychotropic medicines for behaviours that challenge in people with intellectual disabilities: a systematic review
Source: BMC Psychiatry. 2023 Mar 28;23:202. doi: 10.1186/s12888-022-04479-w (PMC10044393; doi:10.1186/s12888-022-04479-w)
Supplement: Supplementary file 1 — Additional file 1. [file 12888_2022_4479_MOESM1_ESM.pdf]

Article characteristics				
**Reference No**	**Title**	**Author**	**Journal**	**Year**
1	A Review of Antipsychotic Drugs Prescribed for People with Learning Disabilities Who Live in Leicestershire	Branford D	Journal of Intellectual Disability Research	1996
2	Assessment of Qtc Prolongation Following Thioridazine Withdrawal in a Developmentally Disabled Population	Brahm, N. C., et al	Journal of Clinical Psychopharmacology	2003

3	An Open-Label Discontinuation Trial of Long-Term, Off-Label Antipsychotic Medication in People with Intellectual Disability: Determinants of Success and Failure	de Kuijper et al	Journal of Clinical Pharmacology	2018
4	An Open Label Discontinuation Trial of Long-Term Used Off-Label Antipsychotic Drugs in People with Intellectual Disability: The Influence of Staff-Related Factors	de Kuijper, G. M., and P. J. Hoekstra	Journal of Applied Research in Intellectual Disabilities	2018

5	Pharmacist Impact on Drug Use in an Institution for the Mentally Retarded	Ellenor, G. L., and P. A. Frisk.	American Journal of Hospital Pharmacy	1977
6	Effects of Data-Based Interdisciplinary Medication Reviews on the Prevalence and Pattern of Neuroleptic Drug Use with Institutionalized Mentally Retarded Persons	Ferguson, Donald G., et al	Education & Training of the Mentally Retarded	1982

7	An Assessment Program to Reduce Drug Use with the Mentally Retarded	Fielding, L. T., et al	Hospital & Community Psychiatry	1980

8	Impact of Interdisciplinary Team" Review on Psychotropic Drug Use with Persons Who Have Mental Retardation	Findholt, N. E., and C. G. Emmett	Mental Retardation	1990

9	Delivering STOMP in a community learning disability treatment team	Gerrard D	Hospital Pharmacy Europe	2020

0	Psychotropic Medications in Community Based Individuals with Developmental Disabilities: Observations of an Interdisciplinary Team	Howerton K et al.	Mental Health Aspects of Developmental Disabilities	2002
11	A Clinical Pharmacy Service to Reduce Psychotropic Medication Use in an Institution for Mentally Handicapped Persons	Inoue F et al	Mental Retardation	1982

12	Antipsychotic Withdrawal-Induced Relapse Predicts Future Relapses in Institutionalized Adults with Severe Intellectual Disability	Janowsky	Journal of Clinical Psychopharmacology	2008
13	Relapse of aggressive and disruptive behavior in mentally retarded adults following antipsychotic drug withdrawal predicts psychotropic drug use a decade later.	Janowsky	Journal of Clinical Psychopharmacology	2006

14	Evaluation of an Interdisciplinary Review Committee Managing the Use of Psychotropic Medication with People with Intellectual Disabilities	Jauernig, R., and A. Hudson	Australia and New Zealand Journal of Developmental Disabilities	1995
15	Reducing Psycho-tropic Drug Use in an Institution for the Retarded	LaMendola, W, and Zaharia, E and. Carver, M	Hospital and Community Psychiatry	1980

16	Discontinuation of Risperidone and Reversibility of Weight Gain in Children with Disruptive Behavior Disorders	Lindsay, R. L., S. Leone, and M. G. Aman	Clinical Pediatrics	2004

17	Factors Associated with Reduction in Antipsychotic Medication Dosage in Adults with Mental Retardation	Luchins, D. J., D. M. Dojka, and P. Hanrahan.	Clinical Pediatrics	2004

18	Implementation of a Psychotropic Drug Review Service in a Mental Retardation Facility	Marcoux, A. W	Hospital Pharmacy	1985
19	Experience of Thioridazine Use before and after the Committee on Safety of Medicines Warning	Matthews T., and S. N. Weston	Psychiatric Bulletin	2003

**20**	Withdrawal of Chronic Chlorpromazine Medication: An Experimental Analysis	Marholin D et al	Journal of Applied Behavior Analysis	1979

21	A Study of the Clinical Outcome of Patients with Profound Mental Retardation Gradually Withdrawn from Chronic Neuroleptic Medication	May, P., et al.	Annals of Clinical Psychi	1995
22	Dynamics of Lip Dyskinesia Associated with Neuroleptic Withdrawal	Newell, K. M., et al	American Journal of Mental Retardation	2000

23	The Changing Effector Pattern of Tardive Dyskinesia During the Course of Neuroleptic Withdrawal	Newell, K. M., et al	Experimental & Clinical Psychopharmacology	2001
24	Onset of Dyskinesia and Changes in Postural Task Performance During the Course of Neuroleptic Withdrawal	Newell, K. M., et al	American Journal of Mental Retardation	2002

25	Changes in Health-Related Quality of Life in People with Intellectual Disabilities Who Discontinue Long-Term Used Antipsychotic Drugs for Challenging Behaviors	Ramerman, Lotte, Pieter J. Hoekstra, and Gerda Kuijper	Journal of Clinical Pharmacology	2019

26	A Structured Programme to Withdraw Antipsychotics among Adults with Intellectual Disabilities: The Cornwall Experience	Shankar, R., et al.	Journal of Applied Research in Intellectual Disabilities	2019
27	Tranquilizer Reduction Trials in a Residential Program for Persons with Mental Retardation	Spreat S et al	Hospital & Community Psychiatry	1993

28	Withdrawal of Antipsychotic Drugs from Adults with Intellectual Disabilities.	Stevenson, C., et al.	Irish Journal of Psychological Medicine	2004

Country of orgin	Study objectives
England	To identify Factors associated with the successful or unsuccessful withdrawal of antipsychotic drug therapy prescribed for people with learning disabilities
USA	

The Netherlands	To investigate the influence of factors potentially associated with successful discontinuation, including changes in environmental circumstances.
The Netherlands	•The influence of staff-related factors on results of discontinuation trajectories and the behaviour of their clients.<br>•We hypothesized that more negative feelings towards challenging behaviour of clients and less knowledge towards effects of psychotropic drug use of support professionals, and higher severity of behavioural symptoms as assessed by clinicians predicted less chance of successful discontinuation.<br>•Other questions were whether there was a relationship between support professionals' feelings and participants' behaviour, and whether we could identify factors which were related to clinicians' judgements of worsening of behaviour during discontinuation

USA	1. Create a direct communication of the patient's behavioral or psychological status to the physician by the direct care staff.<br>2. Open new communication lines among other staff (i.e., training staff) and the physician.<br>3. Relieve the pressures on the nursing department, applied by ward staff, to procure drugs for the treatment of all be¬havioral problems.<br>4. Allow the pharmacist to provide the physician with:<br>a. a complete and accurate drug history;<br>b. an evaluation of past and present drug therapy;<br>c. recommendations regarding drug therapy which reflect documented therapeutic techniques;<br>d. specific drug information; and<br>e. clinical observations for the appearance of adverse drug reactions.<br>5. And finally, provide a means of follow-up and evaluation of changes implemented in therapy.
USA	SERVICE EVALUATION

USA	two-phase assess-ment program that has enabled one institution, Brain-erd (Minn.) State Hospital, to comply with these guide-lines for its developmentally disabled residents.

USA	

U.K. England	

USA	a two year experience of an IDT in addressing the issue of polypharmacy involving psychotropic medications in a cohort of individuals with MR/ DD living in Orange County, California. For purposes of this study, polypharmacy was defined as utilization of two or more psychotropic medications on a daily basis.
Canada	This paper describes the clinical pharmacy activities having significant impact on the drug utilization pattern in a MRF without reading major changes in life skills pro-gramming or pharmacy staff. Further, it results in a reduc-tion of reliance on psychotropic medication for the staff to manage maladaptive behavior problems. Also, the main reason for the use of psychotropic medications in our facility is to control maladaptive behavior such as aggres-sion, destructive acts, and self-abuse.

USA	to determine whether an unsuccessful antipsychotic drug withdrawal attempt predicted future drug withdrawal–induced relapses in institutionalized individuals with severe and profound IDD
USA	The purpose of this study was to determine if relapse during orafter antipsychotic drug withdrawal in mentally retarded adultspredicts continuing antipsychotic drug use an average of a decadelater.

Australia	•To evaluate an Interdisciplinary Review Committee Managing the Use of Psychotropic Medication with People with Intellectual Disabilities<br>•In addition to seeking to determine the minimal effective dose for any particular medication, it was an aim of the Committee to minimise, and ideally eliminate, polypharmacy with respect to psychotropic medication.<br>•An emerging challenge here was the identification of that medication most appropriate to the control of the particular behaviours of concern.<br>•Some drugs, such as the anticonvulsants, were arguably not optimally suited to behaviour management, and the decision to cease administration might have been relatively straightforward in the absence of a history of seizures, or where the client had been seizure-free for an extended period.
USA	Evaluation of drug evalua¬tion program at Glacial Ridge Train¬ing Center.

USA	The objective of this study was to examine the data on weight gain or loss in participants of ex-isting research studies assessing effects of risperidone on disrup-tive behavior.

USA	In-the present study records of all individuals admitted to a 75-bed unit of a state facility for.adults with developmental disabilities were examined. The unit func-tioned as the admitting-unit for individu-. alawith Mental retardatiokand a mental 'illness or with mental retardation and sev&e behavior problems The study was &signed to address several questions: (a) Were cnr,:ent clinical practices of decreas-ing antipsychotic usage being followed? (b) Was there-a difference in individuals who were able to have antipsychotic medi-cation reduced/discontinued versus thou who had increases? (c) Because the effi-cacy of other medications to treat physical aggression and self-injurious behavior has been established (Luchins & Dojka, 1989), could these medications be used in place of antipsychotics, thereby lowering the likelihood of tardive dyskinesia?

USA	
wales	

USA	Clinical Outcome of Patients with Profound Mental Retardation Gradually Withdrawn from Chronic Neuroleptic Medication<br>The study reported here suggests that behav¬ioral deterioration seen in profoundly retarded indi¬viduals being tapered from neuroleptic medication is "transient" more frequently than is generally appre-ciated. Lack of appreciation of the transient nature of behavioral deterioration may be related to the pro-longed nature of the transiency in some cases and also possibly to deficient knowledge of alternative ways to manage the emergent behaviors. Attempts to differ-entiate transient "withdrawal" phenomena from neuropsychiatric disease/psychosis are discussed.
USA	In the present study we report on an examination of the dynamics of lip motions in tardive dyskinesia as a func-tion of progressive medication reduction and eventual withdrawal.

USA	•This article reports an analysis of the pattern of effector disorders in TD over a drug medication reduction and eventual withdrawal protocol in a group of adults with MR that had been on a chronic regimen of typical neuroleptic medications.<br>•We examined the changing pattern of TD as measured by the Dyskinesia Identification System: Con-densed User Scale (DISCUS), a scale that has established validity and reliability for measuring TD in populations with MR (Sprague et al., 1989; Sprague, White, Ullmann, & Kalachnik, 1984).<br>•The data analyzed were from three time periods of a prospective medication reduction and with-drawal protocol: a baseline assessment prior to medication reduction, the assessment at the point of peak dyskinesia following neuroleptic withdrawal, and a follow-up assess-ment at the point of lowest dyskinesia following neuroleptic withdrawal.
USA	In this experiment we examined the postural dynamics in adults with mental retardation at three time points during the course of a neurolep-tic withdrawal protocol:<br>(a) Our first examination was at baseline, when the participant was on their regular individualized dose of neuroleptic medi-cation;<br>(b) the second measurement was taken during the course of medication reduction/with-drawal at the point of peak withdrawal dyskinesia as determined by the total score from the DIS-CUS rating scale (Dyskinesia Identification Sys-tem: Condensed User Scale–Sprague, Kalachnik, & Slaw, 1989; Sprague, White, Ullman, & Kalach-nik, 1984). Peak withdrawal dyskinesia is the time point that by definition reveals the most extreme departures from healthy behavior due to the med-ication reduction.<br>(c) The third point was at fol-low-up, at least 3 months after the completion of medication withdrawal, coinciding with the point of lowest dyskinesia as determined by the DIS-CUS. These three time points provided the op-portunity to assess the major changes over time of the effects of neuroleptic withdrawal on the postural stability associated with varying degrees of dyskinesia.

The Netherlands	The aim of the study was to investigate the effects of discontinuation of long-term used antipsychotics prescribed for the management of challenging behaviours, on health-related quality of life.

England	•In Cornwall, the authors decided to develop a structure around the withdrawal pathway before embarking on antipsychotic withdrawal in adults with intellectual disabilities to support the NHS England STOMP initiative.<br>• The project was set out as a quality improvement (QI) initiative using the "Plan, Do, Study, Act (PDSA)" model (NHS Improvements, 2018).<br>•The "Plan" was to identify all people with intellectual disabilities on APT in Cornwall, having no major mental disorder. The aim of the first QI cycle was to reduce the antipsychotic burden by 20% of the identified target population in one year and then realign expectations for next cycles subsequently.<br>•"Do" was to undertake a structured reduction plan as identified in the methods section.<br>•"Study" was to collect data post-attempt and compare to see if 20% reduction was achieved and if not why not. It would also allow an opportunity to reflect on the impact, barriers and what went well of the change and what was learned including looking into what worked and what did not and why.<br>•"Act" was to find solutions to overcome the challenges and to plan the next cycle.<br>• QI methodology was the back- ground for the current project to guide and evidence improvements.
USA	

Glasgow, Scotland	This study critically examines whether antipsychotic drug withdrawal is beneficial.

Study char
**Design**
Medication review of identified cohort of patients with a plan to withdraw or reduce dose of antipsychotic medeicines.
medication review programme

•This was an open label discontinuation study. We combined data from 2 discontinuation studies:
1) discontinuation of long-term risperidone prescribed for challenging behavior (Netherlands Trial Register NTR5509) and
2) a discontinuation study involving all other antipsychotics (NTR5519).
•Potential participants received a treatment proposal from their physician involving an attempt to discontinue antipsychotics.
•We prospectively investigated the influence of factors potentially associated with successful discontinuation, including changes in environmental circumstances.
•Eligible participants could be
•either sex and any ethnicity
•aged greater than 6 years,
•functioning below an IQ level of 70,
•and had used one or more antipsychotics for more than one year for challenging behavior.
•Excluded were subjects with schizophrenia, a bipolar disorder, or an affective psychosis according to the Diagnostic Statistic Manual 4th Edition, Text Revision or International Code of Diseases 10th Edition.
•Another exclusion criterion was an unsuccessful attempt to discontinue the antipsychotic in the previous 6 months, as a subsequent attempt to discontinue after that short interval would be unlikely to be successful.

open label discontinuation trial
Data was combined from 2 discontinuation studies:discontinuation of rieperidone prescribed for challenging behaviour (see Rammerman et al's RCT) and a discontinuation study involving all other antipsychotics

Pharmacist involvement on a team developed to monitor drug use in an institution for the mentally retarded is described, and the results of the team's efforts'are reported.

two-phase assess-ment program

medication review programme

medication review

An evaluation of the IDT and treatment program for psychotropic polypharmacy in a community based cohort of individuals with MR/DD.
medicaton review

studying an adult institutionalized intellectually disabled pop-ulation, with whom we conducted a 15-year retrospective records review.

medication review programme
unclear

The authors followed up 14 subjects who were in studies of risperidone for management of disruptive behavior disorders. The subjects exited after a mean exposure of 8.9 months because of excessive weight gain, or excessive appetite, or insufficient clinical response. Weight was monitored for the full cohort before risperidone treatment, at termination, and (for various subgroups) at 3, 9–12, and 24 months after termination.

medication review programme

medication rview programme
retrospective case note analysis

Antipsychotics were withdrawn and readministered using a double-blind B-A-B (drug-placebo-drug) design.

deprescribing study
The lip movements associated with dyskinesia in adults with mental retarda-tion were investigated through a dynamic analysis at medication baseline, at the points of the highest level of withdrawal dyskinesia as indexed by the DISCUS rating scale, and at the lowest level of dyskinesia following complete withdrawal of the medication

The primary focus of this study was to investigate if and how the form of dyskinesia changes over time as a function of progressive withdrawal of neuroleptic medication. To examine this question we analyzed the contribution of each individual item score from the DISCUS to the total DISCUS score at each of three assessment time periods:<br>(a) at base-line while on the maintenance dose of neuroleptic medica-tion,<br>(b) at the point of peak TD following medication withdrawal, and<br>(c) at the point of lowest dyskinesia fol-lowing complete neuroleptic withdrawal.<br><br>Three sets of anal-yses were performed.<br>First, we examined mean changes in total dyskinesia and specific dyskinesia items over the three time periods.<br>Second, we examined changes in the pattern of interitem correlations over the three time periods.<br>Third,we used multiple regression to examine which items uniquely contributed to the total score at each of the three time periods<br>These 26 patients were selected for the present study on the basis of one or more of the following criteria:<br>(a) They were selected by their treatment teams to be given a trial off of their neuroleptic drug,<br>(b) they were referred to our laboratory for dyskinesia assessment over the course of the reduction and withdrawal of their neuroleptic medication,<br>(c) they completed their prescribed course of neuroleptic reduction and withdrawal, and<br>(d) their parent or guardian gave consent for the data derived from their clinical assessments of dyskinesia to be included in this study.
Over the course of the study period, some mem-bers of this initial group either had the neuroleptic withdrawal terminated before complete dose with-drawal, were switched to treatment using an atyp-ical neuroleptic agent prior to the end of the 1-year period of evaluation postwithdrawal, or were unable to comply with the testing demands re-quired for the postural task used in the present study. This left a total of 20 participants (15 males [75%], 5 females [25%]) who underwent neuro-leptic dose reduction and withdrawal for the pre-sent study. The baseline neuroleptic medications for the participants who completed the study were haloperidol (n = 5), thioridazine (n = 7), chlor-promazine (n = 2), trifluoperazine (n = 3), and loxapine (n = 3).<br>Selection criteria were: (a) referral by clinical treatment team for systematic neuroleptic dose withdrawal; (b) parent/guardian consent to with-drawal; (c) history of continuous treatment with typical neuroleptic agent for at least 3 years prior to start of the study; (d) not receiving adjunctive treatment with an anticholinergic agent; and (e) between the ages of 20 and 55 (omitting child and geriatric participants). All 42 persons who met these criteria were initially enrolled in the study.

•data from two discontinuation trials: an open-label trial of various antipsychotic drugs and a double-blind trial of risperidone.
• In both studies antipsychotics were discontinued in 14 weeks, with steps of 12.5% of the baseline dosage every two weeks. •Health-related quality of life was measured at baseline and at 16 weeks and 40 weeks after baseline, by means of the RAND-36 (domains on physical well-being, role limitations caused by physical or emotional problems, vitality, pain, mental well-being, social functioning, general health and changes in health). •Participants who had completely discontinued antipsychotics according to the scheduled discontinuation and were still free of use at 40 weeks were compared with those who had incompletely discontinued.

In Cornwall, UK, the present authors proceeded with the withdrawal programme in several steps that are described here.
At the heart of our programme was the wider stakeholder involvement including people with intellectual disabilities and their carers (both family and paid carers), GPs, community pharmacists and community learning disability team (CLDT) members.
Step1: Primary care and identification of the cohort
Step 2: Involving all stakeholders
Step 3: Secondary care
Step 4: Development of tools
Step 5: Assessment of patients for withdrawal
Step 6: Follow-up and contingency plan

•For 101 people (84.9%) the changes in thioridazine were made as a result of the CSM advice.
•For 14 people (11.8%) there were other clinical indications for the withdrawal of thioridazine, and for four people (3.3%) it was unclear whether withdrawal of thioridazine was solely due to the new advice or not
•At the time of the study, the necessary interventions to withdraw the thioridazine had been completed in 84% of cases, and were still in progress for 16%.
•Withdrawal was initiated by a GP in 33 cases (28%), and then referred to learning disabilities psychiatry.
•For the remainder it was initiated by a learning disabilities psychiatrist.
•The method of thioridazine withdrawal was decided by the responsible doctor based on each individual’s identified clinical needs and thioridazine dose.
•The thioridazine was discontinued at once for nine people (7.6%), was gradually withdrawn for 27 people (24.4%), was switched at once to an alternative a n t i p s y chotic drug for 52 people (43.7%), and was gradually s w i t ched to an alternative antipsychotic drug for 22 people (18.5%). The exact method of withdrawal was unclear for nine people (7.6%).
•All individuals remained under the care of a learning disabilities psychiatrist during this process;therefore subsequent changes to the original planned method of thioridazine withdrawal (ie. prescription of an alternative antipsychotic drug when none had originally been planned) were decisions made on the basis of individual clinical need.
•Thioridazine was withdrawn in 4 different ways: discontinued at once,gradual discontinuation, switched at once to an alternative drug, gradual switch to an alternative drug

acteristics

Setting	Recruitment procedures and Sampling Approach
inpatient and community	
not reported	

Community •Study setting was the living facilities of 6 care-providing organizations.<br>•Four of the six organizations also provided data for the open-label discontinuation of risperidone.<br>•Living situation Congregated center: 70.9% Community center: 22.2% Community living facility: 6.8% Living situation, group size 1–4 persons; 18.5% 4–8 persons: 55.6% 8–12 persons: 22.2% Independently: 3.7%	
Six residential care providing organsiations in the community	Potential participants received a treatment proposal from their physician involving an attempt to discontinue antipsychotics.

•The facility is an institution with approximately 475 patients. It provides 24-hour residential, medical and treatment programs not available to these patients in their communities.<br>•Approximately 80% of the patients are severely<br>or profoundly mentally retarded, and 20% are mildly to<br>moderately retarded. In addition, 25% have cerebral palsy<br>and 60% are epileptic.	
inpatient	

inpatient	

inpatient	

community	

community	The individuals in this study represent the first 159 individuals referred to the clinic for assessment of polypharmacy involving psychotropic medications. Referrals to the clinic were generated by the Regional Center of Orange County, a non-profit agency which contracts with the State of California to provide services for persons with MR/DD. Under the auspices of the Regional Center, it was possible to access the pharmacy database from the Orange County Health Agency, State Department of Mental Health.<br><br>Of 8,464 people under the management of the Regional Center, 3,901 were determined to be on at least one psychotropic medication. By utilizing the monthly pharmacy database, the prescription profile of
Inpatient. The MRF in this study is a 400-bed govern-ment facility. Its primary function is to provide training to mentally handicapped individuals to prepare them for community living	

residential care<br>Inpatient. The subject group consisted of 57 residents of the Murdoch Center, a 583-bed North Carolina intermediate-care facility for persons with intellectual disability located in Butner, NC	
inpatient	

inpatient	•In late 1991, there were 69 clients receiving chemical restraint at the Training Centre.<br>•These clients lived in seven different units in the Centre.<br>•Because inclusion in the Committee's review process involved direct-care staff in the daily measurement of the occurrence of challenging behaviour, a stratified sampling procedure was used to ensure that clients under review were spread across units. Within units, clients were essentially randomly selected, although factors such as a current health problem or an imminent move to another unit led to a postponement of review for that client.<br>•The intent was to begin with a small number of selected clients and progressively move to a review of all 69.
inpatient	

community	Each subject was taking open-la-bel risperidone from 2.5 to 12 months (allowing for the possibil-ity of assignment or not to a placebo condition for the first 6 weeks), with a mean of 8.9 months (SD = 4.0). The mean dose before discontinuation of risperidone was 0.0378 mg/kg/day (SD = 0.0172) with a mean daily dose of 1.61 mg/day. For the current study, we obtained physical mea-surements as follow-up to the open-label studies in which all 14 children participated.<br>All subjects experienced weight gain during open-label risperidone use and discontinued risperidone at the end of their trial. For 8 subjects, excess weight gain was the reason given by par-ents for discontinuation, while for 2 subjects, the parents reported only

inpatient	

inpatient	
community	

inpatient	

inpatient	
inpatient	These indi-viduals were referred by their clinical interdisciplinary treatment teams for neu-roleptic medication withdrawal as part of a standard program of psychotropic medication treatment monitoring in place at the facility.

Inpatient. All participants were residents of a single intermediate care facility for persons with MR and were drawn from an available pool of 370 persons during the study period.<br>At the start of the study period, 122 persons were receiving treatment with typical neuroleptic agents.<br>Over the course of the study period (approxi-mately 3 years), a sample of 26 patients were selected from this available pool of persons receiving neuroleptic medication	Over the course of the study period (approxi-mately 3 years), a sample of 26 patients were selected from this available pool of persons receiving neuroleptic medication. These 26 patients were selected for the present study on the basis of one or more of the following criteria:<br>(a) They were selected by their treatment teams to be given a trial off of their neuroleptic drug,<br>(b) they were referred to our laboratory for dyskinesia assessment over the course of the reduction and withdrawal of their neuroleptic medication,<br>(c) they completed their prescribed course of neuroleptic reduction and withdrawal, and<br>(d) their parent or guardian gave consent for the data derived from their clinical assessments of dyskinesia to
Inpatient. All participants were residents of a single In-termediate Care Facility for the Mentally Retarded (ICF/MR) and, thus, were drawn from a single available participant pool of 370 persons at the start of the study.	

community	•In total, nine organizations (two mental health care organizations and seven service providers of intellectual disability care, including medical and psychological services) participated in the studies.<br>•Two of the seven service providers participated in both studies. These two organizations included their clients using risperidone into the double-blind study and the other antipsychotic drug users into the open-label study.<br>•In both studies, participants received a proposal for discontinuation from their physician.

community	
inpatient	

community. 22(18.5%) lived with parents or family members and one person (0.8%) with a spouse. A total of eight people (6.7%) lived in a residential or nursing home, 61(52.1%) lived in their own tenancy with 24-hour staffing, eight (6.7%) in their own tenancy with less than 24-hour staffing and six people (5%) lived alone with no support.	All patients known to the Greater Glasgow intellectual disabilities psychiatric service who were taking thioridazineafter December 2000 were managed in keeping with the new clinical advice.<br>•In September 2001, these individuals were identified and their intellectual disabilities psychiatry case notes examined.<br>•A purpose-designed data collection form was used. Case notes were examined during the period of September 2001 to February 2002.<br>Data was entered from each form onto PC for analysis using SPSS Version 10.

What is measured?

•Three rating scales were administered prior to any reduction of drug therapy.
•These included the Aberrant Behaviour Checklist (ABC), the Psychopathology Inventory for Mentally Retarded Adults (PIMRA) and the Reiss Screen for Maladaptive Behaviour.
•The ABC is a brief rating scale designed to assess the effect of drug changes upon challenging behaviour in people vAxh leaming disabilities.
•Scores from particular statements are grouped into five subscales of irritability, lethargy, stereotypy, hyperactivity and inappropriate speech.
•The scale is claimed to have good intemal consistency and test-retest reliability.
•The PIMRA is a checklist of behaviours intended to assist in the identification of psychopathology in people with leaming disabilities.
• The item content of the PIMRA is based on the major categories from the DSM-III-R (APA 1987) and uses a scoring system developed by Matson (1988).
•The Reiss Screen for Maladaptive Behaviour comprises 38 questions about maladaptive behaviours which are rated according to their degree of severity. The ratings are converted into scores, and grouped into eight separate subscales of behaviours and diagnostic categories (Reiss 1988)
•In addition to the information above, further information was available about the patients from the Leicestershire Information Co-ordination Centre (LICC).

ECG

The primary outcome measure was achievement of complete discontinuation at 16 weeks.<br>Secondary outcome measures were achievement of complete discontinuation at 28 weeks and 40 weeks and changes in the Aberrant Behavior Checklist (ABC) and its 5 subscales, ie, irritability, lethargy, stereotypic behavior, hyperactivity, and inadequate speech. The ABC was completed by the main caregiver. We defined a change of >8 points in ABC total score (0.33 SD) from baseline as clinically relevant
•The primary outcome was achievement of complete discontinuation<br>at 16 weeks.<br>•Secondary outcomes were achievement of complete discontinuation at 28 and 40 weeks, the Aberrant Behaviour Checklist<br>(ABC) and its five subscales, that is, irritability, lethargy, stereotypic<br>behaviour, hyperactivity and inadequate speech, the Clinical Global<br>Impression scale-severity (CGI-S) and the Clinical Global Impression<br>scale-Improvement (CGI-I) with regard to severity of psychiatric<br>symptoms and challenging behaviour.<br>•The ABC was completed by the main caregiver. We defined changes of >8 points in ABC total scores (0.33 SD) as clinically relevant.<br>•The CGI-S and CGI-I were completed by the main clinician, that is, the behavioural scientist or the intellectual disability physician.

•Alterations in the Use of Drugs. Data on drug and dosage changes made by the BRC for medications affecting behavior control were divided into four classifications. These included medication usage for:
(1) antianxiety and antidepressant agents;
(2) antipsychotic agents;
(3) sedative-hypnotic agents; and
(4) miscellaneous agents.

Comparisons were made between the time the project was initiated in January 1974 and in January 1976. All changes in medications used for behavior control were made by the BRC. Statistical analysis of the data was performed by the Student t-test.

•Impact. on Drug Expenditures. The impact of the BRC on drug expenditures was calculated through comparing costs of medications used for behavior control "before" to "after two years" of the program. Only those changes in therapy initiated by the BRC were considered. Because this institution exclusively uses the unit-of-use Blue Book cost (the Blue Book price for the package size actually purchased by the institution) in billing for medications, this cost was used in determining the impact on drug expenditures. In order toeliminate the effects of inflation on medication costs, 1976 Blue Book costs were used for both the 1974 and 1976 drug use data.

•Impact on Patient Care. The Adaptive Behavioral Scales17 (ABS) maladaptive behavioral results were used for evaluating the effects of this program on patient care. Mal-adaptive behaviors in the ABS are defined as: physical aggression to others or properties; self-abusive behavior; inappropriate sexual behavior; stereotype behavior; verbal aggression; trustworthiness; and hyperactivity. As this study was retrospective, only patients who had had ABS tests performed within six months prior to entering the BRC were included in the evaluation.

Daily number of incidents of adverse behaviours
Number of participants who discontinued or changed dose of psychotropic medicines

Number of patients taking antidepressants, anxiolytics and Antipsychotics and Number of patients receiving polypharmacy (defined in study as 2 or more psychotropic medicines)
Cost savings based on medicine prices

Number of medicines stopped, Number of medicines restarted.
For antipsychotic prescriptions- FBC, U+Es, LFT, TFT, Lipids, Glucose/HbA1c, prolactin, BP, weight, pulse and ECG

Medicines stopped and started
number of dosage reductions of psychotropic medicines over a 5 year period

Number discontinuing antipsychotic medication<br>Number of episodes of challenging behaviour
number of people discontinuing antipsychotic medication

•A number of instruments were developed or adapted for use by the Committee.<br>•These included a standardised client information and consent procedure; Behaviour Monitoring Record forms; the Aberrant Behaviour Checklist (Amu, Singh, Stewart, & Field, 1985); Psychotropic Medication and Behaviour Monitoring form; and a medication side-effects checklist abbreviated, to simplify comple-tion, from the MOSES scale (Kalachnik, 1988).<br>•the number of people reviewed, the number of people with reduced medication doses, number of people who discontinued medication.<br>The data collected were frequency counts of the relevant challenging behaviours and were taken by unit staff for a period of at least one month using direct observation procedures.
Percentage of patients prescribed psychotropic medicines, percentage of patients prescribed major and minor

For the current study, we obtained physical mea-surements as follow-up to the open-label studies in which all 14 children participated.

Weight and Body Mass Index

Height was determined to the nearest quarter inch, and weight to the nearest tenth of a pound. We converted these measures into centimeters and kilograms for further analysis. When possible, we used the same set of universal scales for the follow-up measures as those used during the clinical trials. We used the SAS statistical package program furnished by the Centers for Disease Control (CDC) to calculate Body Mass In¬dex and Weight-For-Age standard¬ized calculations (z-scores). This program provides comparison of a child's anthropometric status to the 2000 CDC growth charts (http://www.cdc.gov/growthcha rts/). Subjects removed all outer-wear and shoes for these assess-ments. When the parents were un-able to come to the laboratory for measures, we asked school nurses or nurses within pediatric prac¬tices to obtain heights and weights. We asked nurses to use the same procedures followed in our lab (i.e., removal of outer¬wear and shoes).

In order to evaluate standard¬ized weight as an outcome mea¬sure, we compared both height and weight for all subjects with both measures. Standardized Weight-for-Age (WE-Z) and Body Mass Index (BMI-Z) (i.e., z scores) provided by the Centers for Disease Control were used.

•To determine whether changes in antipsychotic dosage were ac¬companied by changes in behavior, we , identified 13 different behavior areas. Frequency and severity data were collected as a part of the recipient's behavior man¬agement or psychiatric treatment plan.

•Behavior categories were as follows: (a) physical .aggression,-(b) property destruction, (c) verbal aggression, (d) non¬compliance, (e) self-injurious behavior, (f) psychotic behavior (e.g., hallucina¬tions), (g) mood disturbances, (h) agi¬tation, (i) running away, (j) stealing, (k) inciting, (I) inappropriate sexual be¬haviors., and (m) stripping.

•Each behavior was rated by fre¬quency and severity. Frequency ratings were assigned values as follows: 1, more than once per year; 2, more than once per month; 3, more than once per week; 4, more than once per day; or 5, more than once per hour: Severity ratings were also given numerical values: 1, mild: annoy¬ance behaviors; 2, moderate: significant pain to self or others or severely interferes 'with habilitation; 3, requires use of re- ' straint or medical intervention; or 4, ac¬tively life-threatening.

•Data from the initial 3-month period and the ending 3-month period were used to obtain Behavior Index scores in each behavior area for each recipient, using Langee's (1990) formula: Behavior Index (BI) is Severity x Frequency. These Behav¬ior Index 'worts were then summed to . obtain a single behavioral score for the initial and end period for each person. Data from 80 of the initial 95 individuals were available.

•To explore the change in antipsychotic dosage, we calculated the difference in dosage from the initial to end period' . The relation between the change score and the other variables was then determined by examining bivariate relations and, sub¬sequently, by a multiple regression

Antipsychotic dosages
Significant<br>adverse events on /following Thioridazine withdrawal

Observations on the ward and during workshop tasks

number of incidents of challenging behaviour
The participants were videotaped while being examined for dyskinetic movements using the DISCUS scale (Sprague, Kalachnik et al., 1984). One of the cameras for this study was focused on the individual's face so as to provide a clear image of lip and tongue movements with the line for recording orthogonal to the primary vertical direction of lip mo¬tion. The stereotypic lip motions recorded were naturally produced by the participant in the testing situation. To make the situa¬tion as natural as possible, we did not attach markers to the face because our previous study of tardive dyskinetic lip motions had shown that this technique provides valid and reliable kinematic lip-motion data (Sprague et al., 1996).

Each participant was tested using the motor exam that is part of the DISCUS standardized assessment procedure. The DISCUS is the only standardized TD exam and instrument developed for the assessment of dyskinesia in both psychiatric and developmental disabilities clinical populations (Kalachnik et al., 1984; Sprague, Kalachnik, et al., 1984; Sprague et al., 1989).
It is a 15-item standardized rating scale with 9 oro-bucco-lingual items and 6 head-trunk-limb items. All DISCUS testing sessions for each participant were videotaped. Three research assistants, trained in DISCUS evaluation and scoring by standardized videotapes to recognize a specified accu-racy criterion (Kalachnik et al., 1988), conducted the DISCUS evaluations for this study. We did not acquaint the research assis-tants with the medication status of the participants during the course of the study.
To provide an analysis of both changes in total dyskinesia and changes in dyskinesia at specific body points, DISCUS testing results were analyzed in terms of both total DISCUS score (sum of all 15-item scores) and individual item scores for each of the 15 DISCUS items.

Each participant was tested using the motor exam procedure that is part of the DISCUS stan-dardized assessment procedure. Dyskinesia testing was performed on a monthly basis during neuro-leptic withdrawal and then at periods of 1, 3, 6, and 12 months following complete withdrawal. The DISCUS is the only standardized tardive dys-kinesia exam and instrument developed for the assessment of dyskinesia in both psychiatric and developmentally disabled clinical populations (Sprague, Kalachnik et al., 1984; Sprague et al., 1989).
Following baseline testing, the medication dose was reduced by approximately 25% of the total dose every 3 months until complete medi¬cation withdrawal was achieved. Three points dur¬ing the withdrawal sequence were identified for each participant using the total DISCUS score ob¬tained during testing: (a) baseline dyskinesia (rep¬resenting the level of dyskinesia present prior to neuroleptic withdrawal), (b) peak dyskinesia (rep¬resenting the maximum level of dyskinesia ob¬served during the neuroleptic reduction and with¬drawal titration), and (c) follow-up dyskinesia (rep¬resenting the lowest level of dyskinesia observed following complete neuroleptic withdrawal). The results of the postural stability testing from these three time periods (baseline, peak dyskinesia, fol¬low-up) for each participant were used for analysis in the present study.
At every testing session each participant was tested on the postural stability task. This task re¬quired the participant to stand (without shoes) on the force platform with both feet a comfortable distance apart, arms

•The primary outcome was health-related quality of life as assessed by the 36-item Research and Development survey (RAND-36). The RAND-36 is a health-related quality of life questionnaire, with domains on physical well-being, role limitations caused by physical or emotional problems, social role limitations, mental well-being, vitality, pain, general health and changes in health status.
•Secondary outcomes included challenging behaviours and physical side-effects that were associated with health-related quality of life domains, according to our previous study, i.e., symptoms of parkinsonism, autonomic symptoms, irritability, lethargy and stereotypy (Ramerman et al., 2018).

Number reducing or discontinuing antipsychotic medication<br>Number of patients requiring hospital admission or change in placement
changes in antipsychotic prescribing

Number of people withdrawn from antipsychotic
Number of new prescriptions
Numbers needing extra carer support, Numbers of placement breakdown, family problems, admissions to hospital

1[illegible]
**intervention details**
•In addition to the information above, further information was available about the patients from the Leicestershire Information Co-ordination Centre (LICC). Trained interviewers employed by the centre administered a 43-page questionnaire to obtain information from carers about the person, their disabilities, their use of and needs for services, and their living environment.<br>• Following discussion with the manager of the centre, the programme of interviews was altered to ensure that all patients of the antipsychotic drug programme had been the subject of a recent interview.<br>•Of the 198 patients, agreement was reached to reduce and withdraw the drug therapy of 123. Each of the 123 patients was reviewed on a regular basis, and unless there had been any increase in the severity or frequency of challenging behaviours, a reduction of dose occurred monthly. For 43 patients, the antipsychotic drug treatment was stopped. This was usually achieved within 3 months, all achieving it within 8 months. For the remaining 80, dose reductions continued at different rates because of the emergence or worsening of challenging behaviours. For some patients, represcribing was necessary after only one reduction of dose, whereas for most, the dose had been reduced to at least half of the original before any worsening or emergence of challenging behaviours.<br>•For each patient, the review programme lasted for 12 months. Two different groupings of patients were made for analysis of outcome. With the first, patients were grouped according to the change of dose of antipsychotic drug achieved after 12 months.<br>•With the second, patients were grouped according to whether or not their behaviour had deteriorated upon drug withdrawal or dose reduction to the extent that antipsychotic dmg was either represcribed or the dose increased. The definition of the sub-groups of patients were:<br>[illegible]
•In the population reviewed, thioridazine and to a lesser extent mesoridazine were components of the behavior support plans.<br>•In this review,baseline EKGs were ordered if a previous tracing was not available on the chart.<br>•The goal was to attempt “tapering to extinction.”<br>•Cardiac monitoring addressed by the AHA Committee and cytochrome P450 enzyme system interaction concerns, particularly poly pharmacy with SSRIs and dose-related and/or metabolism variations, were reviewed.<br>•Tapering schedules were individualized for each person, and followup EKG monitoring was done 8 weeks postdiscontinuation.<br>•Prolonged discontinuation schedules were developed to avoid withdrawal dyskinesias and the decompensation/dysregulation often associated with aggressive tapering in the developmentally disabled population and to allow for stabilization with a cross-tapering agent, if needed.

Antipsychotic reduced over 14 weeks Although physicians are aware of the risks of prescribing long-term off-label antipsychotics in people with intellectual disability,attempts to discontinue<br>often fail. This study aimed to identify potential determinants of successful and failed discontinuation. Long-term off-label antipsychotics were taperedin 14 weeks, with 12.5% of baseline dose every 2 weeks.
•We asked the main support professional of the participant whether he or she was willing to provide information with regard to his or her reactions towards challenging behaviour of the participant and with regard to his or her knowledge of use of psychotropic agents.<br>•Outcome measures were collected at baseline, at 4,<br>8, 12 and 16 weeks (during the discontinuation period per protocol) and at 22, 28 and 40 weeks (follow-up) after the first dose reduction<br>•The measurements of support professional related factors took place twice, at baseline and at 16 weeks after the first dose reduction.<br>•Achievement of complete discontinuation was assessed at three time points that is 16,28 and 40 weeks after the first dose reduction, reflecting the time points shortly after discontinuation and at 3 and 6 months follow up respectively<br>follow up was 6 months after the 14 weeks over which discontinuation takes place. •Potential participants received a treatment proposal from their physician involving an attempt to discontinue antipsychotics.<br>•Discontinuation was guided by intellectual disability physicians or general practitioners according to a scheduled discontinuation time frame of 14 weeks duration.<br>•The discontinuation schedule was based on previous study, in which we found tapering off antipsychotic drugs in a relatively short time frame was done safely<br>•The study was performed as part of regular clinical care.This meant that participants remained in the study and data collection was continued to the end of the study follow-up when physicians decided the participant should no longer taper off the antipsychotic drug, should taper off in another time schedule, should use a higher dose or restart the antipsychotic drug use.<br>•To assess feelings and knowledge towards challenging behaviour and psychotropic drug use of the direct support professionals of participants, we used translated and validated versions<br>of the Challenging Behavior Self Efficacy Scale (CBSES; Hastings &<br>Brown, 2002) and the Emotional Reactions to Challenging Behavior<br>(ERCB) scale (Mitchell & Hastings, 1998)<br>We also used two<br>self-designed questionnaires with adequate psychometric properties (de Kuijper & van der Putten, 2017).<br>One questionnaire rates

•Of the total 208 patients evaluated by the BRC, test results were obtained on 54 (26%).<br>• Of these 54 patients, 27 had drug dosages decreased, 27 had drugs discontinued and none had drug dosages increased or new drugs added to their therapy. Tests were repeated within three months of the patient's last committee evaluation. A randomly selected control group consisted of 27 patients not reviewed by the BRC. Test results on the control group were also obtained within the same time pe¬riods as the study groups.<br>•The program was initiated through the creation of a spe-cial committee, entitled the Behavioral Review Committee (BRC). The time spent by the pharmacists serving in this capacity amounted to approximately one full-time equiva-lent position. In addition, senior pharmacy students par-ticipated in the program.<br>•The pharmacist reviewed the patient's chart and phar-macy records to compile a complete drug history on a special "drug calendar" form.a This form provided the pharmacist with a tool to evaluate the patient's total drug therapy at any one point in time. Thus, possible drug interactions and ad¬verse drug reactions could be identified and verified by re¬viewing the patient's progress and nursing notes. •Other pertinent information regarding the patient's current medical and psychological status was obtained, including: vital organ function, seizure history, past medical history, current diagnosis and pertinent laboratory data. After the pharmacist compiled this data base, the patient was visited to determine current problems, the effectiveness of therapy and the presence of adverse drug reactions.<br>•The above information was used by the pharmacist in preparing his recommendations to the BRC. Following the application of Weed's problem-oriented approach, as de-scribed by Kishi and Watanabe,'6 a systematic approach to drug therapy was applied, allowing a complete analysis of the case. The final step involved researching questions re-lated to drug therapy. Tentative recommendations were then prepared.<br>•The BRC consisted of a physician, pharmacist(s), nurses, psychologists, sociologists, therapists and any other person directly involved in the care of the patients.<br>•The committee met twice weekly for one and a half to two hours each time. At the completion of the BRC meeting a report was prepared by the pharmacists on each patient providing documentation of the following information:<br>(1) the patient's present drug therapy;
Introduction of interdisciplinary teams medication reviews with a goal to deprescribe antipsychotic medication typically by 25%-50% per 30 day period

In the first phase those patients who could effectively partici-pate in treatment without psychoactive medication were identified by monitoring their behavior during controlled periods of medication and nonmedication. The patients identified in phase one as drug responders participated in the second phase, in which dosage levels were systematically reduced until the minimum ef-fective dosage for each patient was found.

During both phases of assessment each individual's usual behavioral programming remained constant. A psychologist specializing in behavior analysis had de-veloped and supervised individual programs for each patient to strengthen adaptive and discourage maladap-tive behaviors. Adaptive behaviors were increased through methods such as graduated manual guidance, prompting, positive reinforcement, shaping, and mod-eling, used singly or in combination. Maladaptive be-haviors were reduced through satiation and extinction techniques and by differential reinforcement of desir-able behaviors incompatible with the maladaptive ones. More aversive procedures like time out, physical re-straint, and response-contingent presentation of aver-sive events occasionally were used when less aversive techniques proved inadequate to control maladaptive behavior.

Phase one: subjects participated in a 50-day assessment period consisting of 20 days during which they received their normal psychotropic medication followed by 30 days during which they received no medication. medication was not tapered. At the end of the 30 days of non- medication, prescriptions were discontinued for those who did not show an increase in challenging behaviours. The 50-day assessment was repeated for individuals who remained on psychotropic medication.

Phase two: 92 subjects who were unable to discontinue psychotropic were exposed to 30 days of 25% dose reduction which was repeated depending on adverse behaviours. Doses were also increased if necessary

multidiscplinary medictaion review with the aim of reducing the prescribing of psychotropic medicines

Pharmacist and PBS nurse reviewed patients with view to deprescribing in conjunction with views of patient ,carers and families

An interdisciplinary team was formed to address polypharmacy involving psychotropic medications in 3,901 community based individuals with mental retardation and developmental disabilities (MR/ DD). Individuals receiving polypharmacy were rank ordered in respect to the number of psychotropic medications taken on a daily basis. Individuals considered to be at most risk from polypharmacy were evaluated in an ambulatory setting by an interdisciplinary team consisting of a psychiatrist, neurologist, behaviorist, and psychologist<br>Three months after each clinic appointment, a telephone follow-up interview was carried out in an attempt to contact the principal caregivers for each individual evaluated in the clinic. Two follow-up questionnaires were administered during this telephone interview.<br>The first consisted of probe questions regarding: 1) whether clinic recommendations had been followed, and 2) whether the behaviors necessitating clinic referral had improved, worsened, or remained unchanged.<br>The second consisted of a 2nd administration of the ABC.
The clinical pharmacy services con-sisted of drug utilization review (reviewing the past clinical history, follow-up and evaluation of the patient's drug response, and planning alter-native drug regimens) and ordering and screen-ing appropriate laboratory tests (e.g. for lithium therapy). The new activity of the pharmacist was direct involvement with patient care by: discus-sion with a direct-care staff regarding the pa-tient's condition on the ward, observation of the patient's response (e.g. changes in target symp-toms) to the drug therapy, and minor neurologi-cal examinations i e.g. changes in motor activity and detection of extrapyramidal signs). The pharmacist visited the ward daily or weekly de-pending upon the patient's condition and the medication changes. Evaluation of the diagnostic examination by the physician to initiate the psy-chotropic medication was not included in the clinical pharmacy services. All services were pro-vided upon physicians' approval and were lim-ited so as not to directly affect other programs and plans for the patient set by the other staff<br>To eliminate subjective interpretation of the drug response by the direct-care staff and to complement the casebook, a psychotropic drug chart was devised (Inoue, 1981). The chart was prepared by the pharmacist for all patients. Brief clinical notes and a global impression of the staff were recorded both before and after medication changes. The drug therapy review periods re¬corded in the charts were between 10 and 50 months (mean 38), including 6-18 months (mean 12) retrospective evaluations.<br>All necessary information on the patient's be-havior was gathered by the pharmacist through observation of the patient as well as through communications involving many staff.<br>The final evaluation of the effect of the altered drug regimen was made six months later, al-though all patients in the facility had their drug therapy reviewed monthly.

The methodology consisted of a retrospective review of quarterly (or more frequently when available) Neurobehavio-ral Review conference (NBR) reports, generated from the beginning of 1990 until the end of 2005. These conferences were conducted on all Murdoch Center residents who required psychotropic medications for behavioral purposes. All NBR reports were analyzed by noting the initial date of symptom intensification (ie, relapse) after an antipsychotic drug withdrawal attempt7,12 and any relapses after subsequent antipsychotic drug withdrawal attempts that occurred between the time of the first relapse and 2005.<br>Relapse was defined as the occurrence during or after an antipsychotic drug withdrawal attempt of clinically significant intensified aggression including aggression toward others, self-injurious behavior, destructive/disruptive behavior, or a combination of the above behaviors requiring psychopharma¬cological intervention. By definition, relapse occurred within 3 months of the lowering or termination of antipsychotic drug administration and was reversible by an increase in dosage or reinstitution of antipsychotic medications, leading to allevia¬tion of aggressive symptoms.
We report here on a 6- to 13-year(average 10-year) follow-up of 151 institutionalized mentallyretarded adults. During the period 1990<br>–<br>1997, the subjects hadbeen prescribed antipsychotic medications to treat maladaptivebehaviors, primarily consisting of aggression,disruptive/destructive behaviors, or a combination of these. Wecompared subjects' psychotropic medication profiles in 2003 asthey related to outcome during the earlier period. Our goal wasto determine if rapid relapse (a clinically significant increasein maladaptive target symptoms, beginning 3 months or less afterantipsychotic drug termination or dosage reduction, that wasreversed by antipsychotic drug reinstitution or dosage increases)during or after routine withdrawal of an antipsychotic predictedpsychotropic drug use in 2003.

•For any review, the Committee always included either the unit manager (9% of reviews) or a direct caregiver (91%) of the client being reviewed.

• Most reviews were attended by a psychologist (89%) and clinical services coordinator (93%), and many by a pharmacist (53%) and director of programs (47%). Almost all of these personnel were from the Training Centre or the local Regional Office of the Department of Health and Community Services, though with the closure of the institution's pharmacy, a pharmacist from a community pharmacy joined the Committee toward the end of the study.

•In addition, reviews were regularly attended by a medical professional (58%), either a general practitioner from one of the community medical clinics servicing clients, or a medical practitioner with a special interest in intellectual disability medicine. Some reviews were attended by a representative of the special school attended by several of the clients involved in the study (16%), and a few by the client or other observers (5%).

• All reviews were attended by one or both of the two authors, who had the task of evaluating the operation of the Review Committee.

•Although the Committee included medical professionals, it is important to note that the considera¬tions of the Committee remained only recommendations to the client's unit manager and consulting physician. The present Committee had no authority to alter medication prescriptions. In time, it was intended that the operations of the Committee would become fully integrated with the existing system of three- to six-monthly reviews of psychotropic medication by clients' physicians.

•The principal functions of the Committee were twofold.

• The first was to develop and execute a formal procedure whereby the medication of clients with chemically restrained challenging behaviour would be maintained at a regularly ascertained minimal effective dose.

• Secondly, the interdisciplinary nature of th e committee would facilitate a team focus on a total program plan. The successful integration with medication regimens of behaviour programs and informal strategies addressing challenging behaviour could result in reduced minimal effective doses and, in some cases, elimination of psychotropic medication. These procedures for adjusting medication were designed to systematically move toward the establishment of the minimal effective dose (MED), defined as the lowest amount of medication required to control the targeted problem behaviour.

• The review process involved downward titration of the medication in small steps while closely observing

The program teas designed to make psy-chotropic drug use consistent with three principles:

that drug use could be reduced and behavior controlled with 1Ktavioral programming;

that residents would perform at least as well without such drugs;

that changes in the use of drugs could be a cooperative, interdiscipli-nary effort.

Drug evaluations were made of all residents of and admissions to the center. In many instances drug holi-days were instituted to determine the level of medica-tion needed. A few of the drug programs remained the same, but generally the process led to increases, de-creases, changes, or discontinuation of medication

•We attempted to collect fol-low-up weight and height mea-sures for all children who com-pleted some or all of the open-label trials of risperidone and who subsequently discontin-ued its use.
•We were able to make contact with the families of 14 of these children at various times fol-lowing study participation. For nearly all of the children, we had both height and weight measures at the point of study termination (discontinuation of risperidone). Thereafter, we attempted to gather follow-up measures at 3, 6, 9, 12, and 24 months after risperi-done use.
•We had both weight and height measures for subsets of the children at 3, 9, 12, and 24 months after risperidone use. However, we also had a significant number of times when only a weight measure was available. Therefore, we explored the simi-larity of standardized Weight-for-Age (WE-Z) measures as an alter-native measure that did not require height. We conducted comparisons of standardized BMI (BMI-Z) and WE-Z scores over time to determine whether stan¬dardized weight scores were suffi¬ciently similar to BMI-Z to be credible outcome measures. Thereafter, we worked with WE-Z scores. We used t tests for matched groups at the 0.05 prob¬ability level (2-tailed) for weight comparisons.

Reduction of antipsychotics associated with improvement in behaviour.
41 participants were receiving an alternative psychotropic medicine at the end of the study period, with 5 of them receiving two such drugs concurrently. The alternative drugs used were as follows: lithium (n = 26) carbamazepine (n = 9) buspirone (n = 9), and propranolol (n = 2). The prescribing of these other psychotropic medicines were associated with a reduction in the prescribing of antipsychotic medicines,

: Interdisciplinary team programme to review psychotropic medication with a view to reduce or discontinue
Retrospective case note analysis to observe effects of discontinuation

Each subject's daily ward behavior, on task, rate, and accuracy of performance in the workshop were observed on and off medication. The study included three phases for Larry, Ron, Tony, and Warren: active medication, placebo, and active medication. Building staff and observers were "blind" with respect to the presence or absence of medication. Active medication. Larry, Ron, Tony, and Warren were observed for 19 days to evaluate ward behavior while they were taking CPZ. All subjects were observed after their lunch, 5 days a week (Monday through Friday), between 12:30 and 1:30 p.m. There is some variation among subjects as to the number of daily observations in each condition due to physical problems, e.g., subject being absent due to physician's appointment, observer being absent, and so on. Data were collected on the workshop tasks during two daily 10-min sessions held between 1:30 and 2:30 pm.
Placebo. For the next 23 days, a placebo was substituted for active CPZ. Observations of ward and workshop behavior continued.
Active medication. For the final 25 days, CPZ was reinstated at the same level as employed in Phase 1. Observations of ward and workshop behavior continued.
The first two experimental conditions were identical for the fifth subject, Charles. During the third condition (reinstatement of CPZ), however, Charles was not given an active medication for 1 day. Moreover, he was removed from active medication midway through the final drug condition for clinical reasons.
1. The four subjects who received 200 mg of CPZ daily received one 100-mg tablet at 9:00 a.m. and another at 9:00 p.m. The fifth subject, Larry, received two 100-mg tablets at 9:00 a.m., 1:00 p.m., 5:00 p.m., and 9:00 p.m. The tablets and water were administered by the nursing staff.The nurse routinely remained with each subject until they had swallowed the tablet(s) and water.

During placebo condition(s), all subjects were abruptly withdrawn from CPZ. A visually identical, nonactive placebo supplied by Smith, Kline, and French Laboratories was substituted.Administration procedures were identical to those during the active medication conditions.
"Double-blind" conditions were maintained throughout the study. Prior to the study, the prescribing physician coded three identical pharmaceutical bottles for each of the five subjects by labeling them with the subjects' names and specific dates of administration.
Two of the bottles contained active CPZ, and the third contained the placebo.
A sufficient number of tablets was provided for each subject to last the number of days of the respective

Thioridazine was usually tapered at a rate of 10% of the original dose every 3 months, thus it took most patients 2 to 3 years to be removed completely (see Figs. 1-3). Data were collected for at least 1 year after thioridazine was discontinued. In all cases data were recorded by direct care staff and evaluated by a psychologist (RT).

Each participant was tested using the motor exam that is part of the DIS-CUS standardized assessment procedure. Participants were tested during baseline (while on their maintenance dose of neuroleptic medication) and then at least monthly during the course of their neuroleptic withdrawal schedule.
Follow¬ing baseline testing, the medication dose was reduced by approximately 25% of the total dose every 3 months until complete medication withdrawal was achieved. Test¬ing continued for each participant for a postwithdrawal follow-up period that lasted between 6 months and 2 years across participants.
Three points during the withdrawal sequence were identified for each par-ticipant using the total DISCUS score obtained during testing: (a) baseline dys-kinesia (representing the level of dyski-nesia present prior to neuroleptic withdrawal), (b) peak dyskinesia (repre-senting the maximum level of dyskinesia observed during the neuroleptic reduc-tion and withdrawal titration), and (c) follow-up dyskinesia (representing the lowest level of dyskinesia observed fol-lowing complete neuroleptic with-drawal).

Testing was done in the context of an ongoing quarterly dyski-nesia monitoring protocol that was part of the routine clinical monitoring procedure in place at the facility for persons who were receiving neuroleptic treatment. In the case of the participants referred for this study, the frequency of monitoring was increased to a monthly schedule to provide monitoring of potential with-drawal-emergent effects. Participants were tested once during baseline (while on their maintenance dose of neuroleptic medica-tion), then monthly during the course of their neuroleptic with-drawal schedule, and then at intervals of 1, 2, 3, 6, and 12 months following complete medication withdrawal. Treating physicians and treatment teams for these participants followed the general neuroleptic dose reduction protocol in place at the facility. Ac-cording to this protocol, and following initial baseline testing, the dose of each participant's neuroleptic medication was reduced by approximately 25% of the total dose every 2-4 months until complete medication withdrawal was achieved. In some cases, regularly scheduled medication reductions were postponed for 4 months because of apparent withdrawal-emergent behavioral tox-icity (mood lability, increased activity level, irritability, sleep disturbance, or aggression). The decision to postpone a partici-pant's scheduled dose reduction was made by the participant's treatment team and not by research personnel.
The effect of neuroleptic withdrawal on postural task performance of 20 adults with mental retardation was examined. Dyskinesia was measured using the DISCUS rating scale and postural stability using a force platform during a prospective longitudinal neuroleptic med-ication withdrawal protocol.

•In both trials discontinuation was scheduled as a 12.5% reduction every two weeks of the baseline antipsychotic drug dose.

•In the double-blind trial, participants were prescribed the fluid form of risperidone (1mg/ml) to enable stepwise, placebo-controlled discontinuation. Every participant received two bottles of medication. In the discontinuation group, the first bottle contained risperidone and the second bottle placebo. During the 14-week withdrawal phase, the amount taken from the first bottle lowered with 12.5%, while the amount taken from the placebo increased with 12.5% every two weeks.

•In both trials, participants were allowed to stop the withdrawal of antipsychotic drugs at any time, to taper off more slowly, or restart antipsychotic drugs after complete discontinuation. The decision to stop the scheduled discontinuation or the restart of the antipsychotic drugs was the responsibility of the participant's physician.

• Reasons to stop the scheduled discontinuation could be an increase in challenging behaviours, the emergence of psychotic symptoms, or withdrawal symptoms.

Step1: Primary care and identification of the cohort
local GPs invited to attend a STOMP- related one-hour tutorial in which the present authors described the STOMP initiative and our proposal to withdraw antipsychotic medication.
Involving GPs at the outset made it easy for us to access GP reg- ister-based database. The present authors have used Read codes (a coded thesaurus of clinical terms) to identify adults with intellectual disabilities who are on antipsychotic medication in the area. The pres- ent authors also used a software program called ECLIPSE (Electronic Checking Leading to Improved Prescribing Safety and Efficiency) to identify all people on the Learning Disability Quality Outcome Framework (QoF) register on antipsychotics who did not have a re- corded mental disorder in 44 practices in Cornwall. The present authors also carried out an audit of people with intel- lectual disabilities on antipsychotics who were discharged back to pri- mary care from the secondary care between 2010 and 2015 to assess in what proportion of cases an annual health check (AHC) involved a satisfactory antipsychotic medication review
Step 2: Involving all stakeholders
The present authors organized a meeting where the people with intel- lectual disabilities and their carers were invited, and discussed all the issues involved in the withdrawal process including risks and benefits- i.e. changes in diagnostic systems over time, exposure of an undiagnosed mental illness, uncover unsuitable placement, risk of hospital admission
patients and their carers and other stakeholders remained totally involved in the withdrawal and relapse prevention strategy.
Step 3: Secondary care
Within the secondary care, the present authors involved CLDT mem- bers regularly to discuss the strategy for withdrawal.
The multi-disci- plinary team (MDT) was also involved in the discussion of individual cases when withdrawal was considered.
However, the present authors integrated primary and secondary care within a whole system ap- proach by establishing a STOMP oversight committee (project team). This committee was led by the clinical director of the local special- ist Learning Disability service with multiagency representation from service users, primary care liaison nurses, community pharmacists, commissioners, social care and private providers with regular meet- ings to discuss ideas and review progress. After multiagency consulta- tion, and receiving

Medication reduction trials

119 adults with intellectual disabilities wereincluded in a programme of antipsychotic drug withdrawal followi

2 months
**intervention duration**
mostly over 3 months
variable

14 weeks

variable

variable

30 days for phase 1, Variable for phase 2

variable

variable

variable

variable

variable

sudden discontinuation

variable

sudden discontinuation for 23 days

variable

variable
variable

14 weeks

variable

Intervention follow up
12 months
8 weeks post discontinuation

6 months following planned discontinuation

variable

nearly 2 years

variable

variable

3 months
variable

up to 15 years
10 years

2 years

24 months

variable

48 days

3 to 4years
6 months to 2 years post discontinuation

12 months post discontinuation
12 months post discontinuation

6 months post planned discontinuation

3 months
12 months post discontinuation

variable

Outcomes

•18 persons were identified and 15 were successfully discontinued. One person was stable on therapy with EKG monitoring within the recommended guidelines; complete initial baseline information was unavailable for two individuals

•Of particular interest to the team were the individual changes, particularly the increases in QTc prolongation times in five patients after discontinuation of medication. All received thioridazine and all were male .

•Even with the increased QTc prolongation interval, two persons' intervals remained less than 400 msec. Another individual was on the cutoff,and the interval increased only slightly.

•The remaining two individuals experienced more marked increases (408 vs. 448 and 401 vs. 456 msec). These may be related to individual cardiac function variations or other medication-related effects.

•These individuals are scheduled for 6-month follow-ups.

In this open-label discontinuation study, 61% of participants were able to discontinue off -label antipsychotics completely in 16 weeks. At follow-up 3 months later (28 weeks after the first dose reduction) 46% of participants were still completely off their antipsychotic medication; at 40 weeks' follow-up, 40% were still completely discontinued. These results are in line with other discontinuation studies7 but somewhat better than those seen in our previous study, in which 43% achieved complete discontinuation and 36% were still off antipsychotic medication 3 months later. Results with regard to the course of neurologic side effects were mixed: at 40 weeks, there was a decrease in parkinsonism in participants who were completely discontinued and a decrease in akathisia in participants who were incompletely discontinued. The decrease in akathisia in participants who were still on antipsychotic drugs may be explained by the potential of these agents to mask this extrapyramidal symptom or by the disappearance of withdrawal akathisia.13 Autonomic symptoms increased in participants who were incompletely discontinued at 16 weeks. This may have been the result of an increase in the dosage or additional prescriptions of psychotropic drugs intended to treat the behavioral disturbances that led to termination of the discontinuation trajectory.

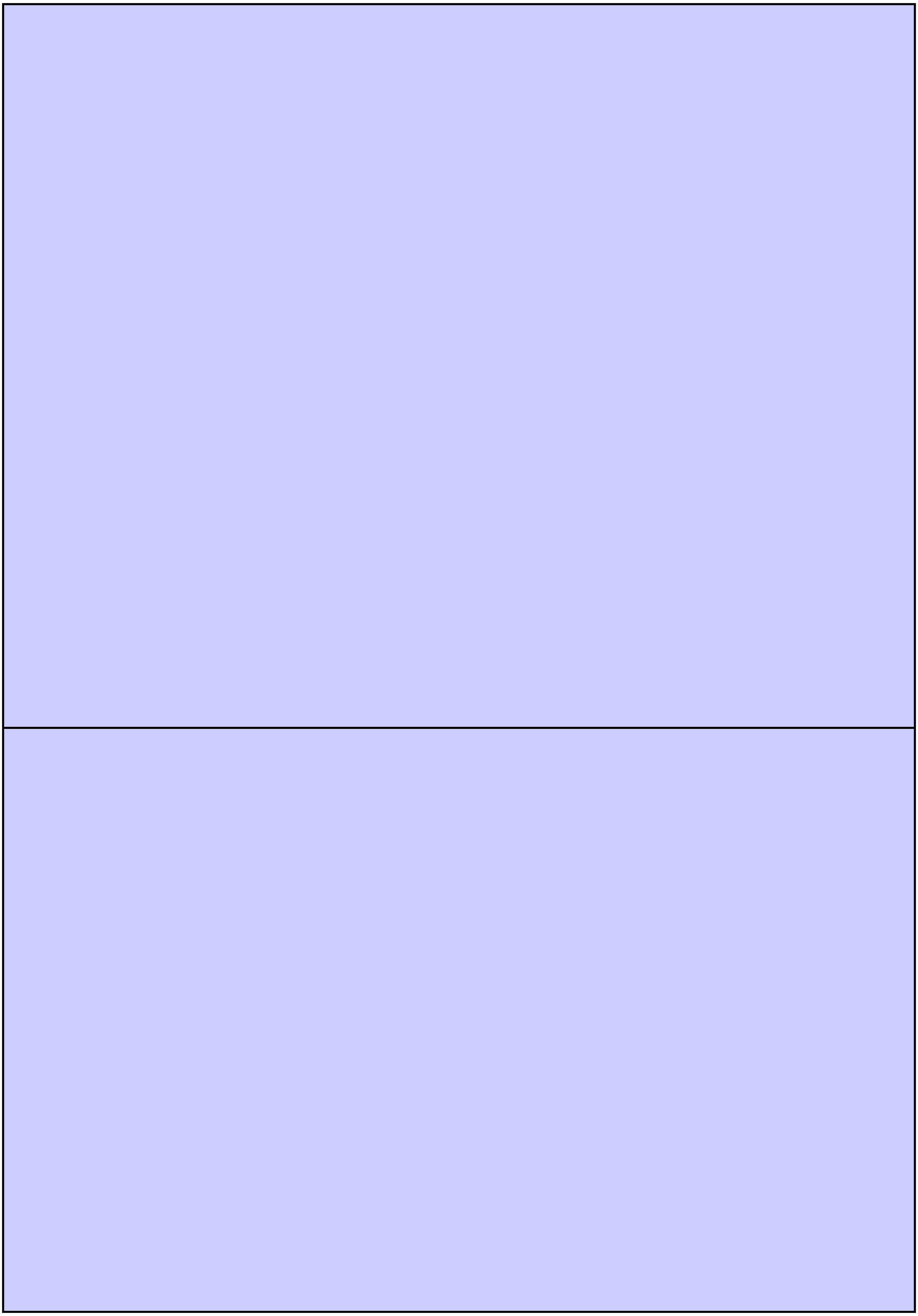

While the major benefit of decreased reliance on psy-choactive medication accrues to the individual resident, there also are benefits for the institution. A direct dollar savings results from decreased expenditures for drugs. Indirect savings result from the decrease in staff time needed to set up, administer, and record medications. To date, the hospital's drug assessment program has produced an annual direct dollar savings of more than $16,000

There also has been an annual reduction of more than 33,000 hours in medication-related staff activity. In ad¬dition, little or no extra staff time was needed to imple¬ment the drug reduction program since the required pa¬tient observations and recordkeeping had to be done anyway for regular behavioral programming. Both the direct dollar and staff-time savings have helped the in¬stitution better meet the needs of its retarded residents.

Due to the efforts of the Behavior and Medication Review Committee, the Wyoming state Training School has experienced a signifi-cant decline in usage of psychotropic medica-tions
This reduction in medication usage was obviously accompanied by a significant cost savings.

By the end of the five year period, 135 psy-chotropic medication orders for 121 patients were discontinued. The dosage reductions (25-75%; mean 48.6%) were made for 91 medi-cation orders. Many patients (50.4%) were re-ported to show significant improvements in cog-nitive functions as observed by the direct-care and nursing staff following these medication changes, though no objective measurement nor control group studies were possible because of a limited number of trained staff. No change and improved were a result of a dosage reduction and discontinuation

forty-nine of the 57 individuals who had experienced an initial antipsychotic drug withdrawal–related relapse had a subsequent antipsychotic medication withdrawal attempted. Fourteen (28.6%) of these 49 subjects experienced 1, 19 (38.7%) experienced 2, 10 (20.4%) experienced 3, and 4 (8.2%) experienced 4 additional relapses; 2 (4.1%) did not relapse. The mean number of relapses per individual was 2.0. The remaining 8 individuals had been kept on first-generation antipsy-chotic agents or were directly transitioned to second-generation antipsychotic agents and had not had subsequent withdrawal attempts after the initial relapse. The lack of further withdrawal attempts was usually due to the danger-ousness of the first relapse and the related fear of future relapses.<br>Our results indicate that for individuals who have experienced intensification of maladaptive symptoms during withdrawal from a first-generation antipsychotic medication, all but 2 had a subsequent relapse or relapses when further attempts at antipsychotic medication withdrawal occurred, and only a small minority eventually became antipsychotic drug free. In these individuals, there was often a cycle of aggression during each withdrawal attempt, with some of these destructive episodes causing significant harm to self or to others. Symptoms decreased when antipsychotic agents were either increased in dosage or reinstituted.

•Prior to the introduction of the Committee, medication review took place on 3 to 6 month cycles and essentially involved the client's physician and unit manager (or representative).
•The Committee involved a much larger group and met regularly, and hence was quite labour intensive. Consideration of each case was thorough. In 47 meetings of up to two hours duration, 336 reviews were conducted. This represents an average review allocation of 17 minutes. It could be argued that it is slow progress to effectively deal, in two years, with only 25 out of 69 clients at the Training Centre on chemical restraint. The fact remains, however, that substantial reductions were achieved with these clients, and for most of the continuing clients, further reductions were yet possible.
• A further consideration is that review durations decreased as the Committee gained proficiency. Whereas, in the first year, 136 reviews were conducted (averaging 5.7 reviews per meeting at 21 minutes each), 200 reviews were completed in the latter year (8.7 reviews per meeting at 14 minutes each). In time, the ongoing operation of the Committee will review all clients. With many clients coming off medication altogether, it can be anticipated that the Committee may be able to maintain regular monitoring of those clients still on medication.
•In the present study, the three clients whose medication was ceased altogether arrived at the MED of zero within 5 to 7 reviews.
• For other clients, however, particularly those whose behaviours were more frequent or more serious, the review process became a protracted procedure.
•In one case, for example, a client had been reviewed 26 times to achieve a medication reduction of 16.7%. The Committee was very cautious to recommend medication titration on the one hand, but very reluctant to remove the client from the review process on the other. As time went on, the Committee's investment in the client's participation grew, and with it, reluctance to discontinue the client's reviews. Had the Committee set a review-outcome criterion, whereby a case would be set aside for a period were a pre-determined progress rate not met, it is conceivable that several more clients than was the case would have had the opportunity to benefit by participating in the review program in the first two-year period.

•Di-rect-care staff were extremely responsive to the ap-proach and were encouraged by some residents' in-creased participation in programming.
•The process initially caused anxiety among some of the staff becau.:e the center lacks seclusion rooms or other locked facili-tie, and physical and mechanical restraints were rarely used.
•Some of the residents exhibited behavior prob.-lems upon withdraw;, •-f the drugs, but they were care-fully monitored until re-stabilization took place
•Between implementation of the program in July 1974 and January. 1978, the percentage of residents on medi-cation declined from 34 per cent to 21 per cent. The percentage of residents on major tranquilinrs, thc most frequently prescribed drugs, dropped by 7 per sent. from 27 per cent to 20 per cent. Five per cent of the residents were receiving minor tranquilizers in July 1974; by January 1978 their use had been eliminated.
•The study also revealed some interesting fiadings about drug use patterns among different disability groups. In one cottage for profoundly retarded persons with serious emotional or behavior problems. 25 per cent of the residents were receiving major tranquilizers and 5 per cent were receiving minor tranquilirsrs at the beginning of the program. By January 1978 only 5 per cent were receiving major tranquilizers. and the use of

For 8 particpants weight gain was the reason given by par-ents for discontinuation, while for 2 subjects, the parents reported only increased appetite. The par-ents of 5 children reported insuf-ficient response (3 of whom also had excess weight gain). The par-ents of 2 children did not directly report these types of factors, though weight gain was present. Nine of the 14 subjects used per-mitted concomitant stimulant med-ication during the clinical trials. We know that 6 of the 9 children who used stimulants continued to do so after the discontinuation of risperi-done, but we were unable to ob-tain this information from the other 3. We know that of the 5 who did not use concomitant stimulants, 1 did begin use of stimulants after discontinuation of risperidone.

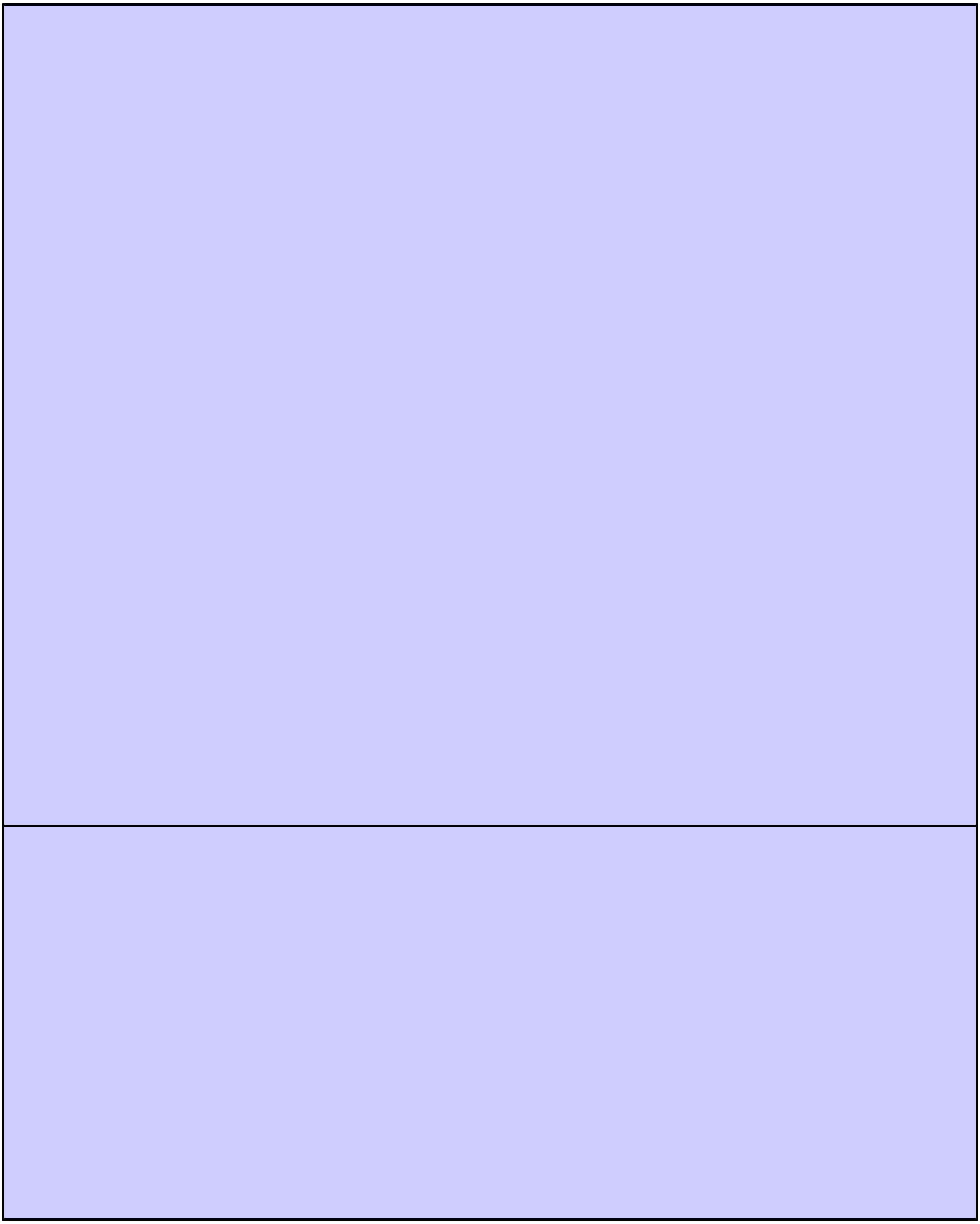

Overall thioridazine was reduced from a total congregate starting dose of 8750 mg/day in 1989 to 0 mg/day by 1992. The total frequency of destructive behaviors rose from a baseline established in 1989 of 882 incidents per month average to 1113 per month average in 1990 and then decreased to 746 average per month during 1993. Coincident with this rise and subsequent fall in destructive behaviors, the use of mechanical restraints (as an indirect measure of severity) increased from an average of 25 times/month to 91 per month and then decreased to an average of 18 times/month during 1993.<br>Individual patterns of behavior fell into three groups. There were no significant differences of age or IQ between the three groups. Group 1, which con- sisted of nine individuals, demonstrated a transient worsening and then return to baseline (Fig. 1). Group 2, which consisted of five individuals, demon¬strated a progressive decrease in maladaptive behav¬iors (Fig. 2). Group 3, which consisted of nine individuals, demonstrated persistent worsening of be¬havior that lasted for more than 2 years (Fig. 3). De¬tailed tables which provide specific demographic frequencies of target behaviors and doses of thiori-dazine are available from the authors on request.<br>The study reported here suggests that reintro-duction of the drug may not always be necessary since behavioral deterioration seen in 78% (18/23; Groups 1 and 3) was transitory half the time.Transient behavior problems may resemble psychiat-<br>ric syndromes, thus making it difficult to ascertain whether they represent a "withdrawal" state or a bona fide psychiatric disorder. The temporary use of "alternatives" (redirection, environmental changes, the use of carbamazapine, valproic acid, pro-pranaolol, clonidine, etc.) thus might provide the possibility for symptoms to resolve without chronic pharmacological intervention.
This kinematic analysis of lip mo-tions in tardive dyskinesia as a function of medication withdrawal shows that there is an inverse relation between the amount and the complexity of organiza-tion of the motion. Specifically, greater lip movement, produced by neuroleptic withdrawal and identified clinically as dyskinesia, is associated with a signifi-cant decrease in the complexity of the sequential pattern of lip movement. The data confirm the general proposition that the enhanced movement variability asso¬ciated with tardive dyskinesia is related to a reduction in the dfs that are organiz¬ing the movement dynamics (Newell, 1996). Thus, the prolonged regimen of neuroleptics leads to enhanced constraint on the organization of the motor system, and this in turn leads to enhanced move¬ment variability as observed in behavior.<br>The amplitude of lip motion showed the well-known pattern of greater ampli¬tude and variability of amplitude on drug withdrawal. This increment of motion on drug withdrawal can often be detected by natural observa¬tion and rating scale The present findings show that the frequency of the lip oscillations changed over the drug manipulation conditions, with the peak withdrawal dyskinesia con¬dition producing a longer period (slower frequency) of oscillation than was appar¬ent at either baseline or long-term follow-up. The kinematic analysis also revealed that the lower lip had the greater ampli¬tude of lip motion, a finding that reflects the anatomical structure of the mouth and the greater motion that can be pro¬duced by the jaw and, hence, lower lip.

The results of this study showed the expected general increment in dyskinesia due to medication withdrawal. The mean level of effector activity at peak withdrawal was significantly higher than at both baseline and follow-up, and this trend was present for all individuals. There was no difference in the mean level of TD at baseline and follow- up, showing that the average level of TD that was increased on peak withdrawal was eventually reduced to the baseline level. The mean total DISCUS scores at baseline and fol¬low-up, although lower than the cutoff standard adopted in the DISCUS test (cf. Sprague et al., 1989), nevertheless indicate that TD was present on average at both of these testing periods.
There was a substantially different effector pattern at peak withdrawal to that evident at baseline and follow-up testing. Significantly more items contributed to the total dyskinesia score at the peak withdrawal time period than at the baseline or follow-up time periods, suggesting that the effects of dyskinesia had spread throughout the effector areas.
Our findings indicate that as TD progresses there is a pattern of increased involvement of separate body areas. Thus, with progressing TD, body areas formerly free of signs of involuntary movements come to display involun-tary dyskinetic movements. This finding is consistent with the notion that dyskinesia is associated with a global de-crease in voluntary muscle control and is in contrast to models that hold that dyskinesia is primarily a focal disorder (e.g., of the oro-facial region).

The findings from this study of adults diag-nosed with mental retardation show that neuro-leptic withdrawal influences both the overall level of tardive dyskinesia and the postural stability of whole body standing postural. The general level of tardive dyskinesia as indexed by the DISCUS (Sprague, Lachnik et al., 1984; Sprague, White et al., 1984) was significantly higher at peak with-drawal than at baseline and follow-up. The medication withdrawal protocol con-ducted in this study allowed us to examine the dynamics of standing posture over the progres¬sive range of medication withdrawal. Here we have selected, on an individual subject basis, the peak withdrawal dyskinesia and the final point of follow-up after complete medication withdrawal as points of comparison to baseline conditions of tardive dyskinesia. The trend to reduce the ab¬solute amount of postural motion at peak with¬drawal was achieved through apparent stiffening of the body musculature, as the regularity of the postural motion was also increased under this condition. However, it is important to note that the absolute level of postural motion in all three medication conditions is still very high in con¬trast to that of the postural control of healthy individuals (cf. Ko et al., 1992; Newell, van Em-merik, Lee, & Sprague, 1993). The reduction of motion on medication withdrawal arises from the further reduction of the active degrees of free¬dom of the system around what in essence is a limit cycle form of dynamical organization to the center of pressure. In contrast, the onset of dys-kinesia is indexed by an increase in motion aris¬ing from the change of point attractor dynamics to those of a limit cycle (Newell, 1996). This pat¬tern of change is consistent with findings from our previous studies of the motor control of

•This study is the first to study the effects of discontinuation of antipsychotic drugs on health-related quality of life, in people with intellectual disability. However, there are also some limitations.
•First, we used the RAND-36 as a measure of health-related quality of life, a scale widely used and validated, but not for the use in people with intellectual disabilities or for completion by proxies. Validation of this scale for people with intellectual disabilities (and their proxies) may support future research on this subject.
•Second, we combined two studies with a different design. Although the schedule of discontinuation was the same, there might be a difference in results between blind discontinuation and open discontinuation. It can be expected that participants in the double-blind study are more inclined to continue with discontinuation, even when discontinuation becomes more difficult, by for example an increase in challenging behaviour.
•Moreover, there may be a difference in bias between the two trials on the completion of the RAND-36 and other scales, by the caregivers. In the open-trial, the caregivers were not blinded and all participants were discontinuing antipsychotic drugs. In contrast, in the double-blind trial, caregivers were blinded on the discontinuation of their client when completing the questionnaires at baseline and week 16.
• This may have affected the answers provided on the scales.
• Last, there was no control group to compare the normal fluctuation in health-related quality of life with.

7.6% completely withdrew from antipsychotic medicines, and 48.7% experienced onset/deterioration in problem behaviours or mental ill-health.
The cost to the intellectual disabilities psychiatric service (over and above that of routine psychiatric care) was £258,050.
Ten people required increased levels of carer support to be provided; seven were excluded from a day centre placement, one person experienced a placement breakdown and moved to a new home, and six experienced considerable family problems.
Fourteen hospital admissions to an intellectual disabilities psychiatric assessment and treatment unit

Key findings of study
•The type of accommodation did not appear to be associated with the successful withdrawal of antipsychotic drug therapy.<br>A number of possible variables could have affected this outcome.<br>On wards and in hostels, staff have more support from nearby colleagues, whereas staff working in the community may be more isolated and feel more vulnerable.<br>•This could result in the community-based staff feeling more reluctant to agree to withdrawal of the drug or to be prepared to tolerate challenging behaviours.<br>In community settings, challenging behaviours may be less acceptable than in hospital or hostel settings. Once a person has been placed in the community, there may be great reluctance to do anything which may jeopardize that placement.<br>• In the ward setting, the behaviours may be more potentially dangerous or severe. Although there was a greater reluctance to reduce the antipsychotic drug therapy in community settings, the outcome was similar for those who underwent reduction or withdrawal. This would support the hypothesis that the outcome was more related to the reduction in drug therapy and its effects on the patient than the type of accommodation.<br>The successful withdrawal of antipsychotic drug treatment (3 I patients) was associated with:<br>•lower doses,<br>•predominantly of the drug thioridazine,<br>•minimal psychopathology,<br>•the presence of epilepsy,<br>•and the lack of hyperactivity, aggression or stereotypy.
15 participants discontinued Thioridazine increases in QTc prolongation<br>times in five male patients after discontinuation of Thioridazine, three patients slight increases and two patients more marked increases.

The present study indicated some determinants that might help predict whether discontinuation will be successful.

• The presence of autism, akathisia, higher ABC scores, and more frequent worsening in health during discontinuation were clearly associated with lower odds of successful discontinuation. In people with intellectual disability, comorbid mental disorders, neurologic side effects of antipsychotics, and ill-health conditions may express as behavioral symptoms, which may be difficult to manage.

•When underlying causes of maladaptive behaviors are not recognized, appropriate treatments will be lacking, and the severity of behavioral symptoms may increase, which in turn may hinder successful discontinuation.

•The commonly accepted idea that change in environmental circumstances should be a reason not to start or even to stop antipsychotic discontinuation trajectories could not be confirmed, given no association was seen in this study between change in living circumstances or life events and lower incidence of successful discontinuation.

• We identified a number of determinants potentially capable of explaining failure to achieve complete discontinuation that have clear clinical implications. In particular, worsening of chronic medical conditions or temporary ill-health conditions in clients, which were negatively associated with successful discontinuation, should be addressed appropriately but should not necessarily be a reason to terminate a discontinuation trajectory.

• Also, the presence of neurologic side effects should be carefully examined and appropriately managed; in this study, such symptoms were associated with higher severity of maladaptive behaviors, which in turn were associated with a higher incidence of failed discontinuation. Finally, the presence of autism spectrum

•Of 499 eligible patients,129 were recruited. reasons for non participation: •Of the 129 participants, 61% achieved discontinuation at 16 weeks; at 28 and 40 weeks, 46% and 40% were completely discontinued.

•Staff's feelings of Depression/Anger towards their client's behaviour, less knowledge about psychotropic medication and clinicians' judgements of behavioural worsening were negatively associated with achievement of discontinuation.

•We indeed found significant differences in staff-related variables between those participants who succeeded and those who
failed in complete discontinuation. Also, we found a number of staff-related factors that were associated with the chance of
complete discontinuation.

•Male gender and feelings of depression/anger of support professionals, and clinicians' judgements of worsening in behaviour during the discontinuation trajectory were associated with a lesser chance of complete discontinuation, and clinicians' judgements of no worsening in behaviour with a higher clinicians' judgements of no worsening in behaviour with a higher
likelihood.

•Furthermore, we found support professionals' education and knowledge of psychotropic drug use, and agreement in
staff with regard to participants' eligibility to discontinue the antipsychotic drug use were positively associated with successful
discontinuation.

•Remarkable findings were the association of support professionals' feelings of "cheerful/excited" and "confident/
relaxed" with failure and the association of clinicians' judgement of higher baseline severity of maladaptive behaviour with success in discontinuation at 16 weeks.

During the two-year study period, the use of antipsychotic drugs was reduced 18%; antianx-iety-antidepressant drugs, 50%; sedative-hypnotics, 58%; and miscellaneous agents, 65%. The net savings in drug expenditures was more than $10,000per year. Maladaptive behaviorial test results implied that the decreased drug use did not change behavior significantly.
•Of the 129 patients 61% had comopletely discontinued APs at 16 weeks, 46% at 28 weeks and 40% at 40 weeks.

•ABC scores increased in 49% of patients with unsuccessful discontinuation at 16 weeks

•Autism, higher dose of AP, higher ABC, and akathisia scoresand more frequentl worsening of of health during discontinuation were assocoaited with lower incidence of discontinuation

•In total, 66 people have received a STOMP challenge, with 24 psychotropic medications being stopped; 20 of these were with PBS support. A further 22 people are undergoing the challenge which is not yet complete. Ten medications needed to be restarted post-discontinuation or increased post-reduction, with eight being in the unsupported clinic. On average, each person required a minimum of five reviews to fully undertake the challenge.

•The majority of medications stopped are antipsychotics, as reflected in NICE guidance, which only supports the use of antipsychotics for behavioural management. Over half these prescriptions were for risperidone, which reflects the clinical practice that this antipsychotic was the preferred choice in behavioural intervention.

•Side effect burden reduced by 71% with a reduction of 50% of the starting dose or more. The main issues that improved were sedation, weight gain and postural hypotension. Many of the prescriptions had historically been for risperidone, and improvement in hyperprolactinaemia, often risperidone-induced.

The following recommendations were made by the interdisciplinary team:<br>1) decrease in the number of psychotropic medications in 43% of the patients;<br>2) shift from classical to atypical antipsychotic preparations with the most marked reductions in the usage of thioridazine, haloperidol, and chlorpromazine;<br>3) reduction in the usage of anticholinergic agents, lithium and anticonvulsants when the latter were being used for behavioral disorder; and<br>4) reduction of tricyclic antidepressants in favor of selective serotonin uptake inhibitors.<br>Follow-up of compliance with recommendations and outcome of medication change were carried out through the use of two informant questionnaires given to the principle caretakers.<br>When recommendations were followed, 73% of principle caretakers reported an improvement in the behavior prompting original clinic referral whereas only 19% showed improvement when recommendations were not followed.<br>The most frequent medication change recommended was a shift from typical antipsychotic medications to atypical antipsychotics. In the population reported in this study, thioridazine use was reduced by 63%, haloperidol by 72%, and chlorpromazine by 100%.<br>In reducing classical antipsychotic medications, the IDT recommended discontinuing anticholinergic medication in 21 patients.<br>Other recommendations included reducing the use of tricyclic antidepressants in favor of selective serotonin uptake inhibitors. These changes were prompted by side effects of the tricyclic medications including excessive weight gain, sedation, dry mouth, and constipation.<br>Following evaluation by the IDT, lithium was discontinued in 18 patients because of lack of evidence of a bipolar disorder, no convincing evidence of efficacy for the targeted behaviors, and occasional occurrence of secondary hypothyroidism. In 19 subjects, benzodiazepines were recommended for discontinuation because of lack of response or concern about side effects
Following five years of the drug utilization review process by the pharmacist, the result is a significant reduction of psychotropic drug use. The reasons for the use of excess drugs are not clear; however, a long-term systematic review and a coordinated objective evaluation of the patients' responses to drug therapies were missing prior to the implementation of the clinical pharmacy services

For those individuals successfullywithdrawn from antipsychotic medications, 66.3% (55/83) werestill psychotropic drug free in 2003. For those who rapidlyrelapsed during the period 1990
1997 following antipsychotic drugwithdrawal or dosage decreases, only 9.0% (5/55) werepsychotropic medication free in 2003.

•For 25 clients receiving psychotropic medication in the management of challenging behaviour, daily doses of neuroleptic medication were reduced by an average of 43.9 %.<br>•Across all psychotropic drugs administered as chemical restraint, daily doses were reduced by an average of 51.2%.<br>•Polyp harm acy prevalence was reduced from 52.0% to 24.0%.<br>•Three of the clients were totally removed from all psychotropic medication with no deleterious outcome. None of the 25 clients had a net medication increase.
Patients prescribed psychotropic medication decreased from 34% to 21%, percentage prescribed major tranquillisers fell from 27% to 20% and minor tranquilisers were no longer used having accounted for 5% of patients.

Analysis of standardized weight scores in relation to standardized BMI scores suggested marked similarity between them at all time points. Comparison of standardized weights at time of drug termination with 3, 9–12, and 24 months after termination indicated that weight gain during risperidone treatment is reversible (i.e., significantly less weight after risperidone was discontinued) at all time points after termination. Furthermore, standardized weight at 12 and 24 months after discontinuation of risperidone was not distinguishable from standardized weight before risperidone. The prospect of reversibility may provide some comfort for clinicians and parents alike, but far more data are needed before an assumption can be made that this is the case for all children.

•Presence of a psychotic diagnosis was a significant variable in increased antipsychotic dosage. Use of alternative medication (carbamazepine, buspirone, lithium, and propranolol) was related to decreased antipsychotic dosage.

•Findings suggest that individuals with mental retardation who do not have psychoses are a suitable group for reduction and that use of alternative medications facilitate this process for individuals with or without psychosis.

Antipsychotic dosages decreased at a projected annual rate of 17% and no significant withdrawal reactions reported. This dosage decrease has saved the Institution approximately $2800 to $3200 in medication costs after a 10-month period.
Over 50% of those on regular thioridazine experienced adverse events during or following drug withdrawal. Adverse events were significantly associated with the duration of previous thioridazine prescription. Problems encountered included re-emergence of psychosis or mood disturbance, escalation of arousal, aggression, anxiety, self-injury, sexual disinhibition, and ritualised behaviours. Further details of adverse effects not reported.

The behavior of these five retarded adults was heavily influenced by CPZ withdrawal in some cases and virtually unaffected in others. The magnitude of the rate changes as well as the behaviors affected varied among subjects in the unstructured residential setting. In contrast, only one of four subjects was consistently affected by CPZ withdrawal and reinstatement in the structured workshop setting.

•In a naturalistic study of 23 severely and profoundly mentally retarded adult male patients undergoing slow "diagnostic" neuroleptic taper, it was determined that at least 60% could eventually be managed without psychoactive medication.<br>•However, many of these demon-strated a remarkably long, but nonetheless transient, period of worsening.<br>• This suggests that behavioral deterioration during drug reduction trials does not always indicate a need for chronic neuroleptic maintenance since these behaviors may return to baseline without phar-macological intervention.<br>•On the other hand, 40% demonstrated persistent (>2 years) be-havioral worsening.<br>•Those individuals who demonstrated persistent deterioration had been generally well controlled on neuroleptics, were somewhat older, and were receiving a higher baseline dose.<br>•Most of these persistently worsened subjects currently require some type of psychoactive medication (although only two have been returned to neuroleptics).
Results showed that the changes in the amount of lip oscillations following medication reduction and eventual withdrawal were strongly linked to changes in the structural complexity of the dynamics of lip motions. These findings provide evidence that neurolep-tic medication reduces the df of the dynamics of the movement output and that this change is inversely related to the level of tardive dyskinetic motion observed in the clinical setting.

Results indicated that the effector pattern of TD changed over the course of neuroleptic withdrawal. Peak dyskinesia was associated with the involvement of more body areas relative to baseline. Although dyskinesia decreased at follow-up and fewer body areas showed signs of dyskine-sia, there were still differences in the effector pattern of dyskinesia relative to baseline at periods of 1 to 2 years following neuroleptic withdrawal.
Finally, it is noteworthy that there was not a return on average to typical healthy postural cen-ter of pressure profiles even after one year of the medication reduction and withdrawal program. Tardive dyskinesia as indexed by postural task performance was still present at the end of the medication withdrawal program in about 33% of the subjects, even though observable manifesta-tions of dyskinesia (e.g., DISCUS scores) had de-creased significantly and, in many cases, below the accepted cut-off for determining dyskinesia.<br>Moreover, the overall level of postural motion at follow-up was considerably higher than that typ-ically observed in healthy age-matched controls (Newell, van Emmerik, Lee, & Sprague, 1993), even in subjects who did not meet the threshold for dyskinesia as defined by the DISCUS scale (Sprague, Kalachnik et al., 1984; Sprague, White et al., 1984).<br>These findings reveal the strong per-sistence of tardive dyskinesia in a population group with mental retardation to medication withdrawal even over a one-year period of obser-vation. The instrumental measures of postural performance and motor control are more sensi-tive indices of the particular effects of tardive dyskinesia on specific effectors than general rat-ing scales such as the DISCUS (see also Newell, 1996; Newell, van Emmerik, Lee, & Sprague, 1993).

•Physical well-being improved in participants who achieved complete discontinuation. This increase in physical well-being may be related to the attenuation of side-effects, e.g. of parkinsonism.
• Full or attempted discontinuation led to a decline in mental well-being which subsequently recovered. Mental well-being was lower at all time points in the group of participants with incomplete discontinuation, compared to the participants with complete discontinuation. The changes in mental well-being were negatively related to changes in symptoms of irritability and lethargy, indicating an increase in mental well-being when there is a decrease in challenging behaviours.
•The effects of discontinuation on social functioning were similar to those on role limitations caused by physical or mental problems.
•Overall, the results indicate that participants with incomplete discontinuation initially showed worse functioning, at the time point of scheduled discontinuation, which improved again at follow-up, after discontinuation had been suspended.
•The results indicate that an increase in irritability and lethargy during discontinuation will decrease social functioning. Furthermore, the results also indicate that when symptoms of parkinsonism or autonomic symptoms decrease during discontinuation, experienced role limitations will improve.
•The changes in mental well-being were negatively related to changes in symptoms of irritability and lethargy, indicating an increase in mental well-being when there is a decrease in challenging behaviours

Our success of engagement was highlighted by:

•no breakdowns of placement or

•no hospital admissions upon withdrawal

•and successful identification of relevant biochem-istry changes in patients which otherwise would not have been possible without the structured programme of withdrawal.

As a result of the Cornwall experience, the present authors have created a new refined template for clinic letters from second-ary care to the local GPs. This is now used for new referrals where antipsychotics are considered either as a new prescription or for the

renewal by secondary care medical team. This incorporates all alter-natives to prescribing antipsychotics, a diagnosis and any rationale for medication use along with a possible formulation.

exprience of carers:

It is important to understand the reasons for patient/carer's concern regarding with- drawal. In our study almost unanimously, carers and people with in- tellectual disabilities expressed concern regarding the withdrawal of antipsychotics as they did not want to take the risk of upsetting the status quo. The present authors found that a frank discussion with them about the pros and cons of withdrawal was very helpful. The carers will feel reassured when it is highlighted to them that it has been shown from other studies that withdrawal is possible in a high proportion of cases. Nevertheless, it should also be highlighted that the attempt to withdraw will fail in some cases. Under those circum- stances, the patients and carers should be reassured that there is a structured back-up plan available as in Figure 1. They should also be informed that in several cases worsening of behaviour upon with- drawal is not due to the resurgence of previous problem behaviour but due to withdrawal symptoms which are likely to disappear in many patients after a few weeks. As required PRN medication could be used in the meantime to tide them over the difficult period of worsening of behaviour. It is also important to highlight the risk of long-term adverse effects from medication and the findings from previous studies that withdrawal will lead to a better quality of life for people with intellectual disabilities. Involving patients and carers helped us to develop local champions and local patient and carer advisory groups consisting of people who are experts by experience. This proved a valuable part of the pathway for withdrawal.

experience of clinicians:

•The use of certain tools as the present authors have done in our study will smooth the path to withdrawal.

>50% dose reduction or discontinuation: 14 (16%)

≤50% dose reduction: 26 (30%)

No change or increased dose: 46 (53%).

•Only nine people (7.6%) were successfully withdrawn from thioridazine with no alternative antipsychotic drugs required, despite this having been attempted for 36 people (a success rate of only 25.0% of people for whom complete withdrawal from antipsychotic drugs was attempted).
•A total of 45 people (37.8%) were withdrawn from thioridazine and switched to an alternative antipsychotic drug without the emergence of new or deterioration in existing mental ill-health or problem behaviours.
•Fourteen hospital admissions to an intellectual disabilities psychiatric assessment and treatment unit resulted from problems associated with thioridazine withdrawal. This totalled 621 days, with two people still in-patients at the time of this study. At a unit cost per day of £172 (€261) for learn¬ing disabilities hospital, this totals £106,812 (€1 62,130).
•Two people required detention under the Mental Health (Scotland) Act, 1984. One person additionally required admission to a general hospital for a total of 10 days, due to drug side effects (transferred during her psychiatric in-patient stay), at a unit cost of £341(€518) per in-patient day,16 totalling £3,410 (€5,176).
•The exact number of hours contact with other professionals as a result of the onset of problems associated with thioridazine withdrawal is unknown, and assumptions are made. It is assumed each community nurse referral resulted in a fortnightly face-to-face contact of one hour each for a period of six months; whereas for those nurses working in the specialist intensive support teams, double this level of contact is assumed. This equates to costs of £47, 320(E71, 827).
•Psychology and social work contact time is assumed to be similar to that of community nursing, but at different costs, totalling £13,572 (E20, 601) for social work and £3,328 (E5,052) for psychology.
• A number of special investigations were conducted, including blood tests, ECGs, two CT scans and other x-rays.
•Ten people required increased levels of carer support to be provided; seven were excluded from a day centre placement, one person experienced a placement breakdown and moved to a new home, and six experienced considerable family problems because of the thioridazine withdrawal. These are considerable human expenses, in addition to having (uncalculated in this paper) associated financial costs.
•The following total excludes the costs to families, additional social and support costs, primary care costs, and special investigations. It also excludes the costs of routine appointments for those people who were successfully withdrawn from thioridazine with no problems. The resultant financial costs to the intellectual disabilities psychiatric service to attend to the new needs people acquired due to withdrawal of thioridazine

Brief Summary of AUTHOR conclusions

The most striking feature of this study was the pauacity of significant findings considering the huge quantity of data available about a multitude of aspects of the patients' lives, behaviours, abilities and personalities

•In this study, 40% of participants with intellectual disability who had been judged by their responsible clinician to be eligible for a withdrawal trial of their long-term, off -label antipsychotic drug were able to discontinue in a time frame of approximately 4-7 months, with, on average, no behavioral worsening.•
To enhance discontinuation off-label drug use, staff's feelings should be explored, their knowledge of psychotropic drugs improved and reasons for clinicians' judgements of participants' behavioural worsening investigated.

The attempts at self-suffocation, which were not associated with dysphoric mood, intent to die, or thought disorder, were evidently not suicide ges-tures in the classic sense. They may have repre-sented complex tics associated with Tourette's disorder or a phenomenon distinct from Tourette's disorder. The study shows that the pharmacist, through a team approach, can help reduce the overuse of medications in institutions for the mentally retarded.

If those charged with the delivery of services to re-tarded persons are to comply with accreditation and li-censing standards and with court orders, they must work to reduce reliance on psychoactive medication in order to achieve therapeutic goals. The two-phase drug assessment program described in this report permits the routine evaluation of each individual's need for psy-choactive medication in a practical, conclusive man-ner.•

It is significant that 14 physicians were employed during the documented time period.
Two to 4 physicians were employed at any one time, with an average length of stay of 22 months. This rather high turnover within the medical staff means that these results cannot be attributed solely to physician prescribing prac-tices. It can be strongly argued that the Behavior and Medication Committee was the primary influence in the trend to reduce use of psycho-tropic medications. Further, the results indicate that the interdisciplinary approach was indeed valuable because the arrival of new physicians did not reverse the overall trend in prescribing.

The experience of the Wyoming State Train¬ing School indicates that psychotropic drugs do have a place in the care of persons with mental retardation. However, it is important that they be used cautiously and only where they are judged to be effective. We recommend that prescribing of psychotropic drugs be part of a team effort involving other therapeutic mea¬sures. The team should ensure that medication is not prescribed in lieu of behavior program¬ming, environmental changes, or staff training. In addition, it is important that the medical chart contain documentation of the purpose for which the medication is prescribed and any evidence of its effectiveness or lack thereof in the individual patient. The goal should always be to achieve the lowest dose of the most appropriate medication needed to manage symptoms, with elimination of the medication altogether whenever possible.

•STOMP is best delivered as a collaboration between the pharmacist and multiple healthcare professionals to ensure safe and effective deprescribing guided by behavioural data and quality of life improvement measures.
•Secondary care pharmacist experts in learning disability and autism are key to delivering STOMP in community teams and in-patient settings within specialist NHS Trusts and private healthcare providers.
•The clinic has been promoted as a model of care through a range of publications and showcased at several conferences. The clinic was also promoted to two local family forums and a number of self advocates, people with a learning disability, autism or both who also act as a 'confirm and challenge group' for service development. This wider collaborative is enshrined in the STOMP pledge for health and social care providers
•There is a widely held belief that people who are solely under the care of GPs will be less complex and might not need the level of support provided by PBS practitioners. The clinic promotes the concept that the collaboration will be more effective and safer for everyone.
•Advice, guidance and direction from secondary care pharmacists and behavioural experts will be key to successful and safe service development and delivery in line with the principles of the NHS 10-year plan, which supports wider adoption and spread of STOMP.
•STOMP is about inclusion and partnership working with people with a learning disability and autism together with their family and care teams. Pharmacists are best placed to educate people about the STOMP agenda and to reach out to include everyone in STOMP service development.
•Pharmacists with expertise in STOMP within secondary care should develop close working relationships with colleagues in medicine optimisation teams and the newly developing Primary Care Networks. This will ensure the specialist expertise is available to support primary care colleagues to safely deliver STOMP.
•STOMP is about inclusion and partnership working with people with a learning disability and autism together with their family and care teams. Pharmacists are best placed to educate people about the STOMP agenda and to reach out to include everyone in STOMP service development.

The results show the considerable impact of a continuous drug utilization process upon psy-chotropic medication use for a population of mentally retarded persons

Our data indicate that federal and state guidelines should be reconsidered. There should be inclusion of the observation that there is only a very small chance that antipsychotic drug-free status can be maintained in individuals who have previously relapsed during a withdrawal attempt. Adherence to guidelines should be consistent with what is good clinical practice. Careful scrutiny of previous records for medication withdrawal attempts is indicated to weigh the likelihood of further relapse and aggression after an antipsychotic drug taper.<br>Guidelines should reflect the observation that for individuals with multiple previous relapses, caution is advised when considering future antipsychotic drug withdrawal attempts. Finally, it should be noted that, although successful substitution of a second-generation antipsychotic medication for a first-generation one usually was successful, it was unsuc¬cessful in at least 4 of our cases.
These observations support policiesand guidelines indicating that attempts to stop treatment withantipsychotic medications in mentally retarded individuals areworthwhile. However, the results also indicate that eventualdiscontinuation of antipsychotic medications in institutionalizedmentally retarded adults who have previously relapsed upon suchwithdrawal is unlikely to be successful. Rigid adherence to drugwithdrawal policies and guidelines in such individuals should bereconsidered.

The data collected in the first two years of operation of an Interdisciplinary Review Committee suggest that the use an advisory committee was effective in reducing the use of psychotropic medication with people with intellectual disabilities in a Victorian residential institutional setting. Of 25 people involved in the review process over a two-year period, 19 had their medication reduced, and a further three were totally off medication. Additionally, for the people reviewed, polypharmacy had been substantially reduced.

•We would like to make some recommendations in regards to clinical and research practice.
•The first is that clinicians should strongly consider giving anticipa¬tory guidance re diet and exercise to children and their parents before initiating tri¬als of antipsychotics. Appreciable weight gain has significant impli¬cations for physical and mental health. Also an explicit state¬ment that the drug will probably be discontinued if weight gain be¬comes a problem that supersedes the benefits of medication is a motivation for parents who see the benefits of risperidone therapy.
•Our second recommendation concerns the desirability of monitoring height and weight in children who are medicated with psychotropic medicines in general. The reality is that many psychotropic drugs do affect appetite and/or weight, and furthermore, that the devel¬opmental period may be impor¬tant in determining adult growth outcomes
•Third, we were struck by the logic and utility of BMI-Z scores and WE-Z scores. Such scores automatically correct for the child's age and gender and, unlike both absolute BMI and weight, no correction is needed to take account of the child's devel¬opmental stage. Hence, the 0.60 SD increase seen from treatment before risperidone to termination was readily understood in its own right, without reference to con¬version tables. The CDC norms are based on tens of thousands of young people. They are available free, they are easy to use, and they are easily interpreted without hav¬ing to resort to charts
•The prospect of reversibility may provide some comfort for clinicians and parents alike, but we need far more data before we can assume that this is the case for all children.

•First, over the course of the study, an overall reduction in antipsychotic dos-age was associated with an improvement rather than a deterioration in behavior. This finding is consistent with the results of controlled studies (Singh & Amen, 1981). Because the study was naturalistic without random assignment to various treatment groups, it is impossible to kriow whether medication reduction led to be-havioral improvement or vice versa. As noted earlier, one of.the three reasons for medication reduction was improved or stable behavior; therefore, it is our impres-sion that the targeted behaviors/symptoms improved first, resulting in medication reduction.
•Second, the presence of psychosis was associated with an increase in anti- psychotics, whereas its absence was associated
with a large decrease in dosage. .Because the majoritjtif the psychotie indi-vidurils in this study were diagnosed es having schizophrenia .(45 of 48), this may simply reflect the fact that individuals . with schizophrenia, retardleris of level of intellectual functioning, show a similar therapeutic benefit from antipsychotica • (Menolascino, Wilson, Golden, & Rued-rich, 19F6) and generally require extended . treatment with- these agents.
•Third, the use of alternative medics-
. tions was associated with a reduction in • antipsychotic usage. This is consistent with a growinvliterature on the ability of nomuiticrsychotic agents to reduce aggres-sion an other behavioral disorders in individuals with Mentalreta'rdation. Spe-cifically, lithium has been found effective in at least three controlled studies
To the extent that individuals with mental retardation appear to have an in-creased risk for tardive dyskinesia (Gual tieri, Breuning, Quade, & Schroeder, 1982), the reduction of antipsychotic inedicationsin this population must re¬main a clinical and policy priority. These results identify some variables that may predict which individuals will probably have successful antipsychotic medication reductions. These findings suggest that . individualswithout psychoses are a par¬ticularly suitable group for such an ap¬proach whereas those s,,ith psychoses msy not be. In addition, these findings suggest that the reduction of antipsychotic medi¬ation for some individuals may be facili¬tated by the use of alternative medication, so tha: &general policy of discouraging psychotropic usage may impede, not enhance, the reduction of antipsychotic medications.

Changes in the behavior of these severely retarded adults which we attributed to chlorpromazine were diverse and generally of no clear relevance to the patients' well being, access to the environment, or physical or psychological comfort. In the workshop environment where reinforcement was available for persistence at simple repetitive tasks, one subject behaved more appropriately when he was drug free; none improved when they were given CPZ. The generality of these findings is by no means established. This investigation does suggest, however, that short-term reversal designs are appropriate in the study of phenothiazinebehavior interactions. It may in fact be possible to evaluate the effects of drug discontinuation in 3 to 5 days rather than the currently accepted 2 to 6 weeks. This methodology is minimally intrusive and produced no observable patient management problems or behavioral or physiological distress.

•Those individuals who demonstrate persistent severe behavior problems (Group 3) require careful neuropsychiatric evaluation and most likely will re-quire a specific psychotherapeutic agent. However, many who are tapered will improve without the use of psychoactive medication (14/23, or 61%, in our study) (Groups 1 and 2), and even those with per-sistent behavior problems can, infrequently, be man-aged nonpharmacologically with behavioral and environmental techniques (2 patients in Group 3).<br>•The explanation for the syndromes of "progres-sive improvement" (Group 2) and "transient worsen-ing" (Group 1) is unclear. The patients in Group 2 might have been experiencing neuroleptic induced akathisia while those in Group 1 may have been bet-ter able to respond to behavioral programs once the neuroleptic was removed. Alternatively those in Group 1 may have been demonstrating a prolonged but transient "cholinergic supersensitivity" (9), ad-renergic rebound (10), neuroleptic-induced supersen-sitivity psychosis (11), or tardive akathisia (5) as has been suggested previously.<br>•The emergent behavioral symptoms varied from patient to patient and included slapping, hair pulling, rectal picking, head banging, rumination, tantrums, and assaults. •Therefore, in patients with profound mental retardation, the symptoms that ap¬pear during withdrawal of chronic neuroleptic use are nonspecific and may reflect a general state of agi¬tation secondary to decreased sedation, or as was found in the "persistent" group, symptoms of an un¬derlying neuropsychiatric disorder.<br>The clinical challenge encountered with the profoundly re¬tarded is the achievement of an accurate psychiatric diagnosis. Perhaps neuroradiological (12-14) and psychoneuroendocrinological (15-17) biological markers may help provide the necessary diagnostic precision.
These findings have a number of implications for the consideration of tar-dive dyskinesia in clinical settings. First, they indicate the value of repeated obser-vation or testing of an individual over long time periods. Second, they re-em-phasize that the rate and level of behav-ioral change following medication withdrawal is individual-specific and that in some cases tardive dyskinesia is still prevalent even after one year post-withdrawal of medication. The third im-plication is that changes in the amount (i.e., amplitude) of movement are usually related to changes in the organization (structure) of movement output and that often the latter measures are more sensi-tive to behavioral change than the former

In summary, the data from this prospective study of adults with MR clearly revealed that there is a changing pattern to the effector profile of TD over the course of neuroleptic medication reduction and withdrawal. This trend was apparent on both a group and an individual basis. These findings suggest that dyskinesia may be best viewed as a dynamic disorder whose manifestations change in im-portant ways over time. Furthermore, the data from this study support a general model of movement disorders in TD (Newell, 1996a), which holds that increasing clinical man-ifestations of the disorder are directly related to a progres-sive loss of the degrees of freedom available to control motor actions.

•Physical well-being showed an increase in the group that had achieved complete discontinuation.
• Social functioning showed a decrease in the group that incompletely discontinued, which recovered at follow-up.
•Mental well-being decreased at 16 weeks, but recovered at follow-up, regardless of complete or incomplete discontinuation.
•To conclude, discontinuation of antipsychotics had a positive effect on physical well-being when complete discontinuation was possible. When complete discontinuation was not possible, there was a negative effect on health-related quality of life domains.
•However, none of the unfavourable effects were irreversible. Clinical implications
The results of this study have implications for clinical practice. There is evidence that successful complete discontinuation of antipsychotic drugs has beneficiary effects on physical well-being. Even if unsuccessful discontinuation may negatively affect the mental functioning of the client, this will only be temporary.
Conclusion
To conclude, health-related quality of life is both positively and negatively influenced by the discontinuation of antipsychotic drugs. The changes in domains of health-related quality of life are negatively associated with changes in challenging behaviours and symptoms of parkinsonism.
Physical well-being improves when complete discontinuation is possible. Mental well-being can be expected to show a temporary decrease, independent of the achievement of complete discontinuation. Furthermore, in those with incomplete discontinuation social functioning is clearly affected however this is also temporarily. These results suggest that attempting discontinuation will not permanently affect the domains of health-related quality of life. Moreover, when complete discontinuation of antipsychotic drugs is possible, an increase in physical well-being may be expected.

•The present authors managed to withdraw antipsychotics totally among 46.5% (33/71) and reduced over 50% of dosage in another 11.3% (8/71) of adults with intellectual disabilities.

•At three months follow-up no one required hospital admission or change in placement.

•Only nine out of 119 people in our study were successfully
withdrawn from antipsychotic drugs with no alternative
a n t i p s y chotic drug being required (7.6%).
•Whilst 61 people were withdrawn from thioridazine without experiencing new onset or deterioration in problem behaviour or mental illhealth, their mean chlorpromazine equivalent dose of antipsychotics was higher following, compared with prior to, withdrawal.
•The programme of withdrawal precipitated problem behaviours or mental ill-health in half of the people, and
drug side effects in a quarter. This is a significant morbidity, with associated social and family consequences, as well as cost. We consider this to be a poor clinical outcome

Funding Bias

Funded by Stichting Zorgondwesteuning,
Soesterberg, The Netherlands

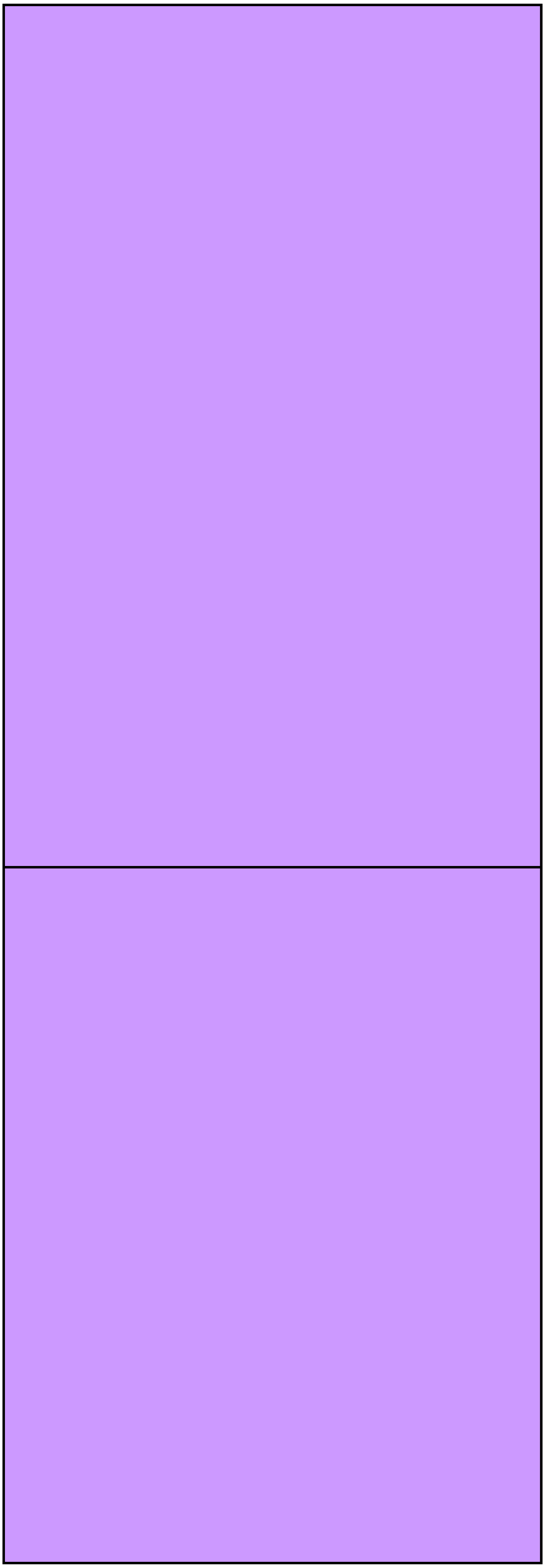

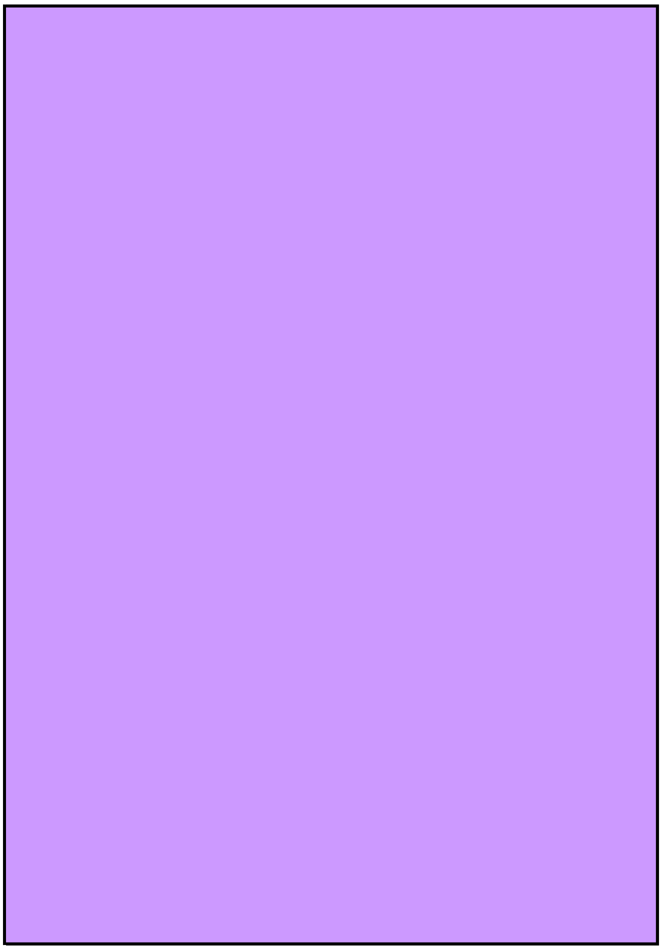

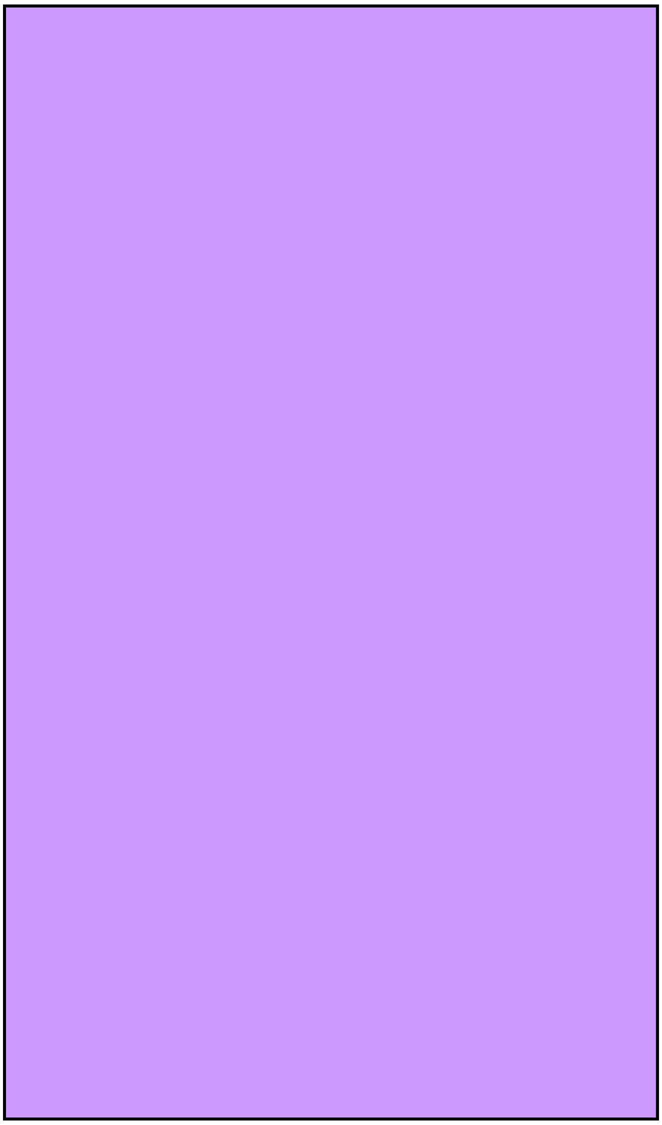

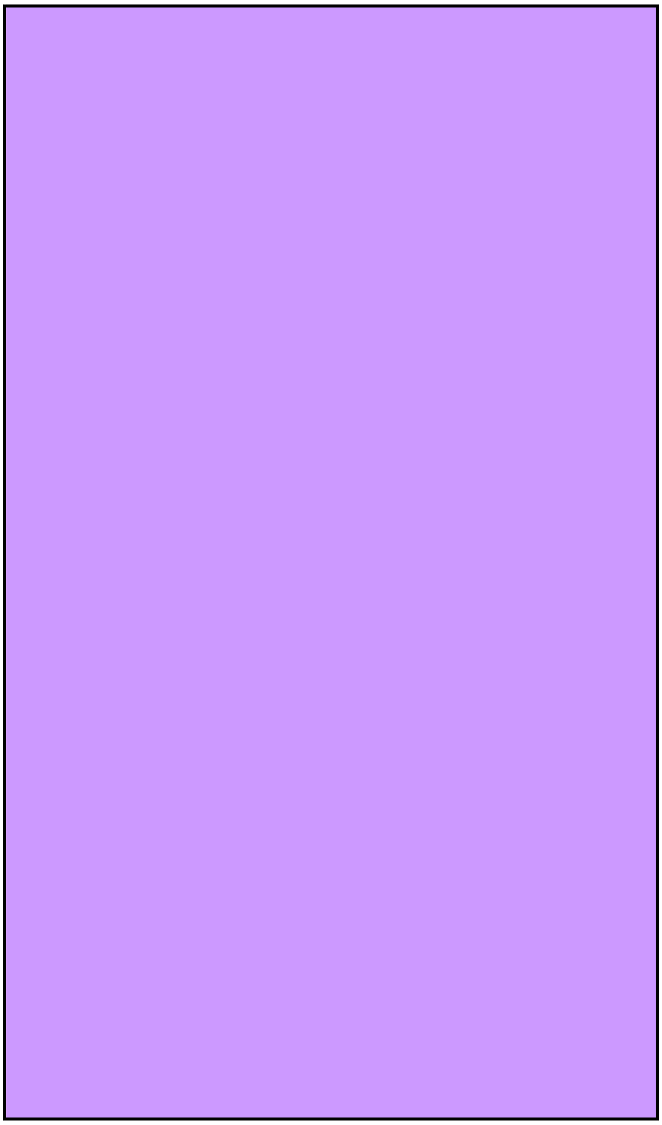

In this report, we describe an IDT evaluation and treatment program for polypharmacy in a community based cohort of individuals with MR/DD. As the result of this intervention, there has been a decrease in polypharmacy, class changes in psychoactive medication, discontinuation of unnecessary anticonvulsants. In addition to the indication for treating seizures, anticonvulsants were recommended for mood disorders but not for aggressive behaviors or impulsivity. In the latter case, atypical antipsychotics proved more useful.

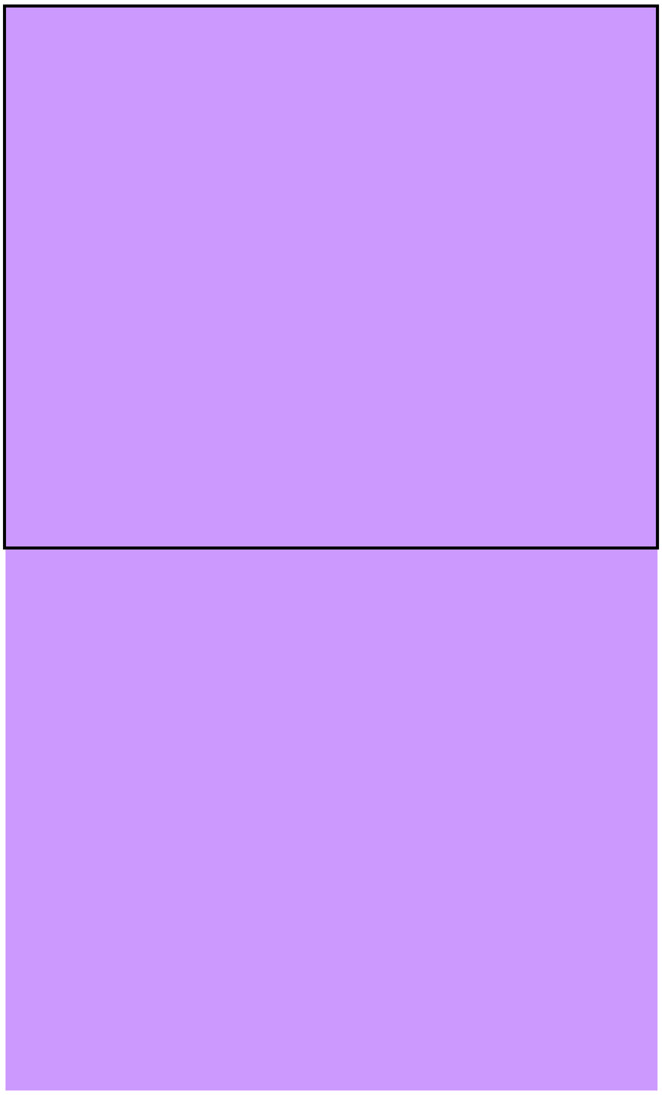

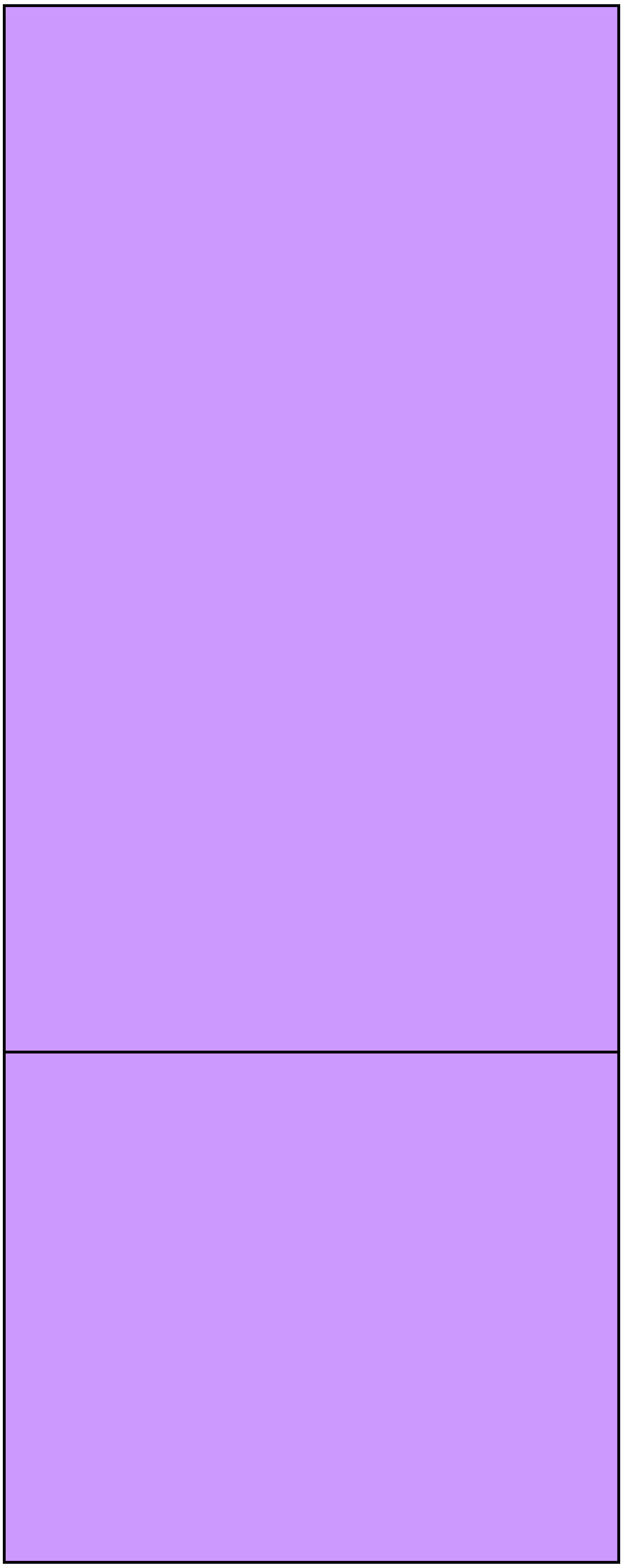

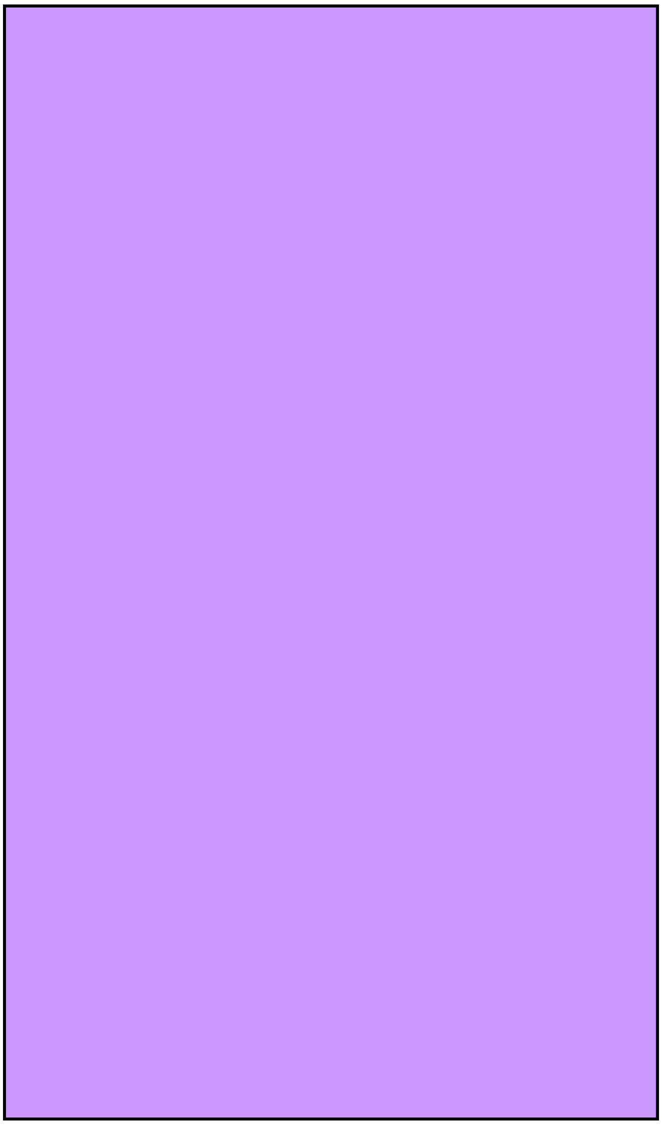

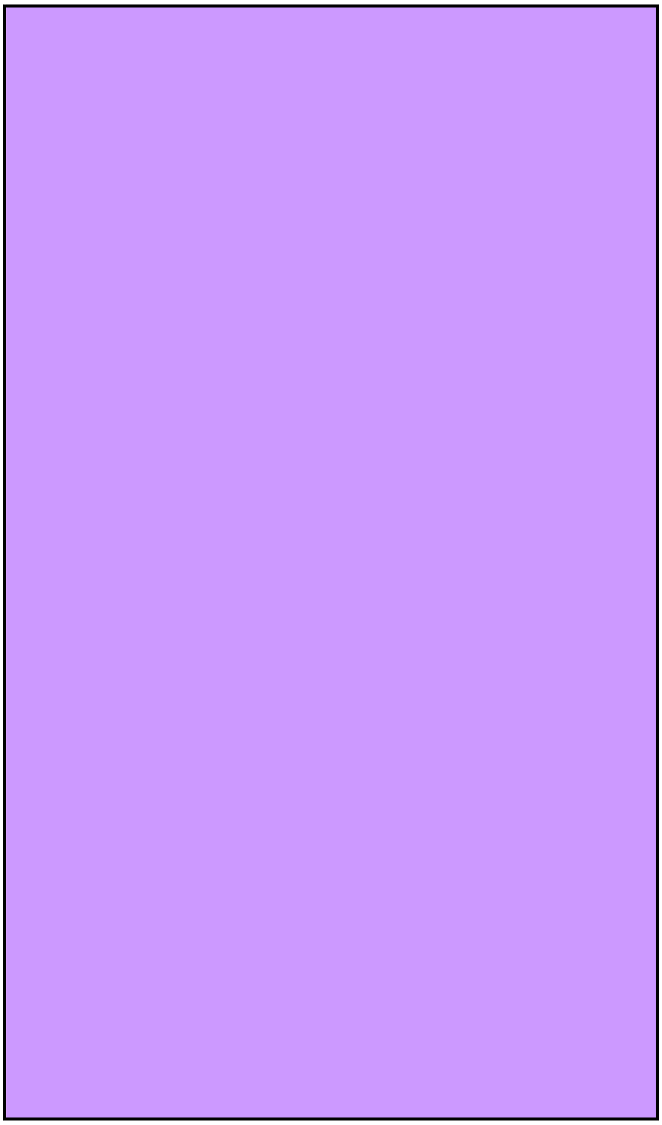

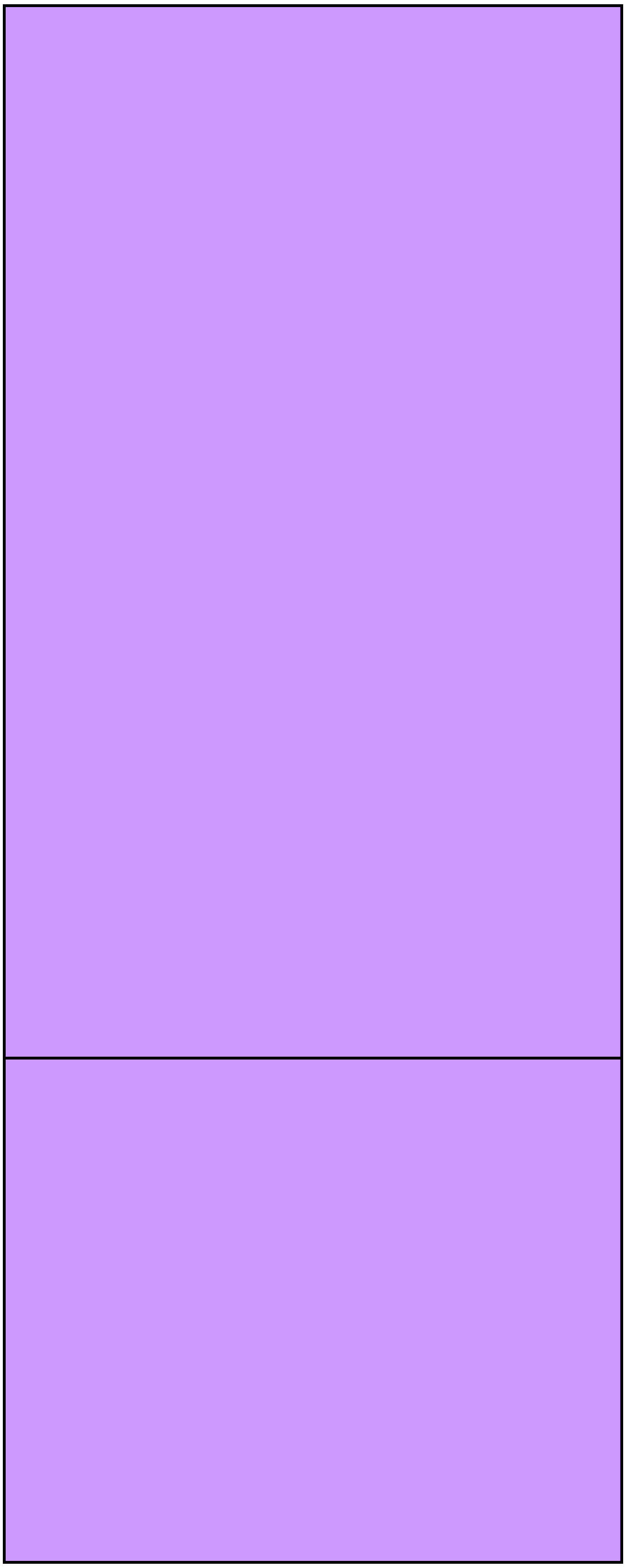

SKF supplied the placebo medication

This research was supported by Grants No. NTH 5 RO1 HD34027-03 and 5 RO1 NS35694-11. We thank Dawn Parker, Caroline Freeze, Kia Handley, Regina Freer, and Courtney Davis for help with data collection and analysis. Requests for reprints may be sent to Karl M. Newell, Department of Kinesiology, The Pennsylvania State University, 146 REC Building, University Park, PA 16802.

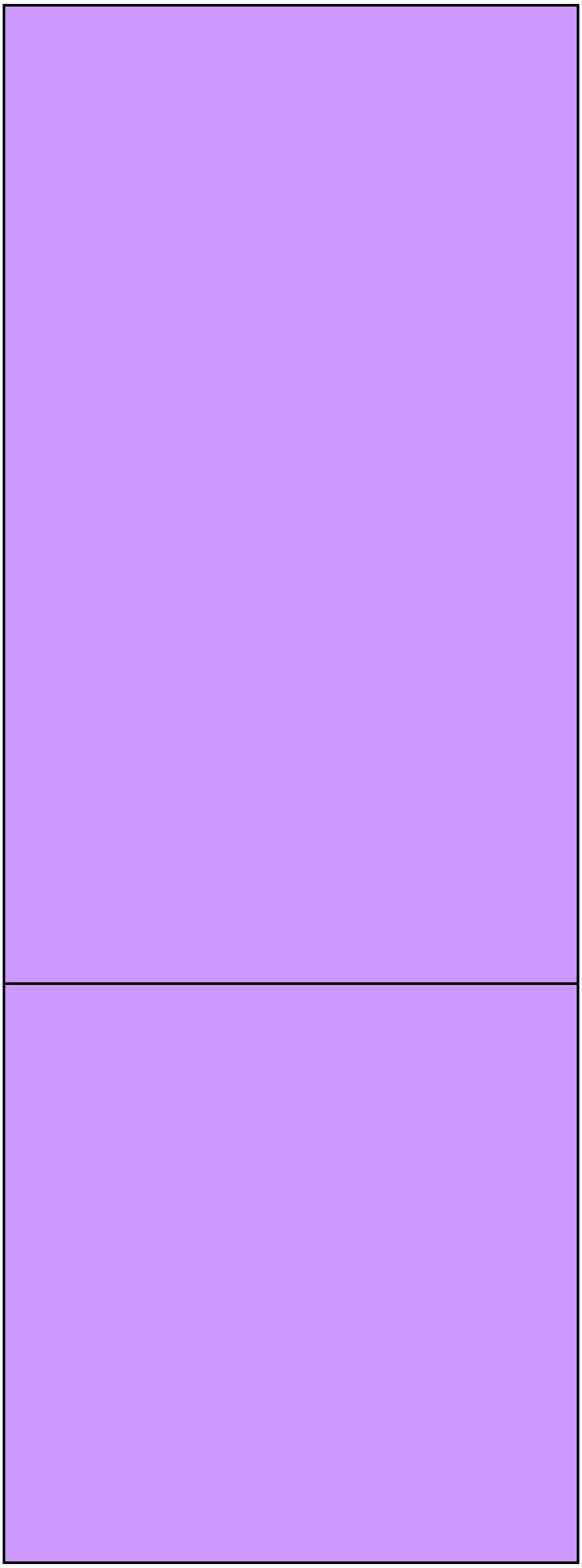

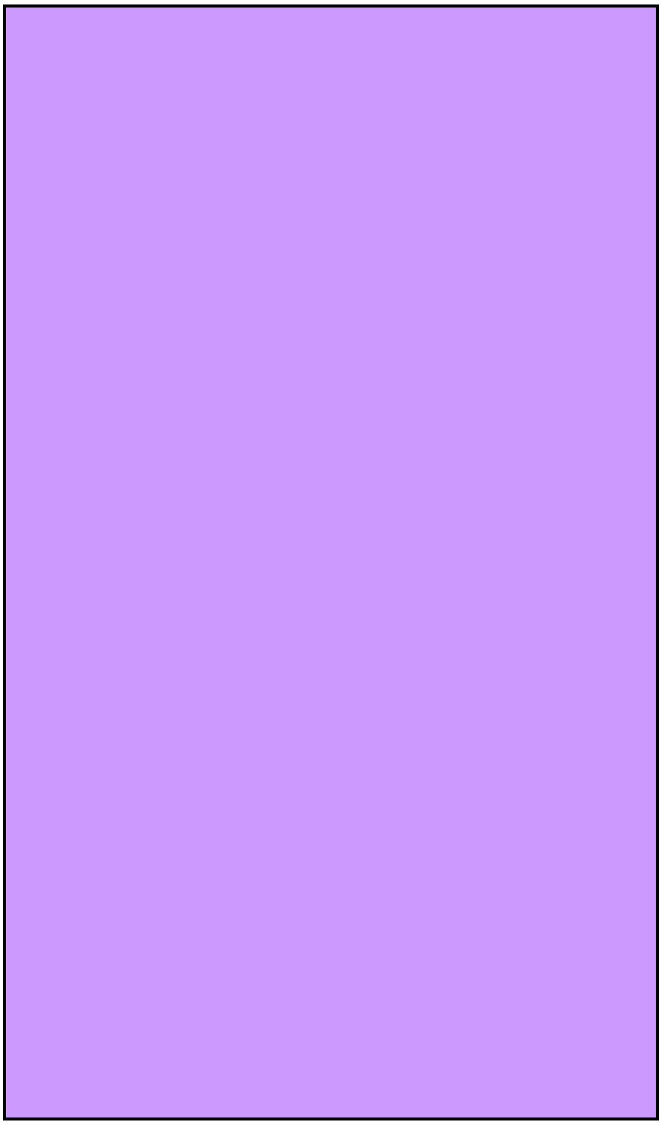

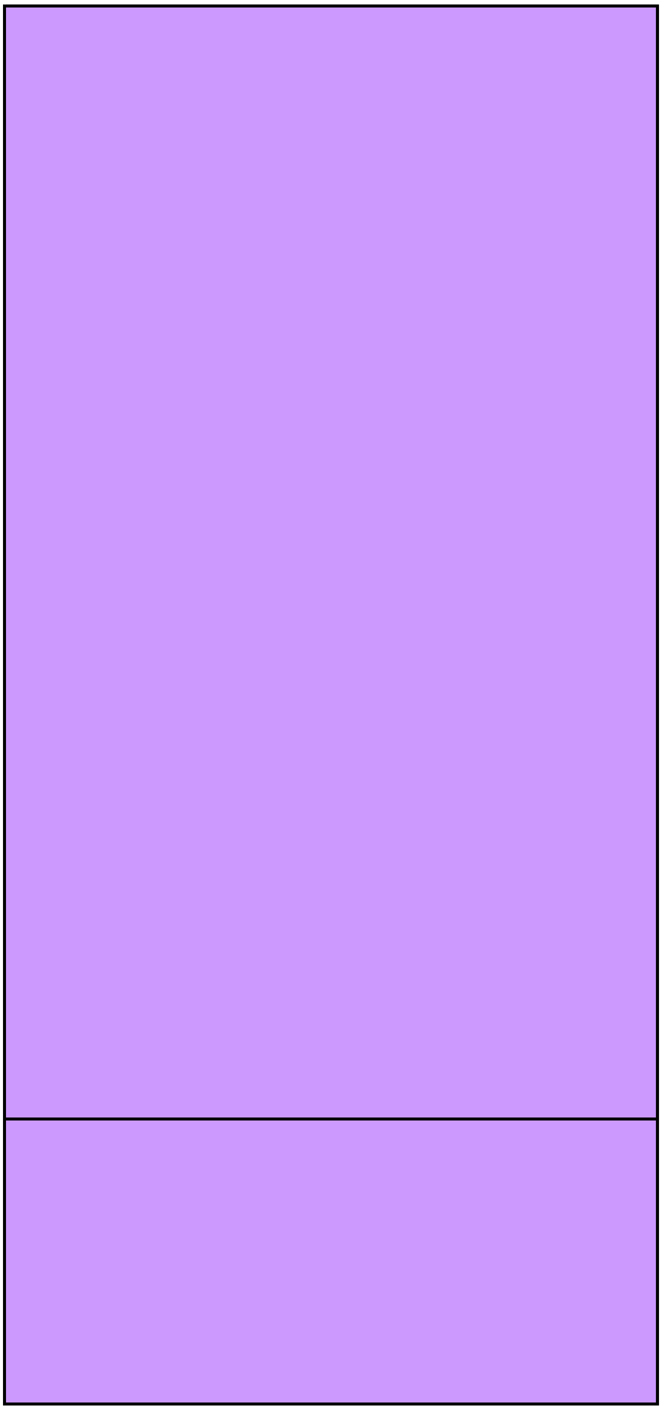

•Good practice requires the avoidance of unnecessary risk, and it could be argued that the financial cost per person (excluding costs of family and social supports and primary care) is not large if there is health gain, or any deaths prevented.
•Current knowledge of the extent to which risk of death is associated with antipsychotic drugs, as opposed to underlying disease or its associations (eg. smoking) is limited. Conversely, the human cost of this thioridazine withdrawal programme has been considerable, and introduced other potentially life threatening risks such as precipitation of neuroleptic malignant syndrome and consequences of serious mental ill-health.
• A potential positive aspect of drug withdrawal however is that it presents the opportunity to fully evaluate whether or not the person has underlying mental ill-health previously categorised as problem behaviour.
•The only factors we identified that were associated with poor outcome were increased severity of intellectual disabilities and female gender.
Our results suggest that antipsychotic drugs do help in the management of problem behaviours for some people. This may interplay with severity of intellectual disabilities, in that possible explanatory mechanisms may be:
•Problem behaviours may be a marker of psychotic disorders in some people with no verbal

Study limitations identified by authors
See study below
ECG changes may be related to individual cardiac function variation or other medication related effects

•A strength of the present study is that it reflects discontinuation in clinical practice with regard to the course and results of discontinuation trajectories. We took the influence of negative changes in health conditions and environmental circumstances into consideration.
•However, recruitment of participants was difficult. In only half of cases eligible for enrolment did physicians judge their clients eligible to discontinue, and of those, just 26% consented to participate in the study.
• Fear by clinicians, caregivers, and legal representatives of clients' behavioral worsening were main reasons not to participate. McNamara et al14 also encountered major recruitment problems in their double-blind, randomized, controlled discontinuation trial of risperidone used for challenging behaviors. They suggested that lack of alternative behavioral interventions to manage potential re-emergence of clients' challenging behaviors may have been the reason for poor recruitment.
•Another limitation of this study was missing data, which reduced the available cases for multivariate analysis. Therefore, the results of this [illegible]

This study had some limitations which should be acknowledged. A major limitation was the missing data of support professionals, which we could not overcome, because we had to rely on
their willingness to complete the questionnaires. Approximately just over half of professionals completed all questionnaires. This lack of data limited the possibilities to identify determinants of successful discontinuation.
Another limitation was that we did not investigate clinicians' characteristics.
A strength of the study is the prospective design in a clinical setting, the naturalistic nature and the relatively long follow-up period
of six months. We were thus able to identify staff-related determinants for successful discontinuation at the short-term and at a longer
term, while accounting for participants' characteristics.

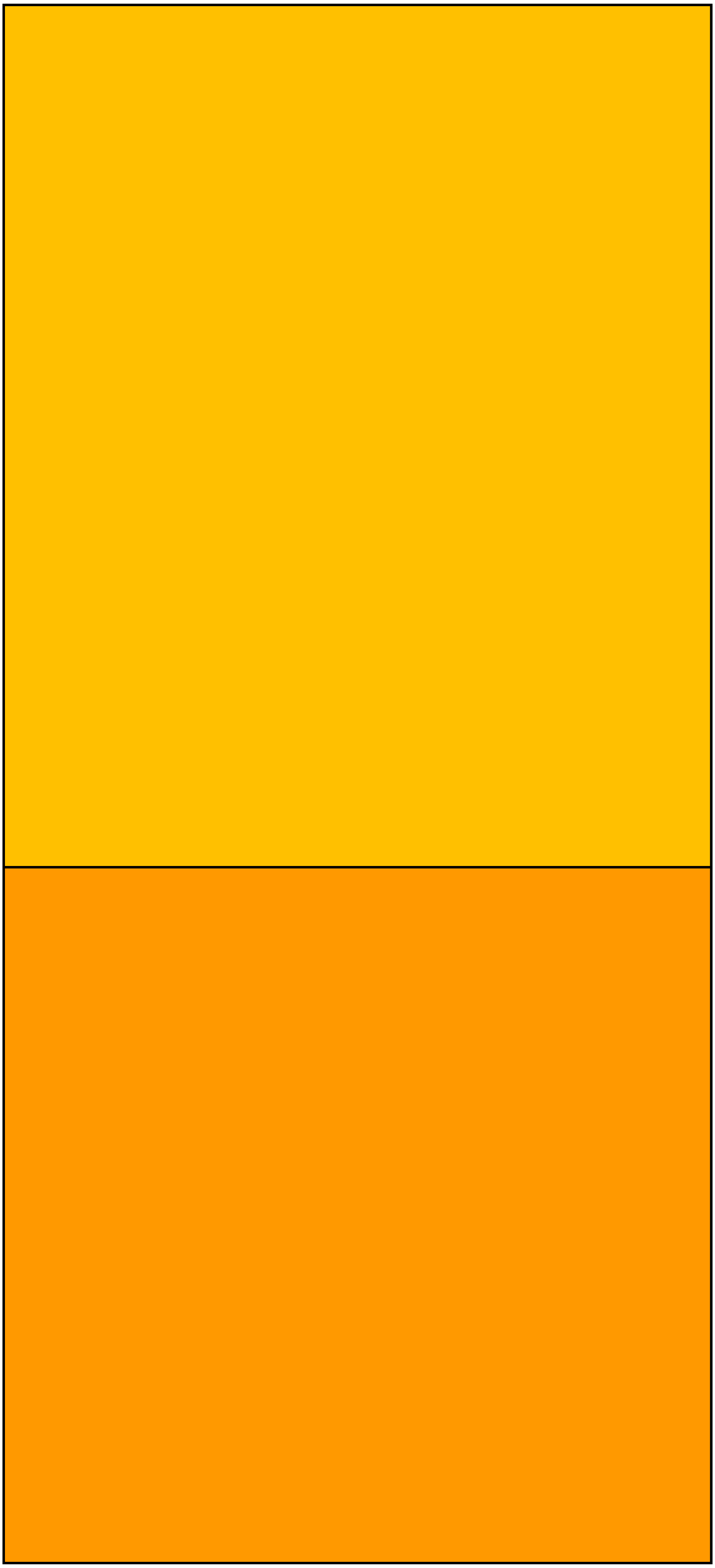

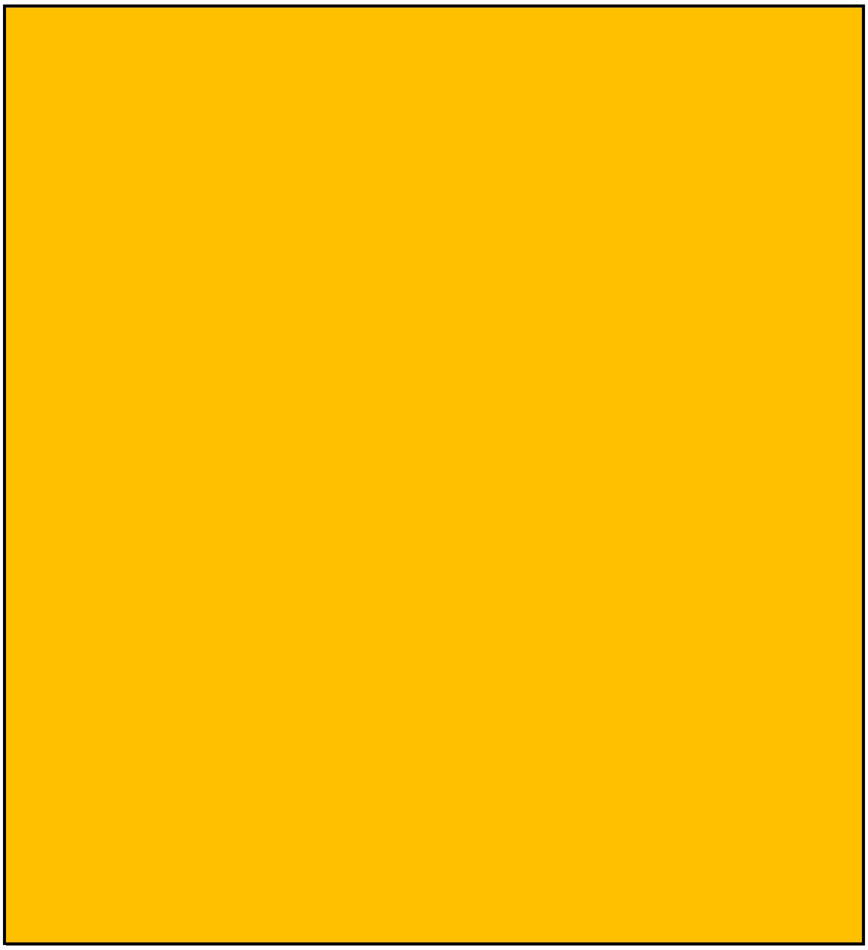

It is significant that 14 physicians were employed during the documented time period. Two to 4 physicians were employed at any one time, with an average length of stay of 22 months. This rather high turnover within the medical staff means that these results cannot be attributed solely to physician prescribing prac-tices. It can be strongly argued that the Behavior and Medication Committee was the primary influence in the trend to reduce use of psycho-tropic medications. Further, the results indicate that the interdisciplinary approach was indeed valuable because the arrival of new physicians did not reverse the overall trend in prescribing.

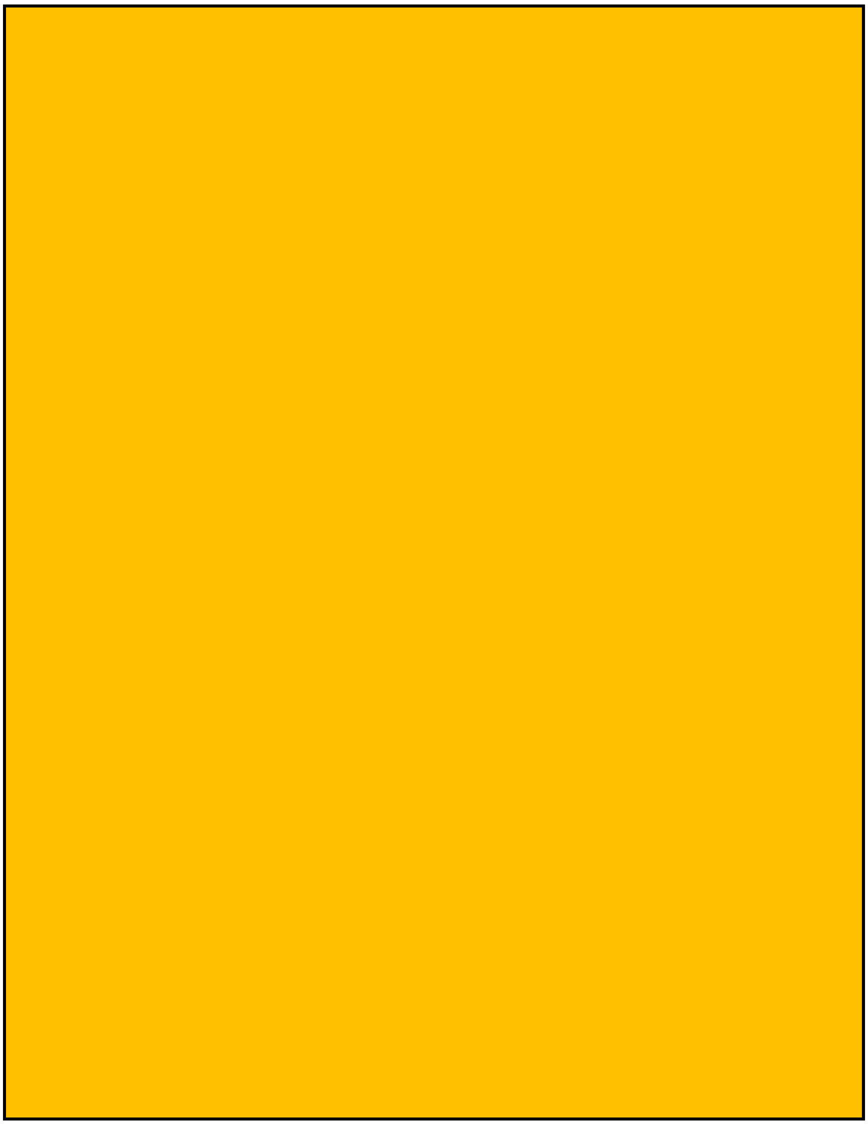

However, because of limited avail-ability of clinical notes on a patient's behavior problems, and little follow-up observation with regard to the drug effect prior to the introduc-tion of the dinical pharmacy services, objective comparison of pre- and post-study impact is not possible.

There are some limitations as to how much one can generalize from our results. Our subject group consisted of a subgroup of institutionalized adults who had severe or profound intellectual disability. In our population, assigning a psychiatric diagnosis is problematic at best. Also, sophisti-cated behavioral controls and high staff-patient ratios were available in our setting. Furthermore, we did not explore the outcomes of those individuals who were discharged from the Murdoch Center during the study period. Also, the doses and presence of concomitant medications were not necessarily held constant.

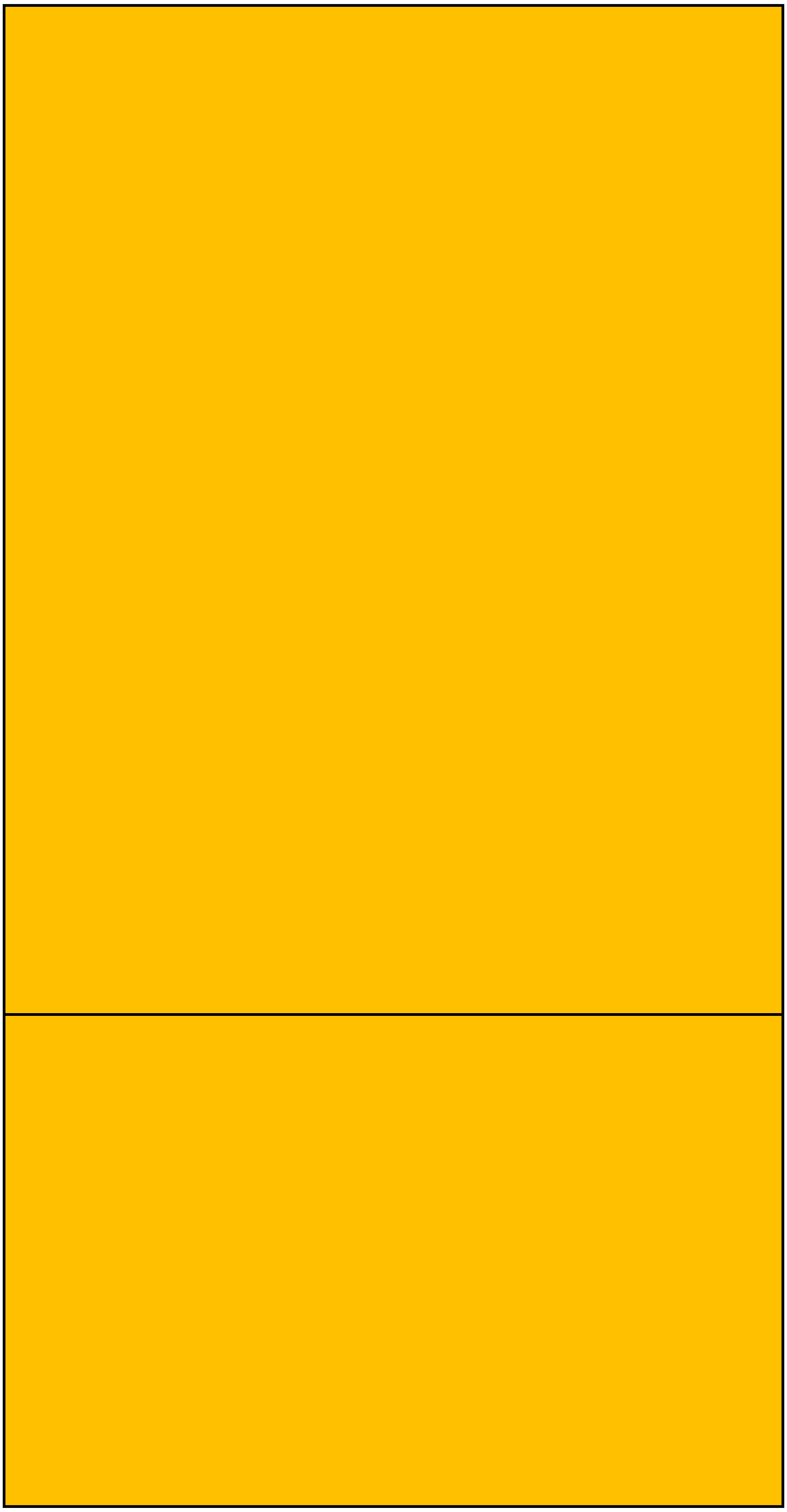

There are several limitations to this study, so readers should in-terpret our findings with due cau-tion.
First, the sample size (n = 14) was small, and the fact that we lost data on some participants re-sulted in even smaller follow-up cohorts.
Second, we had to use weight as our primary outcome measure, although BMI would have been better. However, sub-group comparisons of WE-Z and BMI- Z suggested considerable consistency between the two.
Third, not all participants were as-sessed on the same scale, which introduces a degree of variability. However, all participants were measured on reliable balance scales, either in physicians' or school offices.
Fourth, we did not have complete data on subse-quent medication trials for these children. What data we do have (14 subjects) indicated that the children continued with the med-icines (psychostimulants for at-tention deficit hyperactivity disor-der [ADHD] and antihistamines and chloral hydrate for sleep) that were permitted in the study of disruptive behavior disorders.
We are not aware of subjects' use of any medicines, other than stim-ulants, that might cause reduc-tions in weight
Such outcome data in chil¬dren are understandably sparse, and this type of research is com¬plicated by the fact that most chil¬dren gain weight over time

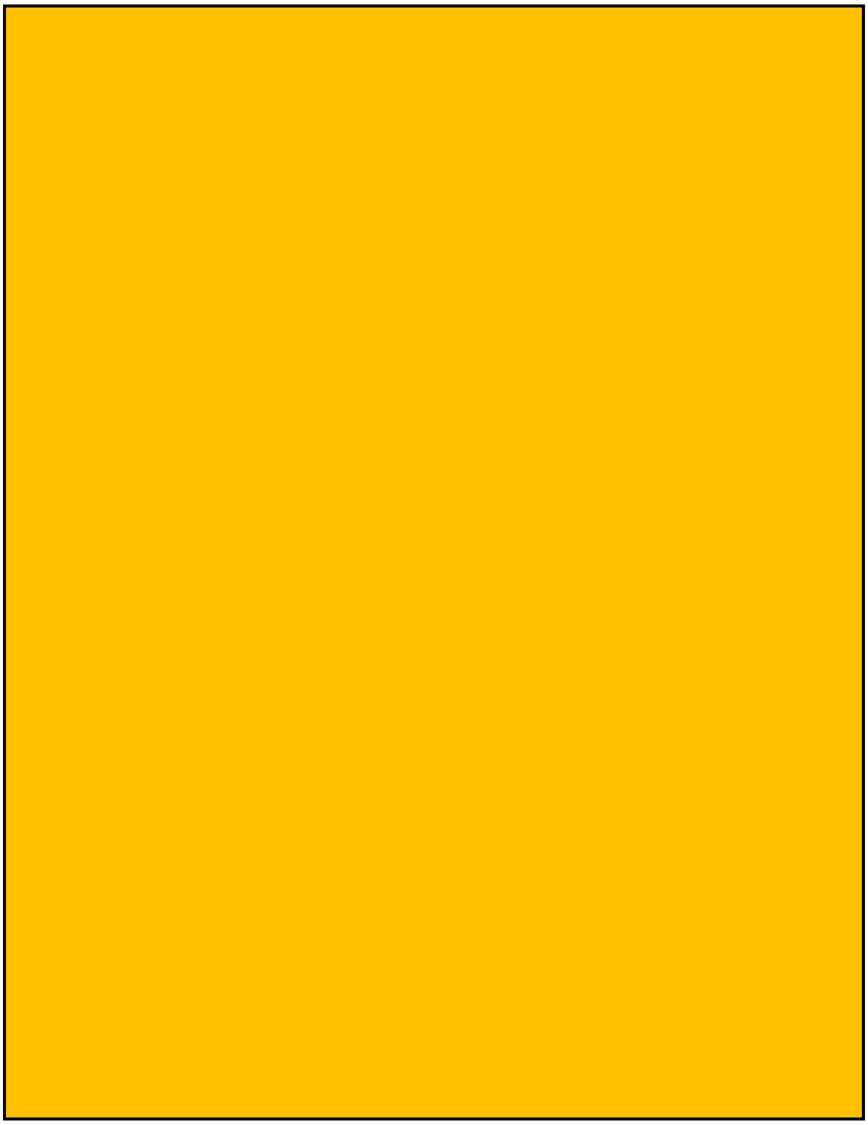

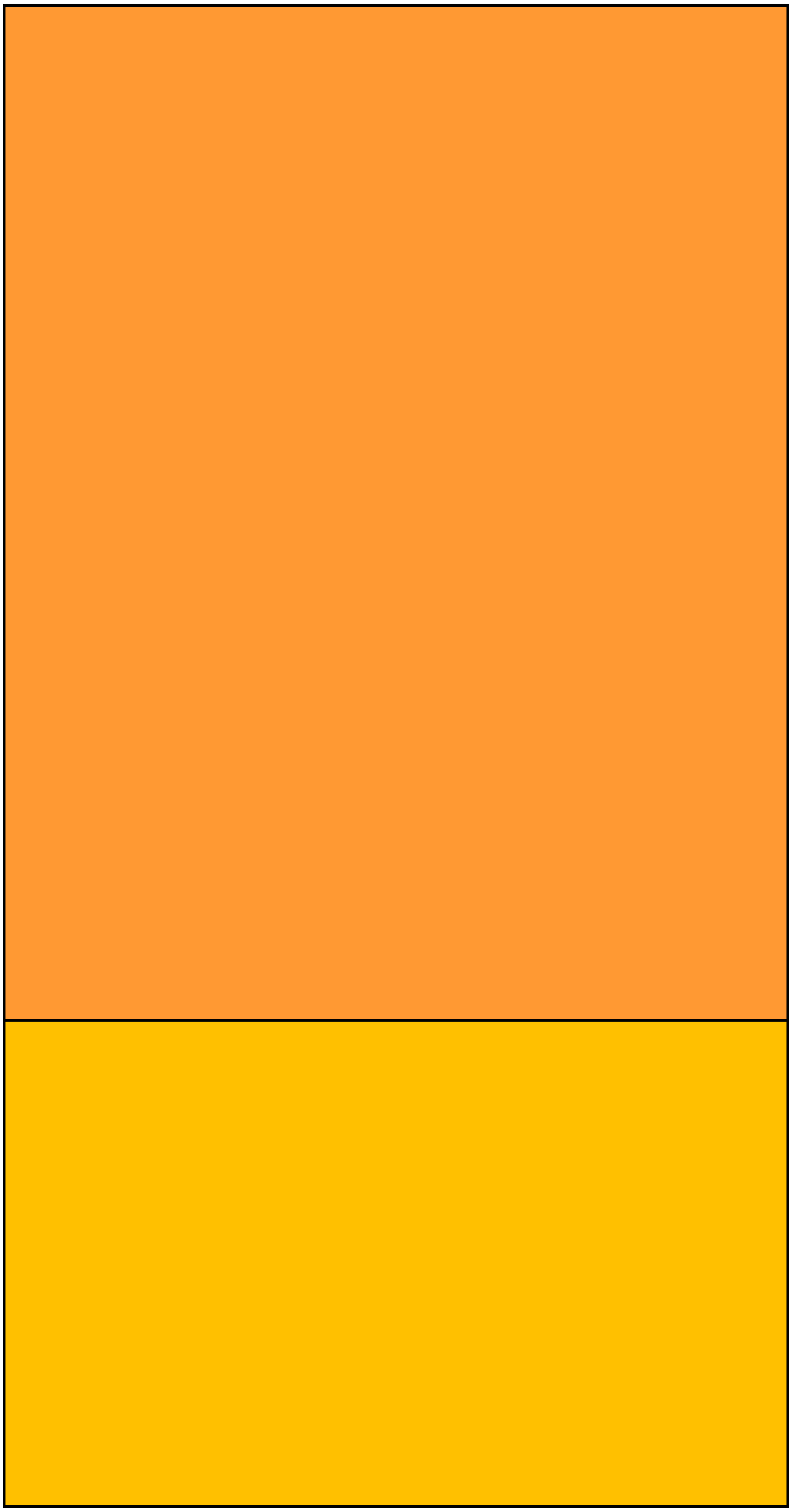

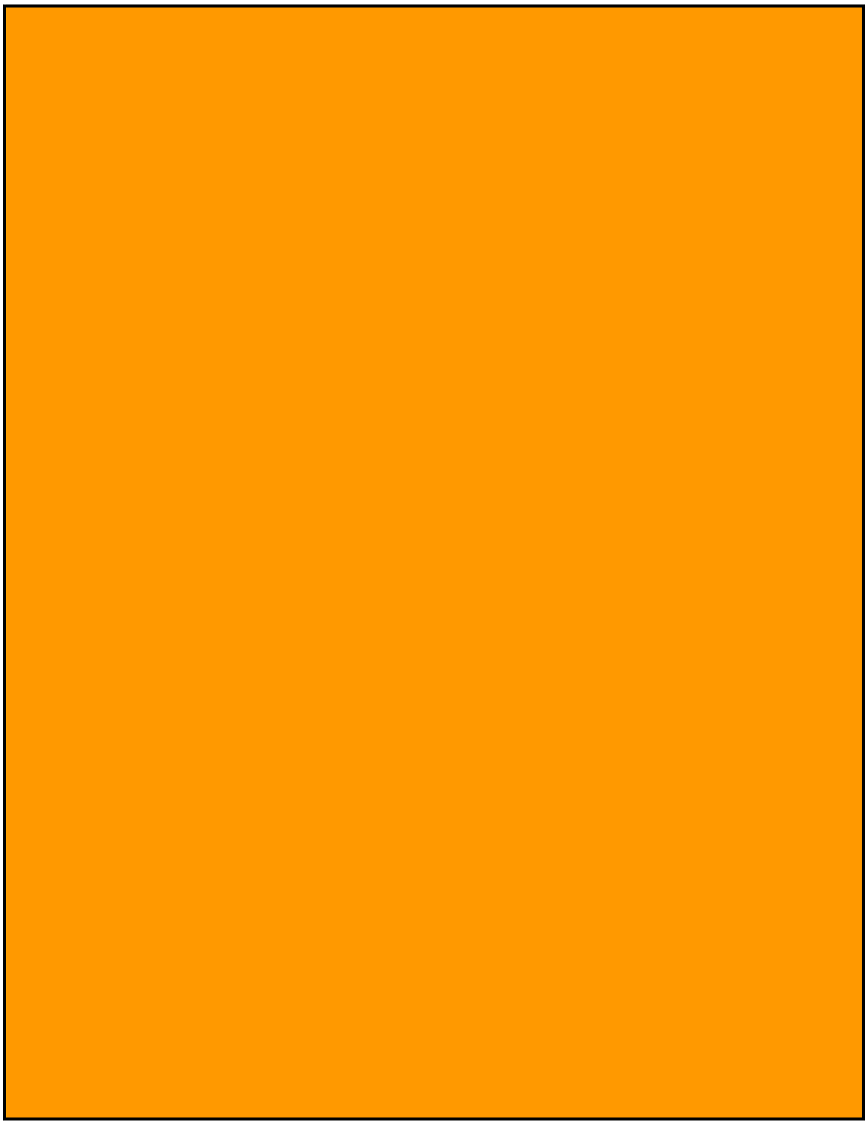

There were insufficient numbers of patients in our study to be conclusive about which patients can or cannot be readily tapered from neuroleptics; how-ever, the data suggest that adult male profoundly re-tarded individuals who are older, have a seizure disorder, or have a low frequency of aberrant (espe-cially assaultive) behavior while receiving a relatively large dose of neuroleptic may be appropriately treated with neuroleptics.

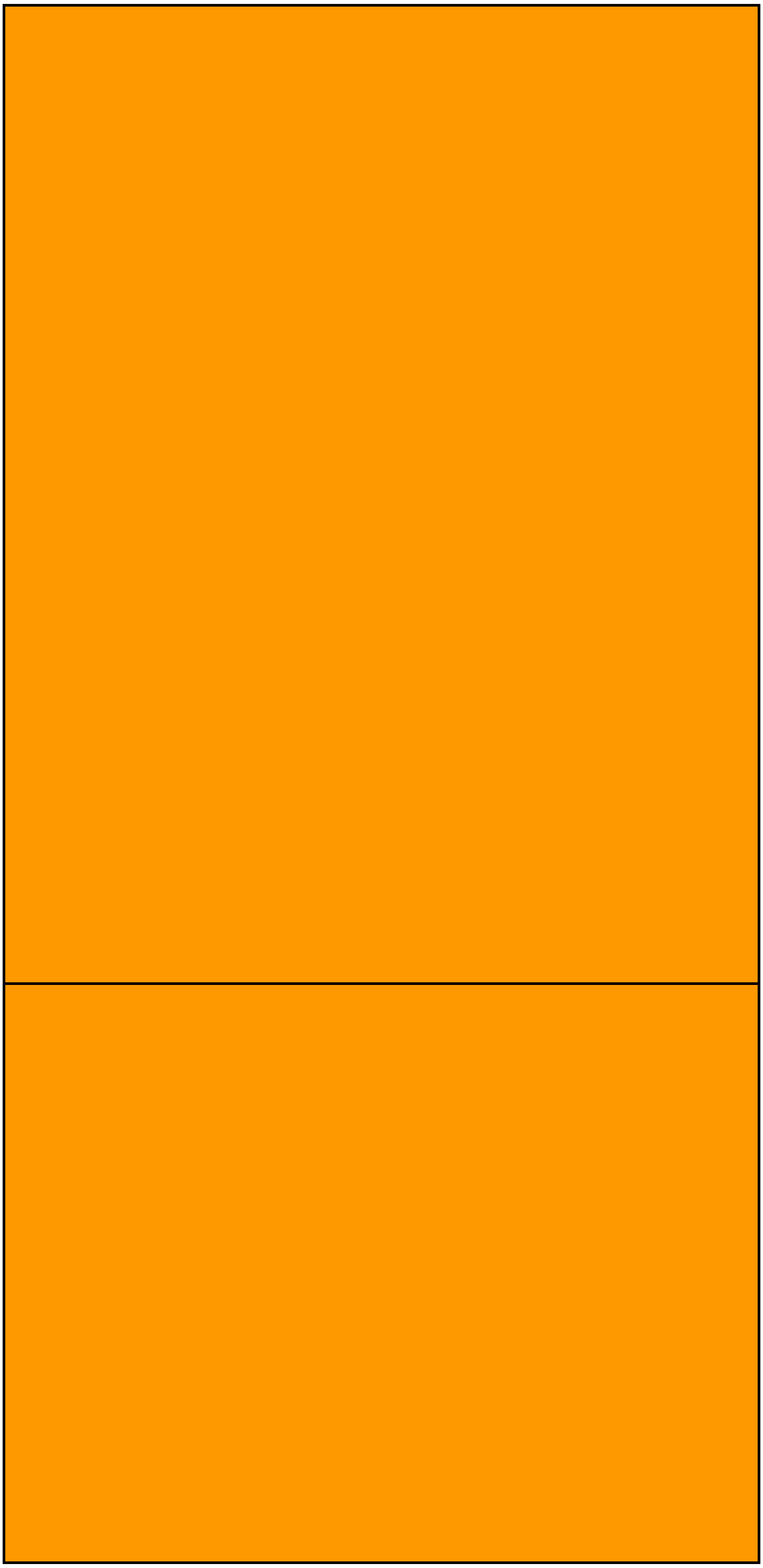

The partiicpants had to be able to follow the instructions for the task ar

•Although the pathway and the tools the present authors have used within our concerted programme of withdrawal seem useful, none of them have been properly field tested. Therefore, the pres- ent authors do not know about their psychometric properties, which the present authors hope to address in a future project.
•The pres- ent authors also did not have a control group to assess whether our pathway is any better than the treatment as usual (TAU) group.
•Our study is also restricted to one geographic area, so the present au- thors would not know whether these findings could be generalized throughout the country or not.
•Although our study has shown that with a concerted effort and involving all stakeholders particularly including people with intellectual disabilities and their carers from the outset could achieve antipsychotic discontinuation and dose reduction in a reasonable number of participants, the programme was time-consuming which was associated with an additional cost to the NHS. However, the present authors feel that by putting more time and resource in the immediate future a programme like ours would have potential to save more time and resources, and human sufferings in the long run. This could be tested in a future study by carrying out an appropriate economic evaluation.
•Therefore, there is an urgent need to put the Cornwall experience in test by running an open-label multi-centre cluster randomized controlled trial (RCT) where participants will be allocated randomly to either an inter- vention group which will implement a structured discontinuation programme and compare the outcome with a control group of TAU for whom no structured discontinuation programme will be imple- mented using standardized outcome measures including

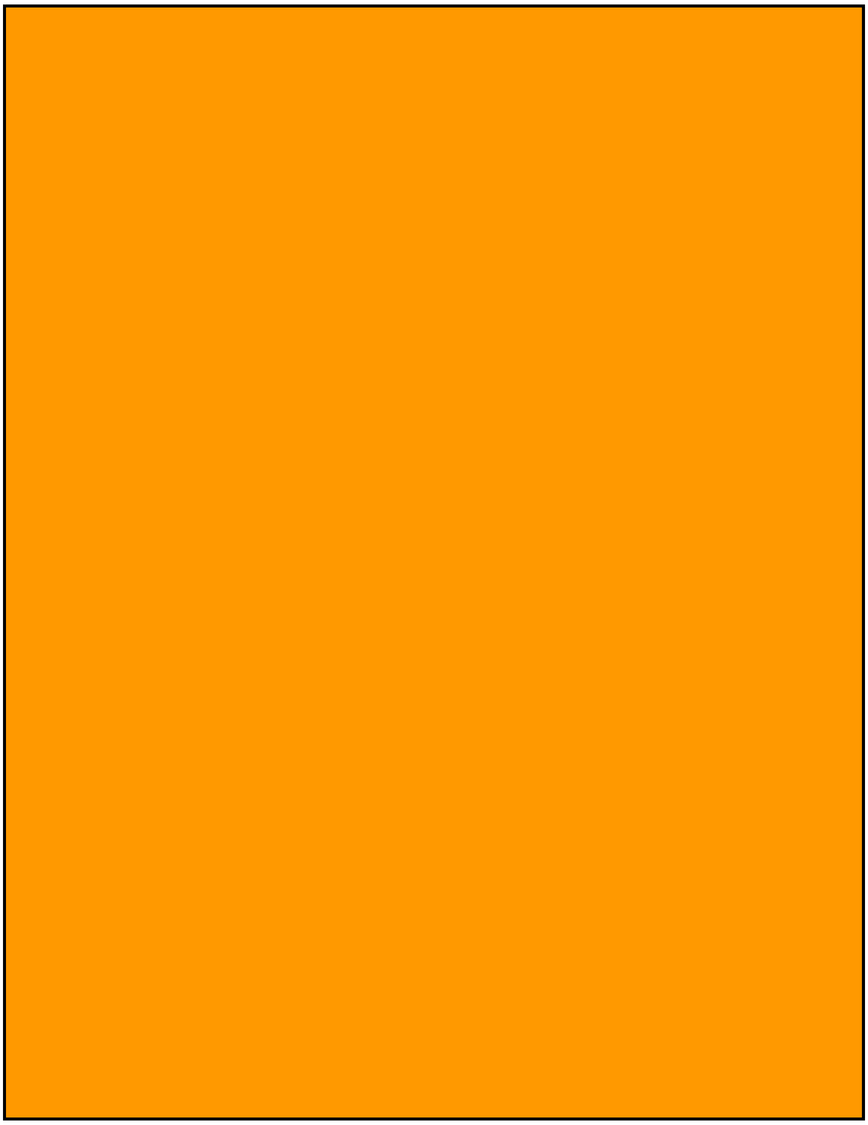

Summary of Quality Appraisal
•Although this study addressed a clearly focussed issue the external validity is limited by the exclusion criteria of patients who were living with relatives, those in unsupervised accommodation,and those in accommodation where staff were unwilling to engage. This introduces bias regarding the influence of staff factors in this process which has not been accounted for in this study.<br>•In addition there appear to be some informal exclusion criteria. Patients excluded tended to be on higher doses of APs, on PRNs and on other psychotropics ie polypharmacy. However as these were not definitive exclusion criteria they would need to be considered confounding factors within this study.
•Weak assocoaition between intervention and outcomes.<br>•no information on baseline cardiac function prior to the prescribing of thioridazine or mesoridazine<br>•unclear if any patients were switched to other psychotropic medicines, whether PRN medicines were prescribed and administered ,<br>•Authors identified confounding factors. Added factor was that tapering schedules were patient specific<br>Evidence from this cohort study not sufficiently robust to impact on clinical practice or policy making.

•This study does contribute to the evidence base but larger studies in a variety of settings are needed,<br>•It is not suficiently robust and many of the cofounding factors have been identified by the authors.Other confounding factors include behavioural, environmental and other psychotropic medication interventions as well as PRN prescribing and administration.<br>•It is unclear how the cohort was recruited and the selection critera were applied which could have introduced bias.<br>•There were difficulties in recruitment and the fear of worsening behaviour were main reasons why clinicans, care givers and legal reps did not wsh to particiapte.<br>•These factors introduce sampling bias
Study addressed a clearly focussed question<br>sufficient numbers of participants recruited<br>follow up period longer than other similar studies<br>confounding factors: behavioural, psychological interventions, other psychotropic medicines

•The study looking at the effects of a pharmacist on the prescribing of psychotropic medicines was limited in regard to the effects on indivual patients of the deprescribing.<br>• It was unclear what constituted successful deprescribing and the effect on CB.<br>• Inconsistent in how long indiviuals were followed up.

•Although this study is not sufficnetly robust in its methodology as confounding factors have not been accouinted for, it does provide some evidence of the rationale for minimum effective dosing which can implemented into clinical practice.

•Economic outcomes are focussed on medicine relted activities and do not account for other interventions that are required to support deprescribing.

• confounding factors - environment, behavioural interventions, staff related factors, other psychotropic medicines, PRN medicine

•This qualitative reflection on clinical practice used appropriate methodology to demonstrate subjective experiences of the researcher, backed up by some qualitative data.

•As the author / researcher is also the pharmacist delivering the STOMP intervention this could be a source of bias.

•Valuable study that will contribute to current clinical practice

In the process of patient follow-up, a telephone interview was carried out with the principal caregiver (n=76) and the ABC was administered (n=36). The follow-up ABC was attempted for all individuals answering the probe questions, however, a smaller amount of caretakers (n = 76) were willing or able to complete this follow-up.
Problems that interfered with completion of follow-up resulted from:
1) change in patient residence during the follow-up period resulting in staff who did not have the benefit of observations of a person over time and
2) unavailability of staff who could respond to questionnaires or telephone calls.
when the recommendations of the IDT had been followed, nearly 73% of caretakers reported that the patient showed improved behavior. In contrast, for the 43 subjects for which recommendations were not followed, approximately 19% reported improvement.

- selection bias
- missing baseline information
- intervention poorly defined

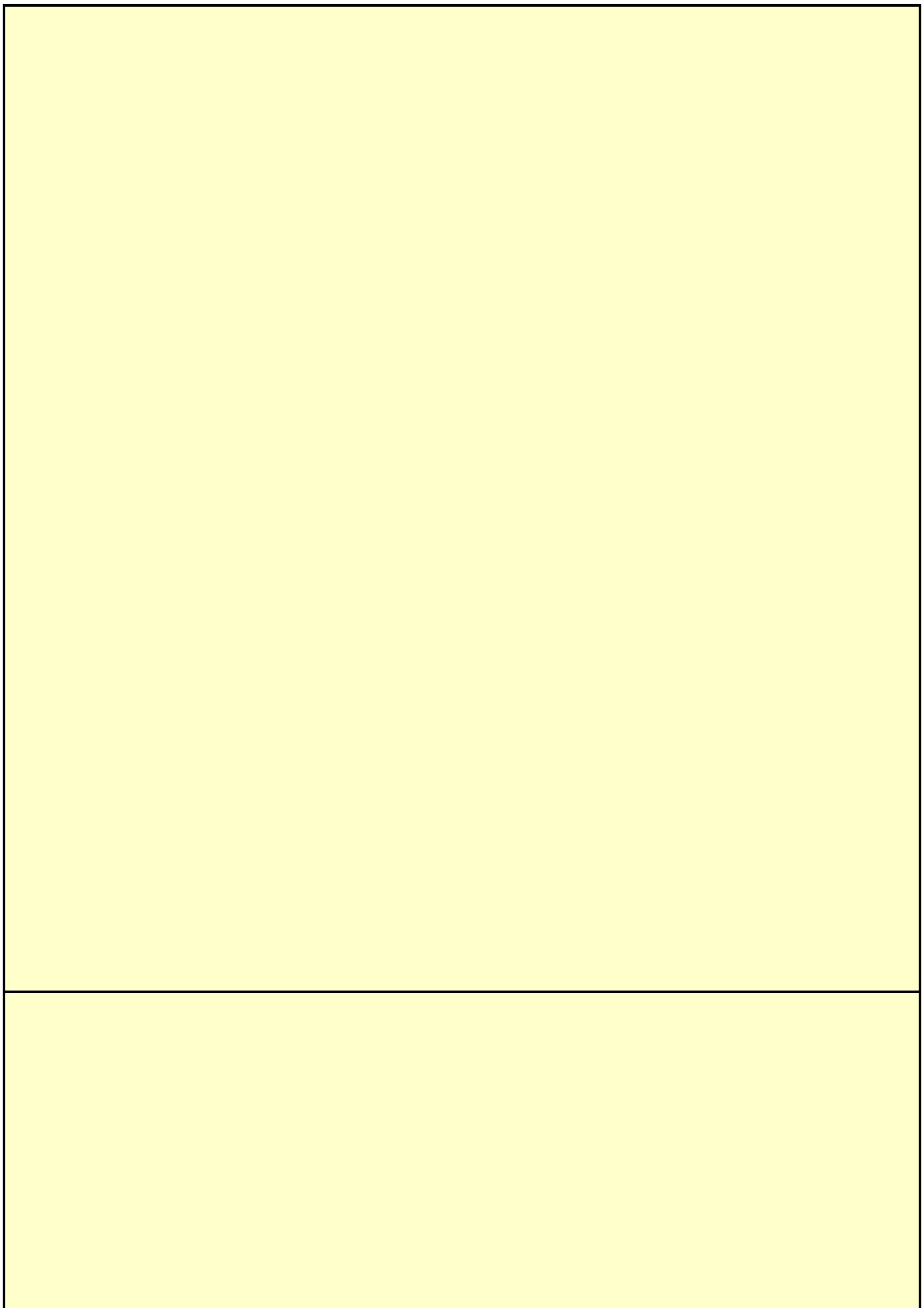

Details on methodology were missing which imapcted on ability to assess bias.
The participants had been part of several different industry sponsored studies and this study followed them afterwards.
The small number of participants, different weighing scales, using weight as an outome rather than BMI and missing data impacted on the reliability of this study.
Confounding factors of dietary modifcation, environmental and behavioural interventions

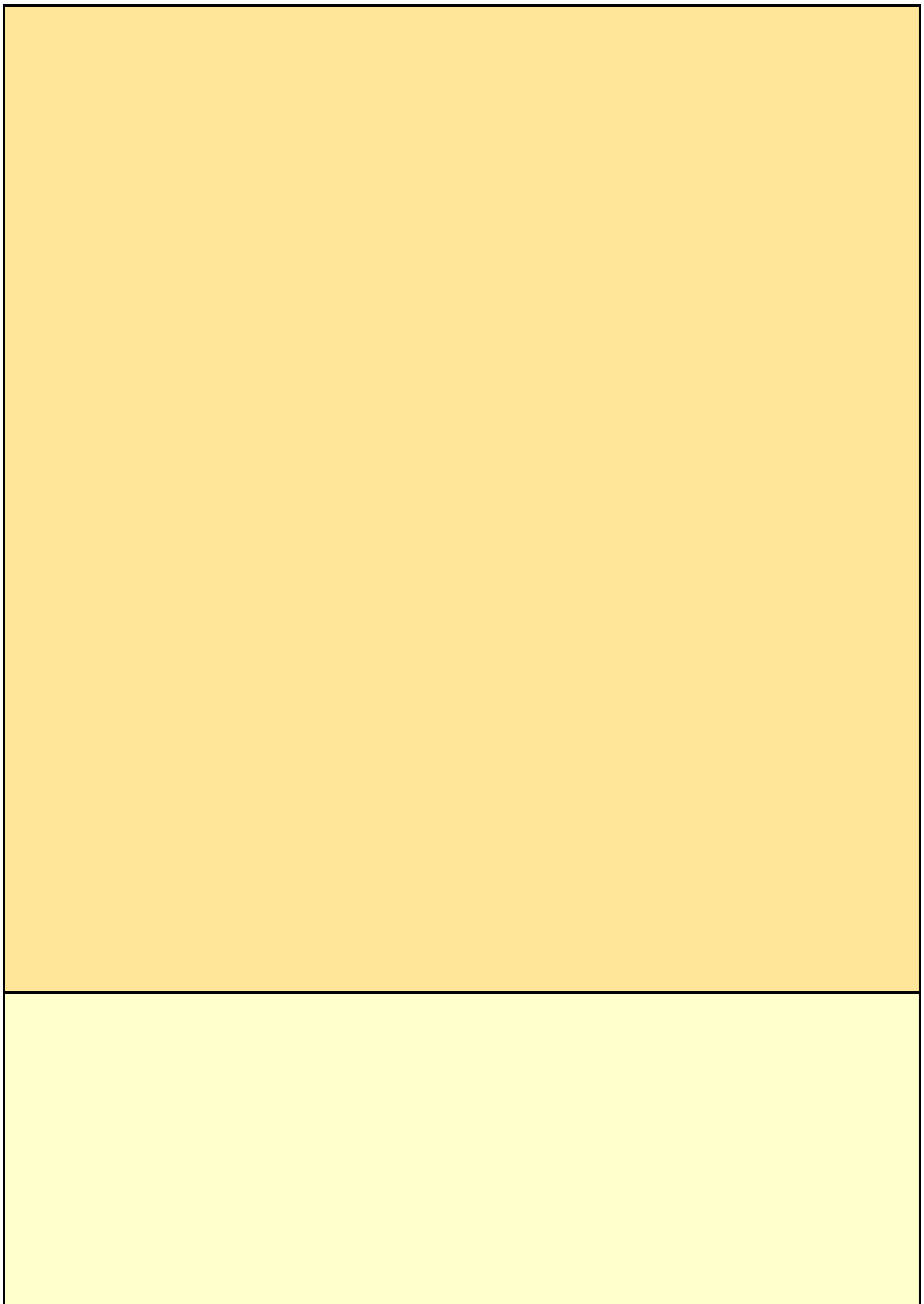

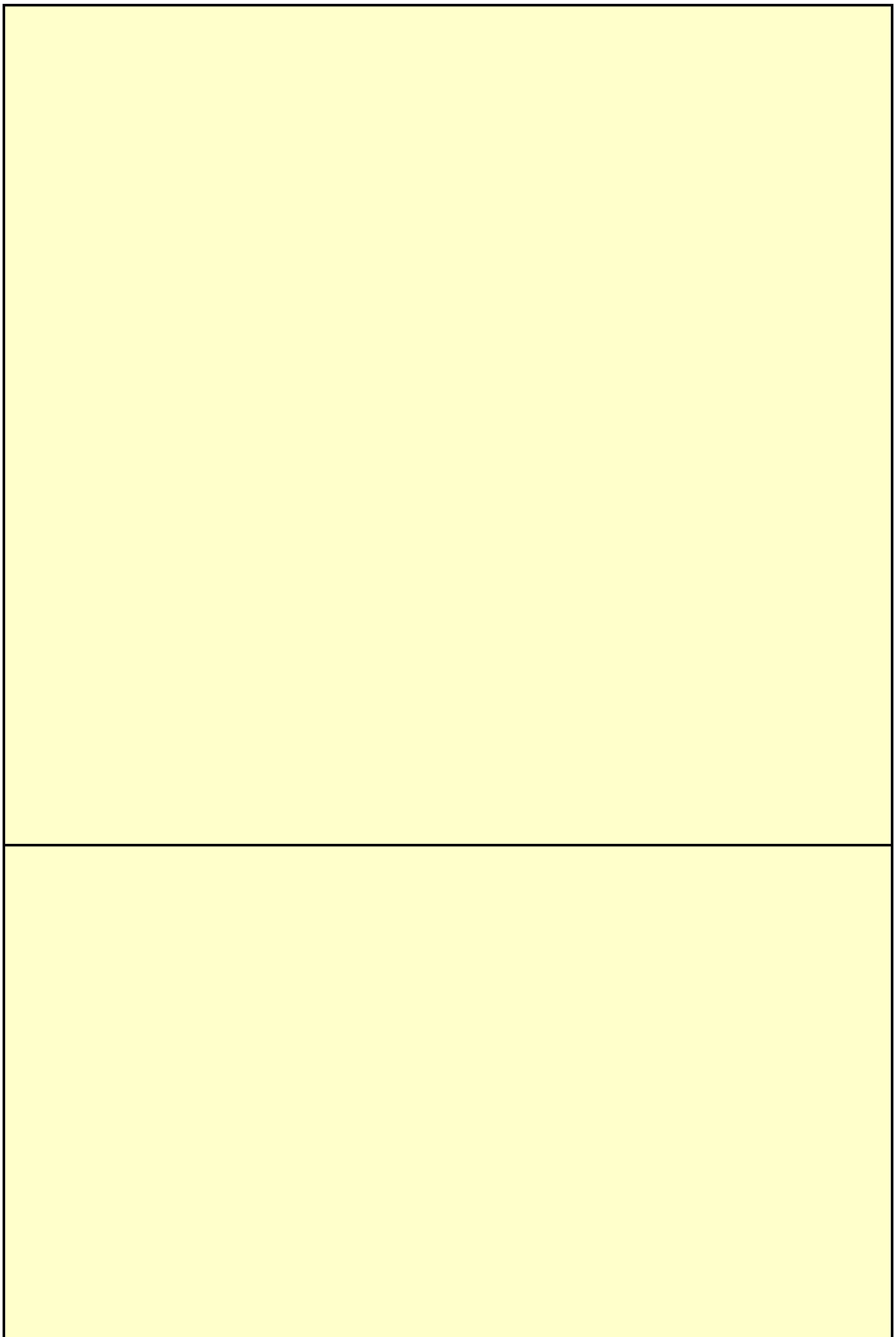

ıd therefore this would be a limiting factor for inclusion.

Overall this study has good internal and external validity with the authors comprehensively identifying limiting factors.
In addition consideration was not given to the the co prescribing of other psychotropic medicines and the prescribing and administering of PRN meds.
Length of follow up was unclear if it was three moinths after the discontinuation process was completed or three months after the beginning of the process.

•This study addressed a focus question and the cohort was representative of a defined population
•Methodology was weak with no standardised outcome measurement tools and relying on subjective measurements
•The authors did not identify any confounding factors .
•Could be detection bias as it was the clinician who decided how to reduce the thioridazine
•Confounding factors include: no mention of behavioural interventions, environmental factors from the different settings, additional APs and switches to APs not specified, other psychotropic meds prescribed, PRN prescribing and administration, clinician decided on reduction or switch method, study included people with known diagnoses of mental illness eg psychosis,
 schizophrenia, length and extent of follow up unclear
•Results are unclear withn the weak association that APs are effective in managing CB.
•Unclear as narrative seems to mix between withdrawal of thioridazine and withdrwal of APs.
variety of residential settings was representative but could have had an impact on results.
•Although the study methodology is not sufficintly robus, it does raise some important isuues about dperescribing, in effect highl;ighting that deprescribing in isolation may not be the optimal methhod to reduce AP prescribing.

extra info

outcomes data

.

We indeed found significant differences in staff-related variables between those participants who succeeded and those who failed in complete discontinuation. Also, we found a number of staff-related factors that were associated with the chance of complete discontinuation. Male gender and feelings of depression/anger of support professionals, and clinicians' judgements of worsening in behaviour during the discontinuation trajectory were associated with a lesser chance of complete discontinuation, and clinicians' judgements of no worsening in behaviour with a higher likelihood. Furthermore, we found support professionals' education and knowledge of psychotropic drug use, and agreement in staff with regard to participants' eligibility to discontinue the antipsychotic drug use were positively associated with successful discontinuation. Remarkable findings were the association of support professionals' feelings of "cheerful/excited" and "confident/relaxed" with failure and the association of clinicians' judgement of higher baseline severity of maladaptive behaviour with success in discontinuation at 16 weeks. We found a number of weak correlations and no moderate and strong correlations between support professionals-related questionnaires and ABC scales at baseline and at 16 weeks:
We found no correlations of clinicians' judgements of the severity of behavioural symptoms as assessed with CGI-S and change in behaviour as assessed with CGI-I, with any of ABC scales at baseline and 16 weeks.

strong correlations between support professionals-related questionnaires and ABC scales at baseline and at 16 weeks:

We found no correlations of clinicians' judgements of the severity of behavioural sym

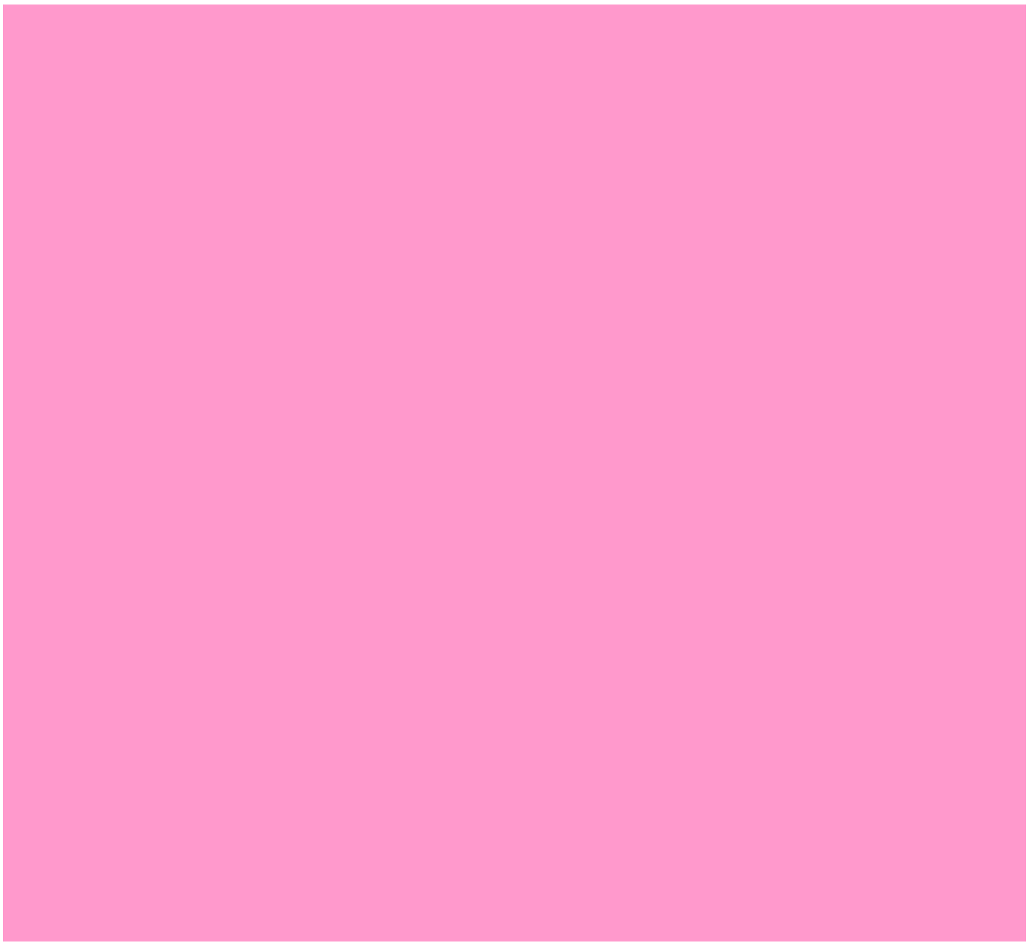

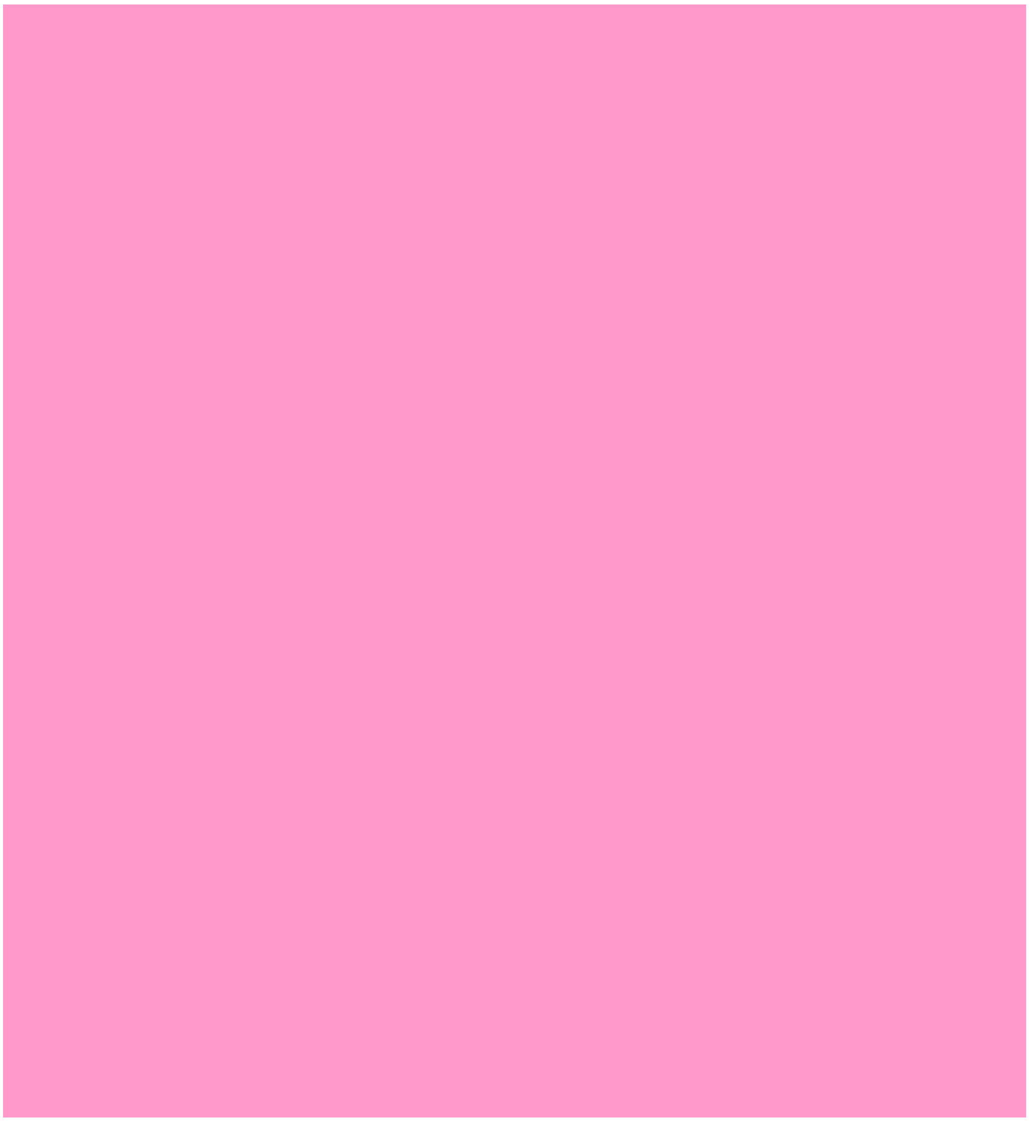

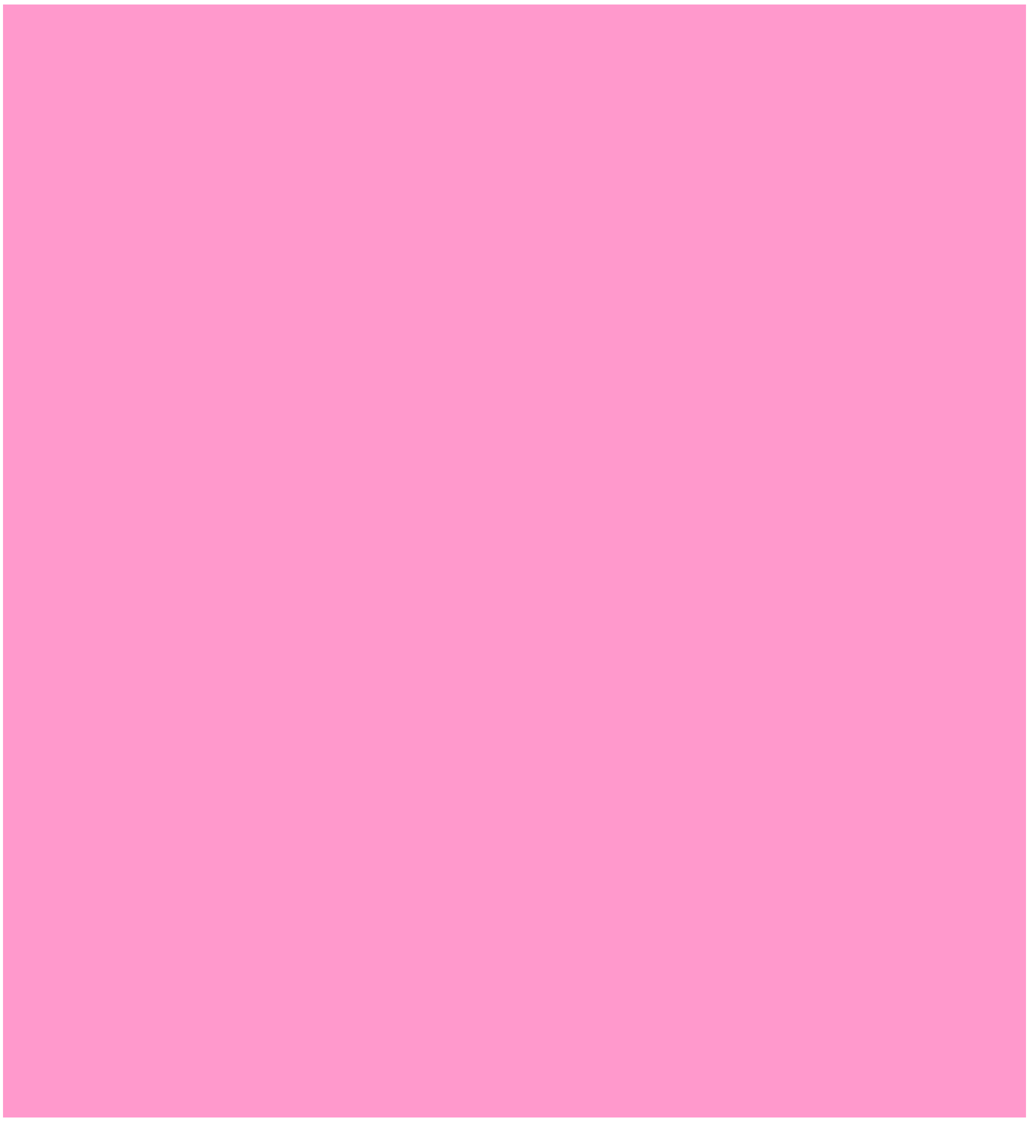

this observational study is not sufficiently robust to recommend chnages within clinical practice. However the benefit of the IDT is supoorted by evidence from other published service evaluations. Confunding factors include differing referral rates from the various primary care providers, poor follow up rates and the unreliability of the 3 month post clinic follow up. It is somewhat arbitary as to whether this is 3 months post change in prescribing as this could have happened at any time point within the 3 months.

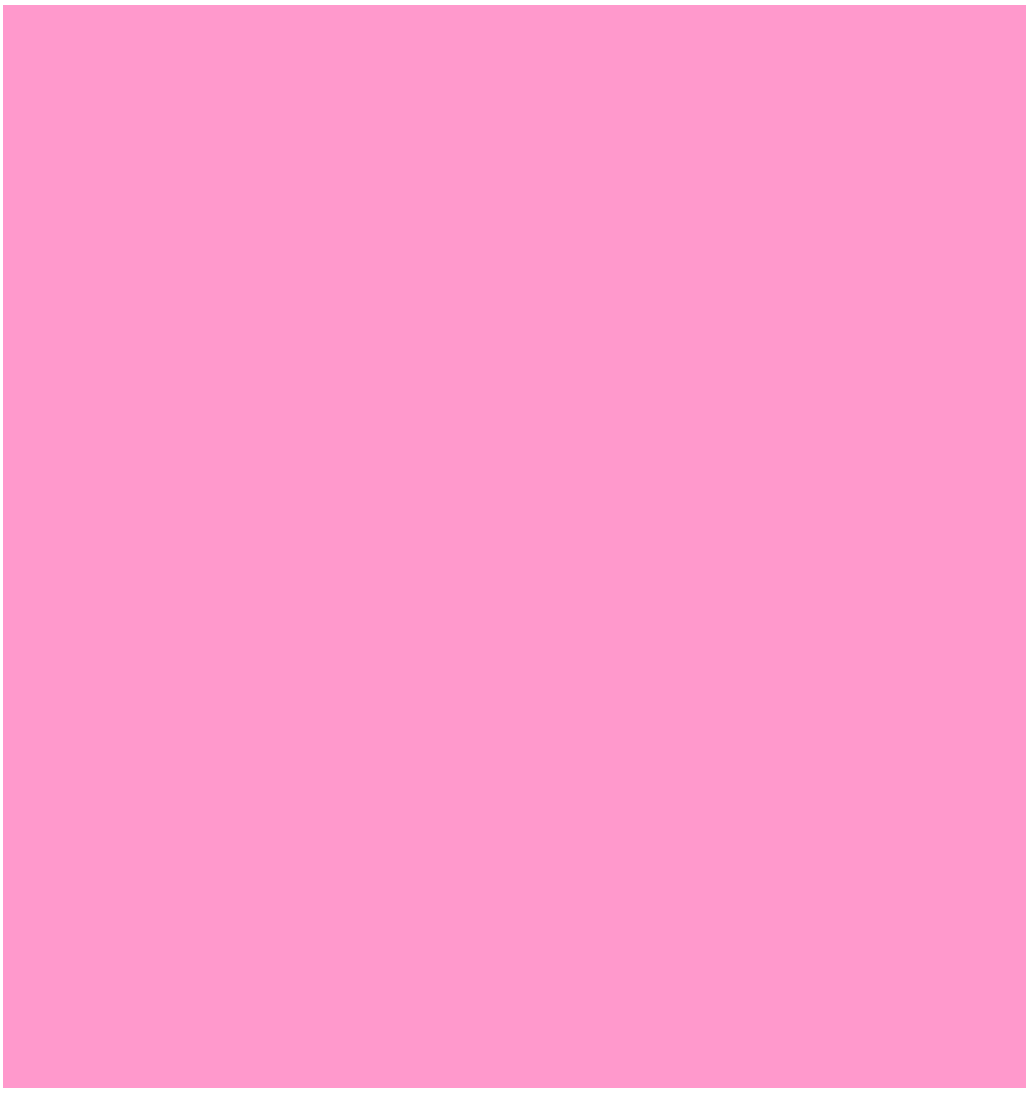

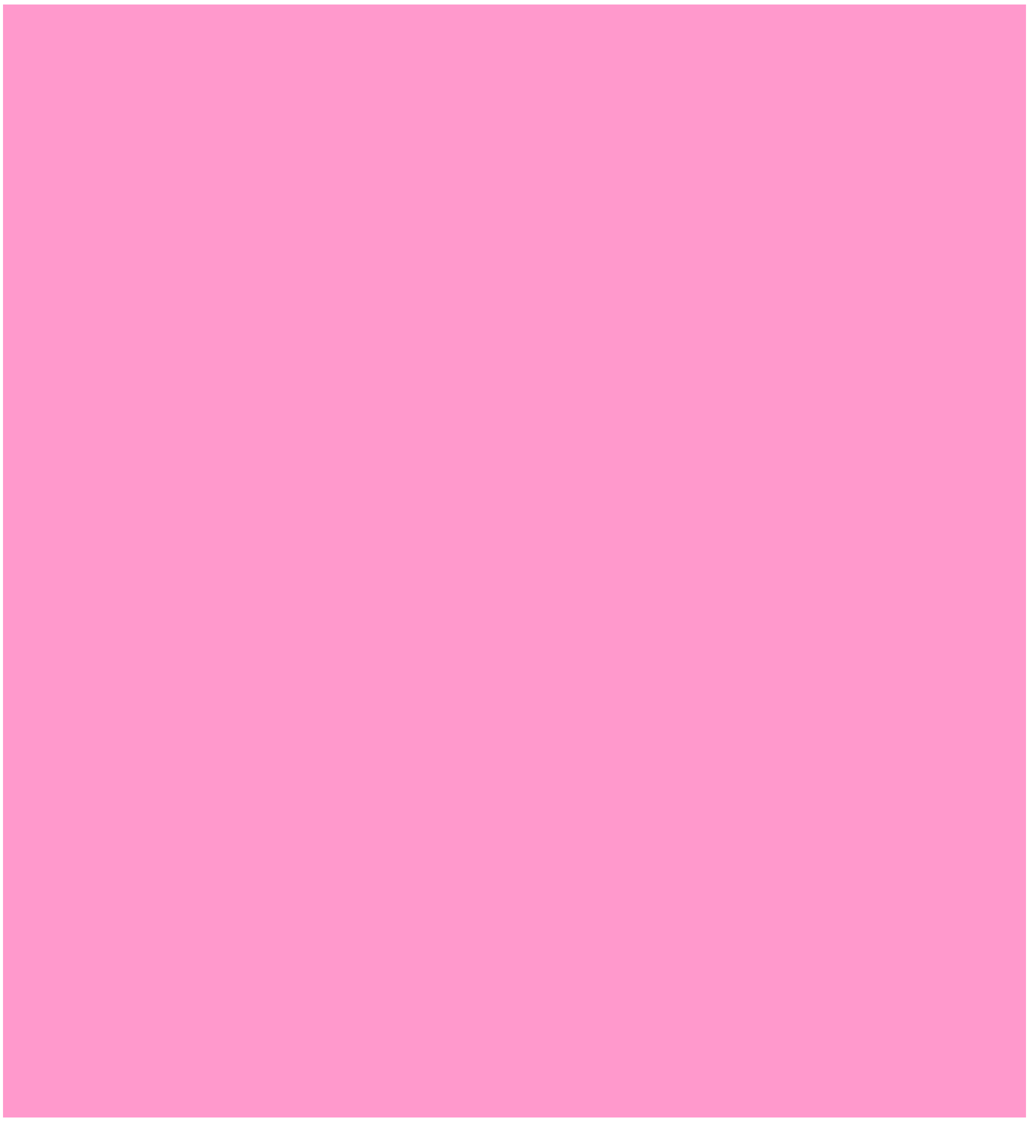

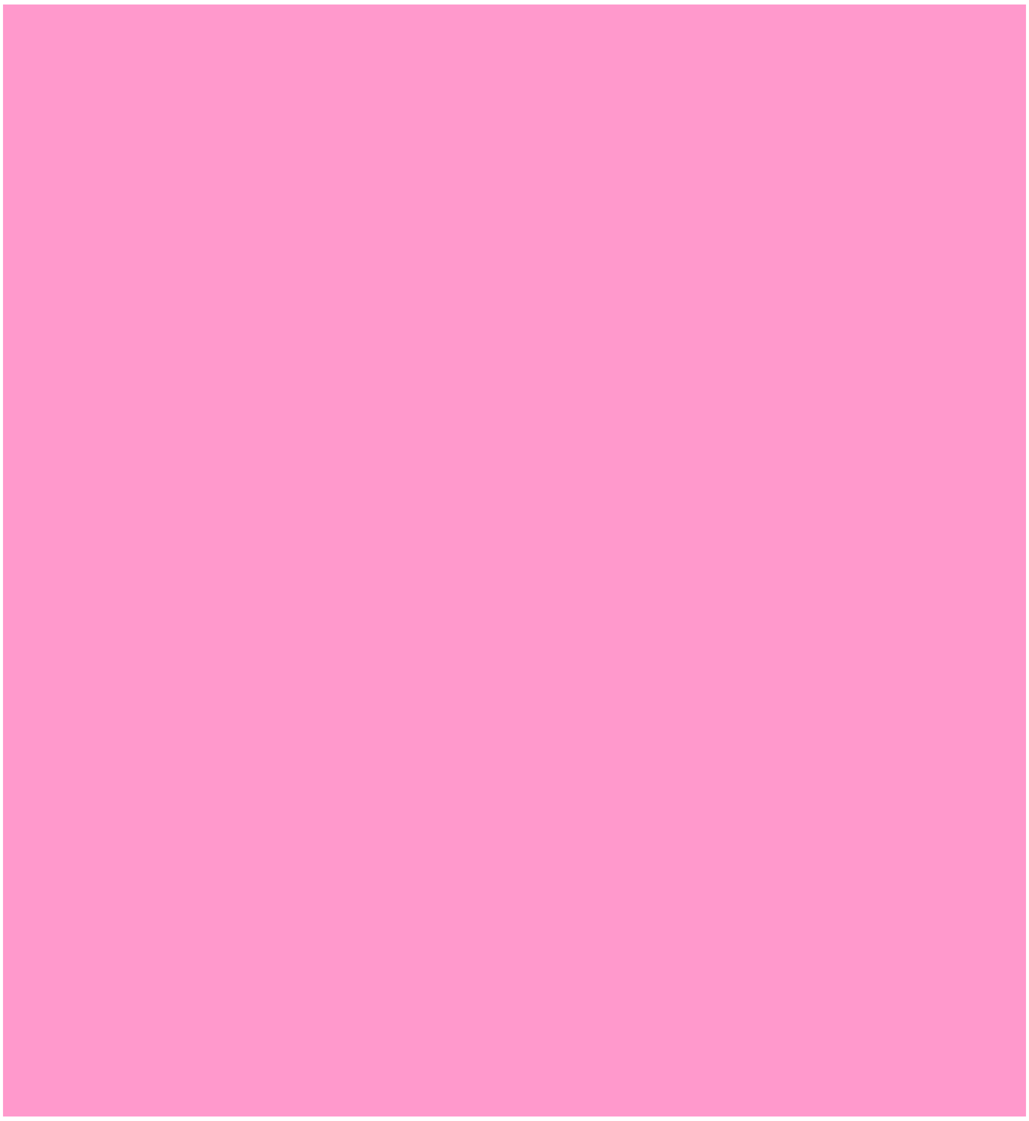

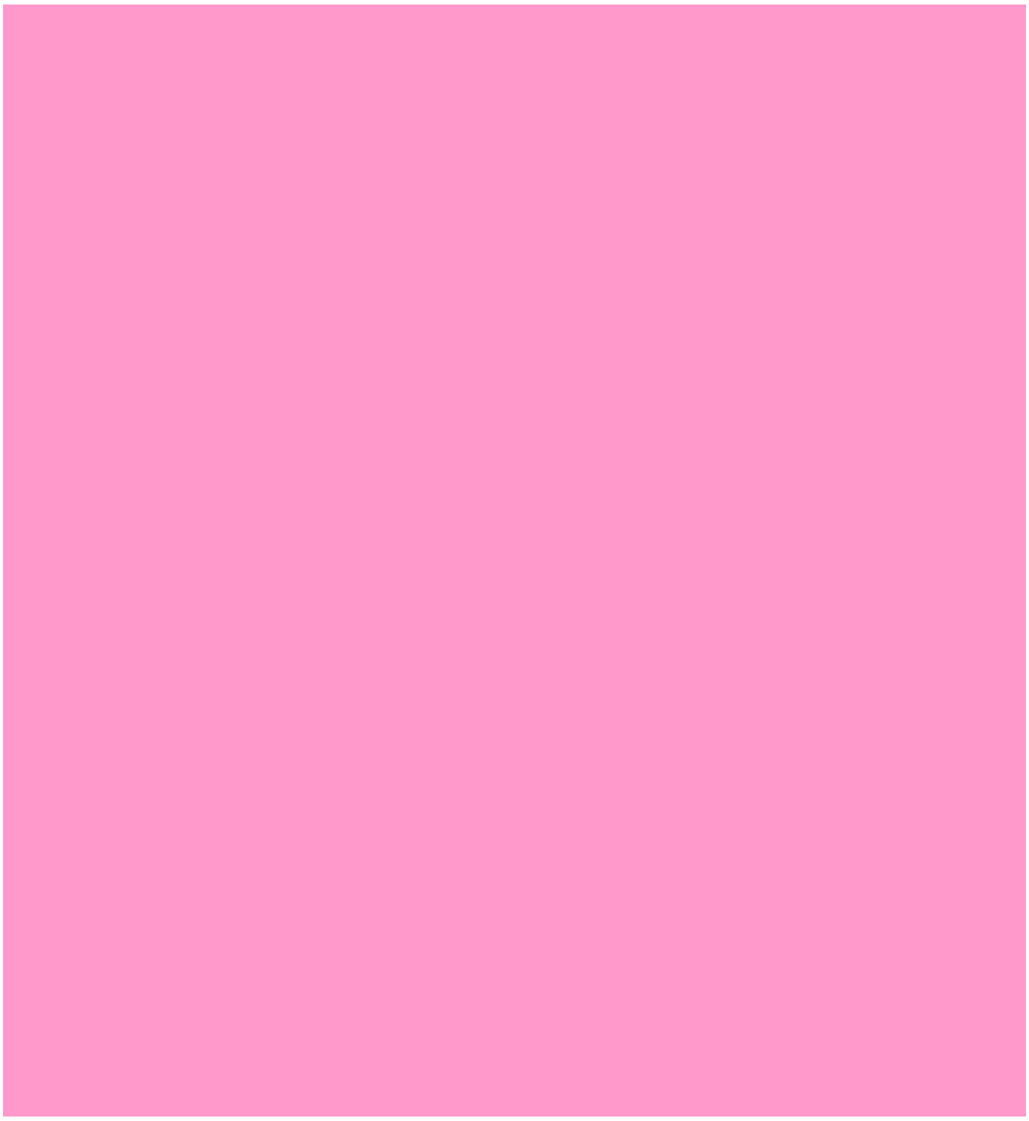

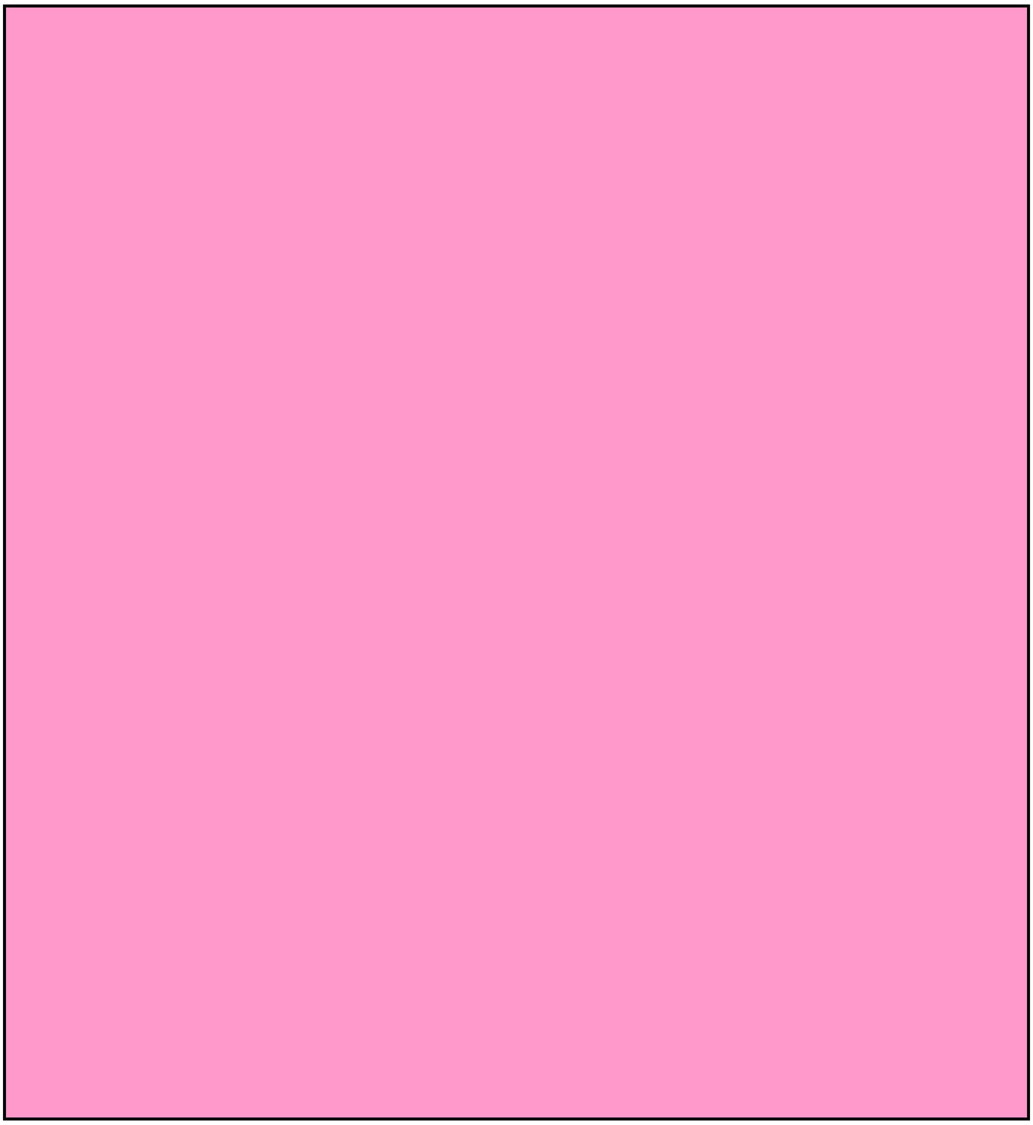

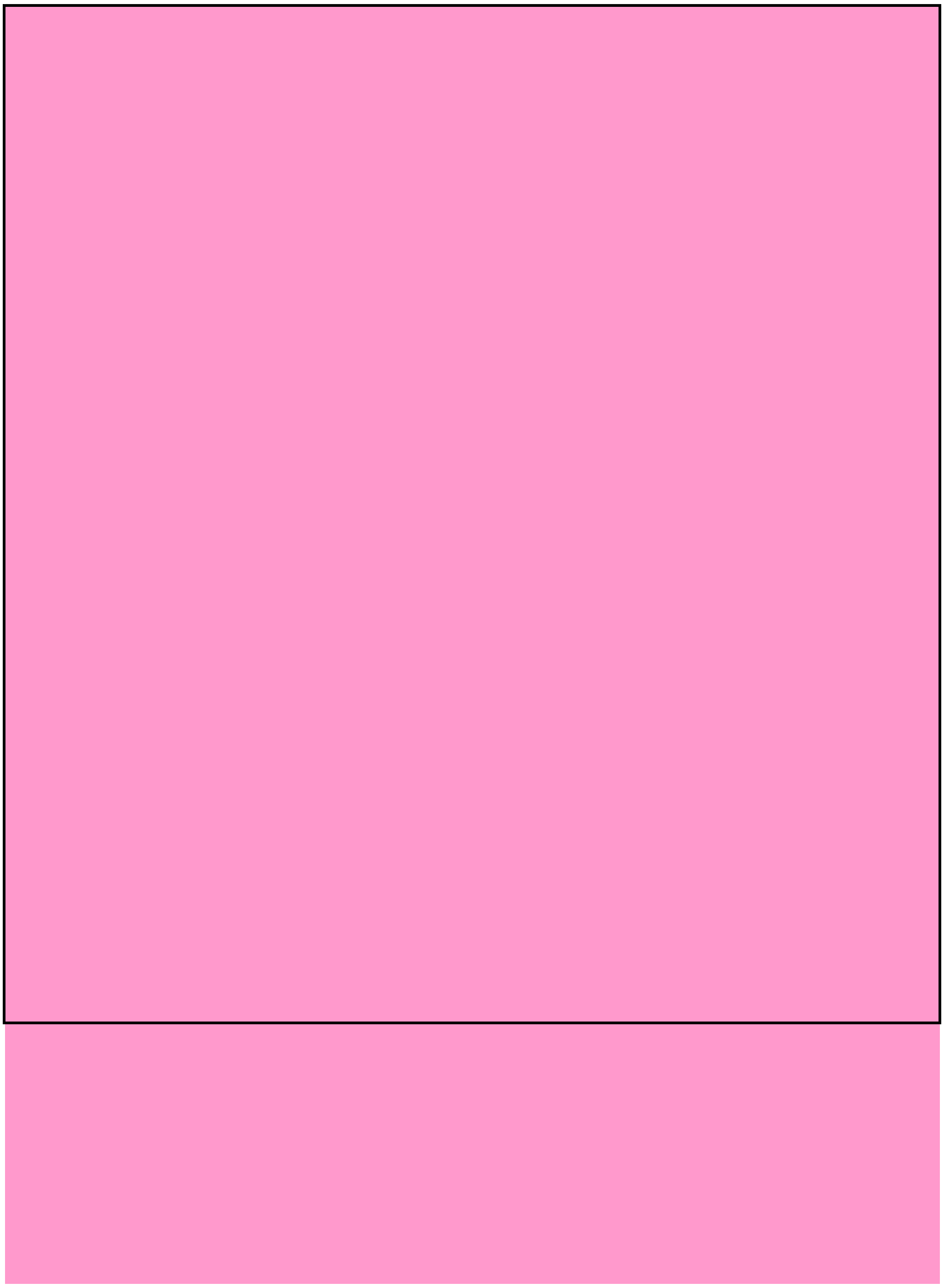

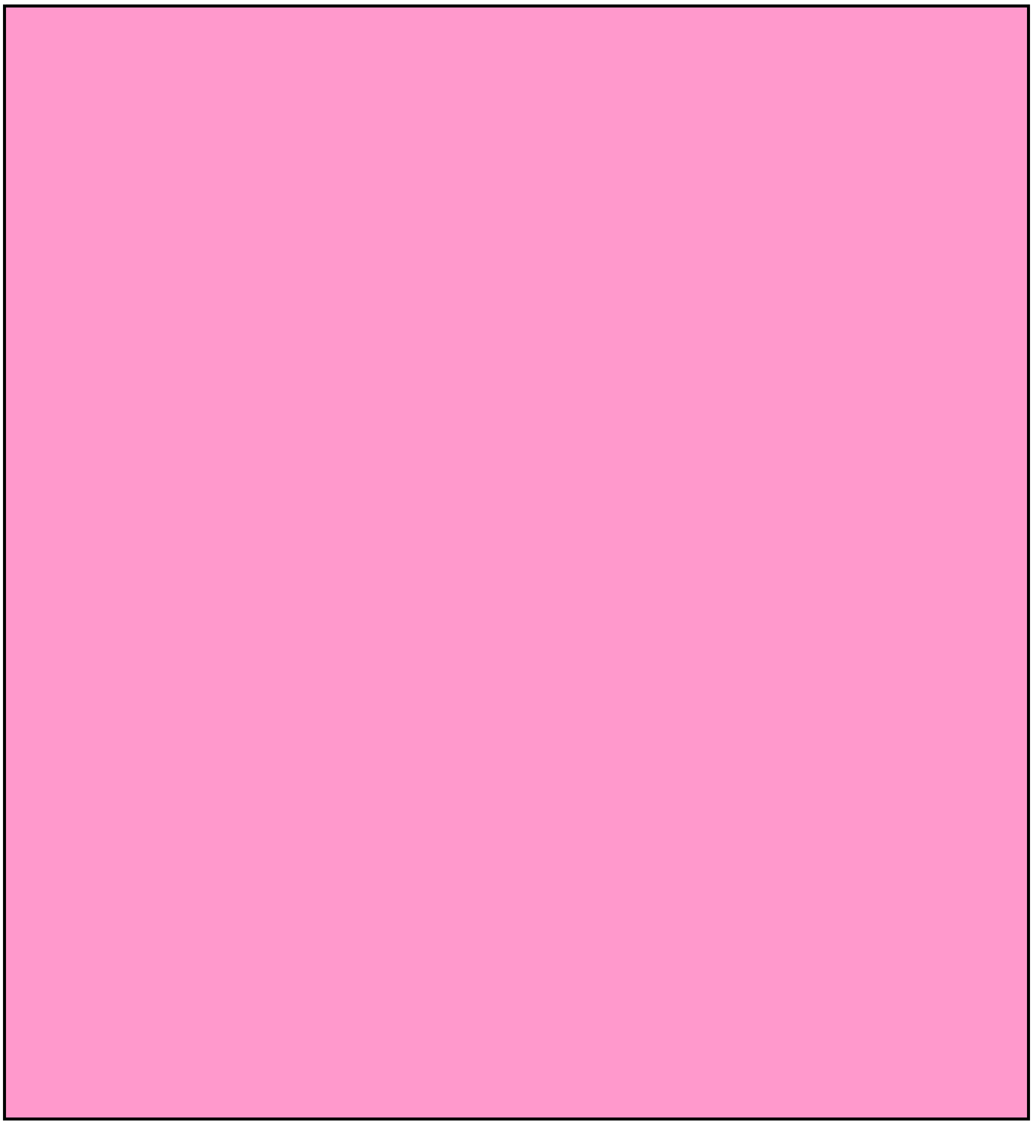

ıptoms as assessed with CGI-S and change in behaviour as assessed with CGI-I, with any of ABC scales at basel

line and 16 weeks.
